# Supplementary material for: Sequence and chemical specificity define the functional landscape of intrinsically disordered regions
Source: Nat Cell Biol. 2026 Feb 12;28(2):323–37. doi: 10.1038/s41556-025-01867-8 (PMC12904797; doi:10.1038/s41556-025-01867-8)
Supplement: Supplementary file 1 — Supplementary Figs. 1–15. [file 41556_2025_1867_MOESM1_ESM.pdf]

nature cell biology

Supplementary information https://doi.org/10.1038/s41556-025-01867-8

Sequence and chemical specificity define the functional landscape of intrinsically disordered regions

In the format provided by the authors and unedited

Supplementary Figures Table of Contents:

Supplementary figure 1 (Fig. S1) Page 2-40

Supplementary figure 2 (Fig. S2) Page 41-62

Supplementary figure 3 (Fig. S3) Page 63

Supplementary figure 4 (Fig. S4) Page 64

Supplementary figure 5 (Fig. S5) Page 65

Supplementary figure 6 (Fig. S6) Page 66

Supplementary figure 7 (Fig. S7) Page 67-68

Supplementary figure 8 (Fig. S8) Page 69

Supplementary figure 9 (Fig. S9) Page 70

Supplementary figure 10 (Fig. S10) Page 71

Supplementary figure 11 (Fig. S11) Page 72

Supplementary figure 12 (Fig. S12) Page 73

Supplementary figure 13 (Fig. S13) Page 74

Supplementary figure 14 (Fig. S14) Page 75

Supplementary figure 15 (Fig. S15) Page 76

Supplementary Figure S1: **Viability of Abf1 constructs assessed by 5-FOA plasmid shuffling assay.**

Strains harboring both pRS416-*ABF1* plasmid (*URA3* marker) and pRS315-”*abf1* construct name” (*LEU2* marker) in the strain background with deleted *abf1* chromosomal gene were re-streaked from patches from YNB without uracil and leucine plates onto 5-FOA without leucine plates. Only constructs discussed in the paper are labeled in plate schemes, where names of viable strains are in blue, names of inviable ones in black. Viability on 5-FOA plates was visually scored by comparison to known viable or inviable strains on the same plate. Inviable strains were distinguished from viable strains because they did not show growth of a contiguous patch after at most three days of incubation at 30 °C but only a smeary appearance or at most sparse single colonies, whereas viable strains grew as a contiguously dense patch. If few individual colonies grew instead of a contiguous patch, we tested some of them by colony PCR with the primers listed in Table S8. In all cases, these individual colonies contained plasmids of a wrong size or sequence reflecting recombination between pRS416-*ABF1* and pRS315-”*abf1* construct name”.
For each construct, independent clones after transformation were tested. On each slide, a table summarizes the result of the shown plate and in brackets the total result obtained for this construct if all tested clones on all plates were considered. If the same clone showed conflicting results in technical replicates, it was not counted as viable or inviable but included in the total number of tested clones. The final categorization of each construct as viable or inviable followed by the majority of tested clones excluding the clones with ambiguous results in technical replicates.

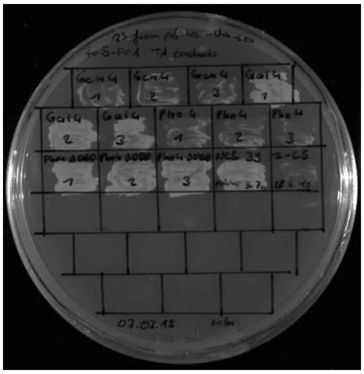

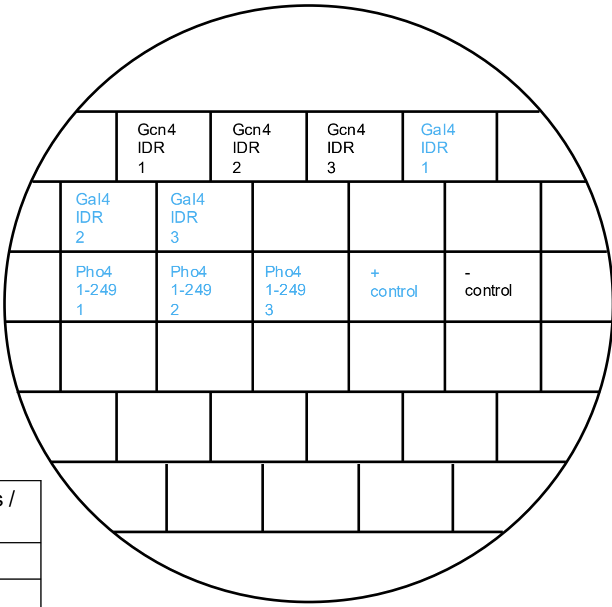

Restreak from YNB –ura –leu to 5-FOA –leu

Construct	Result regarding viability on 5-FOA	Number of (in)viable clones / total number of clones
Gcn4$^{17-150}$	inviable	3 out of 3
Gal4$^{768-881}$	viable	3 out of 3
Pho4$^{1-249}$	viable	3 out of 3

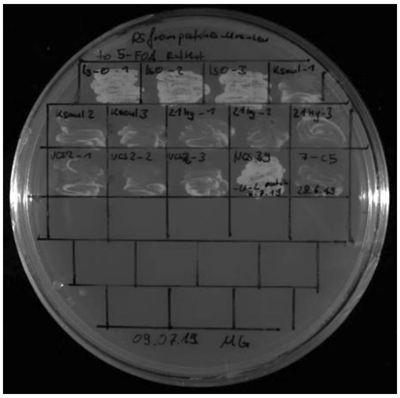

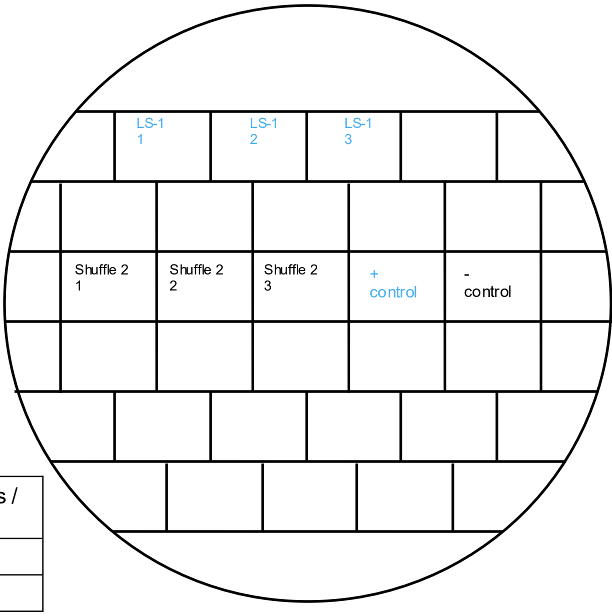

Restreak from YNB –ura –leu to 5-FOA –leu

Construct	Result regarding viability on 5-FOA	Number of (in)viable clones / total number of clones
LS-1	viable	3 out of 3
Shuffle 2	inviable	3 out of 3

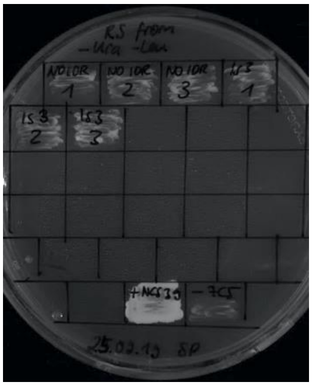

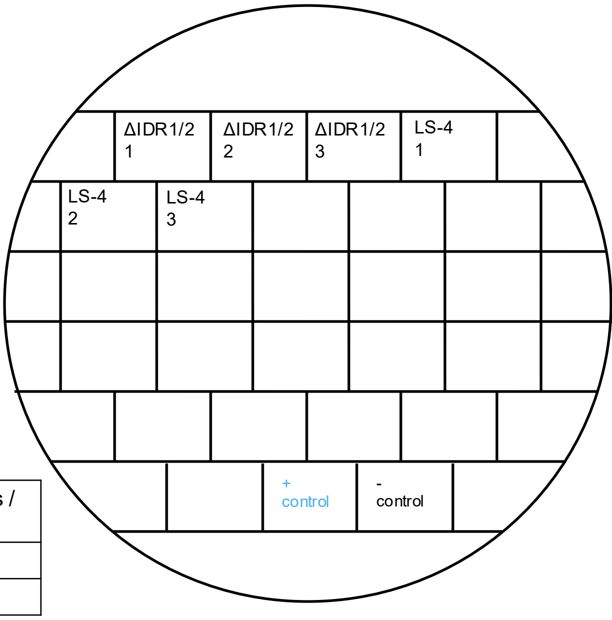

Restreak from YNB –ura –leu to 5-FOA –leu

Construct	Result regarding viability on 5-FOA	Number of (in)viable clones / total number of clones
ΔIDR1/2 (NLS-FLAG)	inviable	3 out of 3
LS-4	inviable	3 out of 3

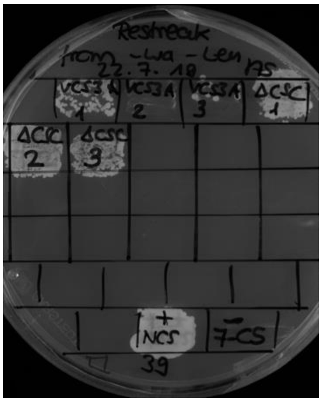

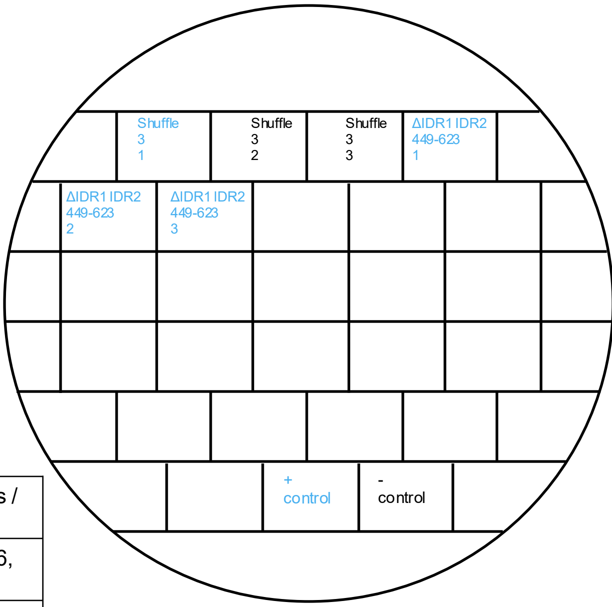

Restreak from YNB –ura –leu to 5-FOA –leu

Construct	Result regarding viability on 5-FOA	Number of (in)viable clones / total number of clones
Shuffle 3	inviable	2 out of 3 (in total 5 out ot 6, see also slide 5)
ΔIDR1 & IDR2$^{449\text{-}623}$	viable	3 out of 3

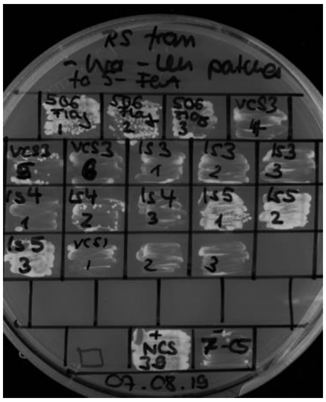

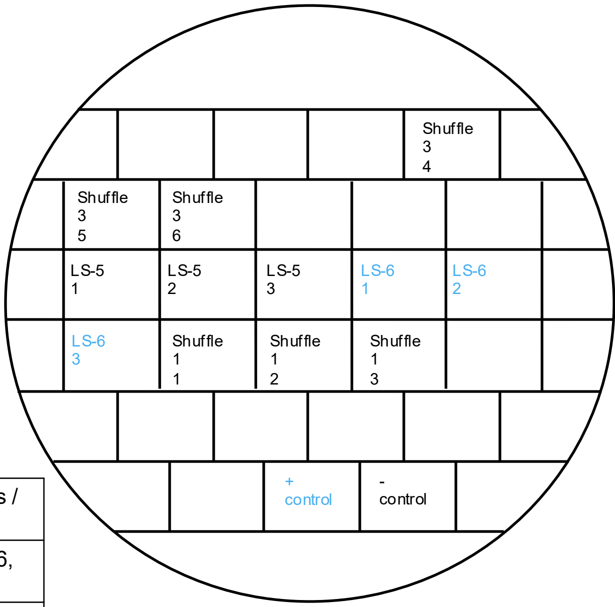

Restreak from YNB –ura –leu to 5-FOA –leu

Construct	Result regarding viability on 5-FOA	Number of (in)viable clones / total number of clones
Shuffle 3	inviable	3 out of 3 (in total 5 out ot 6, see also slide 4)
LS-5	inviable	3 out of 3
LS-6	viable	3 out of 3
Shuffle 1	inviable	3 out of 3

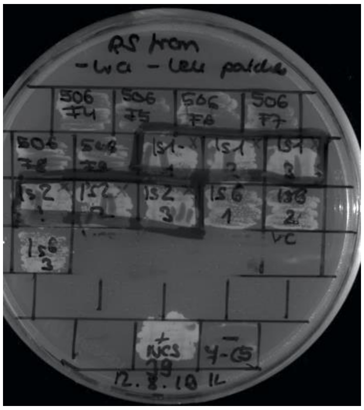

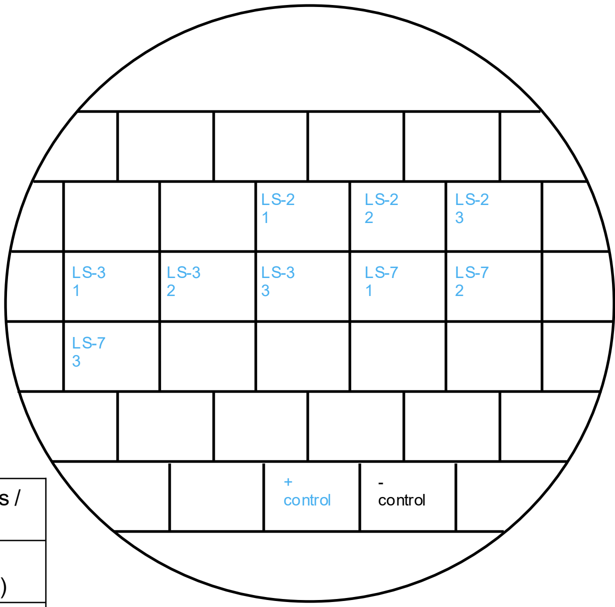

Restreak from YNB –ura –leu to 5-FOA –leu

Construct	Result regarding viability on 5-FOA	Number of (in)viable clones / total number of clones
LS-2	viable	3 out of 3 (technical replicates, see also slide 7)
LS-3	viable	3 out of 3 (technical replicates, see also slide 7)
LS-7	viable	3 out of 3 (in total 8 out of 8, see also slide 6)

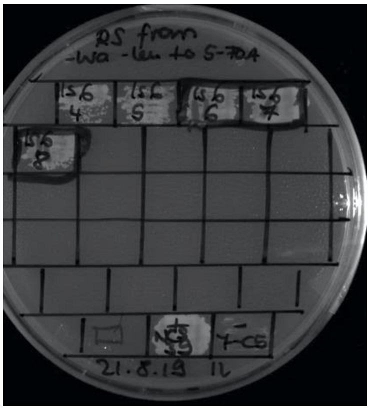

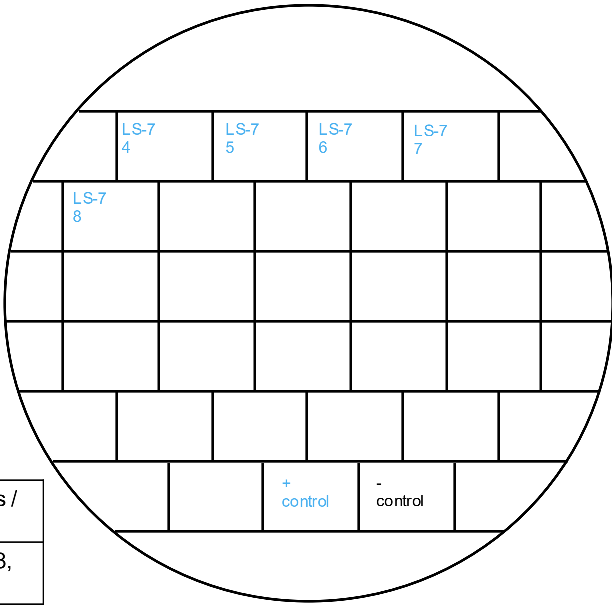

Restreak from YNB –ura –leu to 5-FOA –leu

Construct	Result regarding viability on 5-FOA	Number of (in)viable clones / total number of clones
LS-7	viable	5 out of 5 (in total 8 out of 8, see also slide 5)

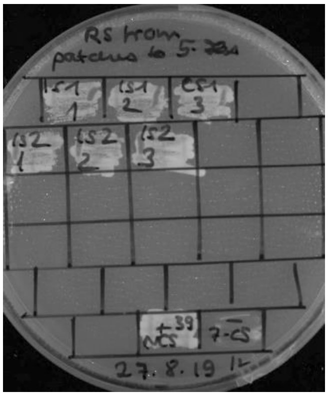

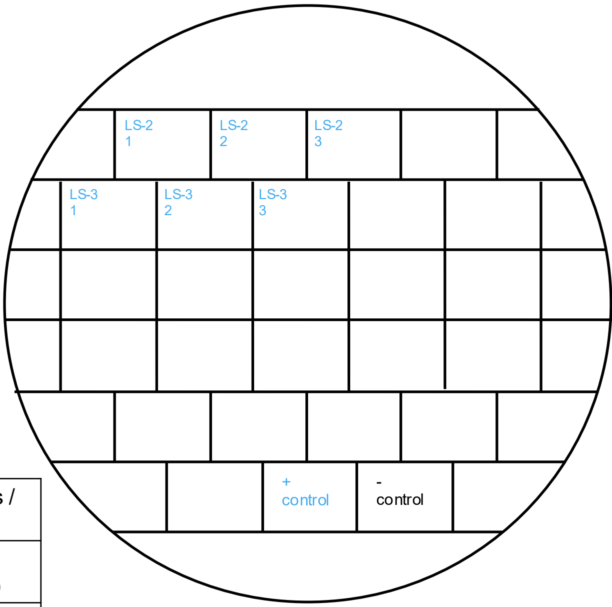

Restreak from YNB –ura –leu to 5-FOA –leu

Construct	Result regarding viability on 5-FOA	Number of (in)viable clones / total number of clones
LS-2	viable	3 out of 3 (technical replicates, see also slide 5)
LS-3	viable	3 out of 3 (technical replicates, see also slide 5)

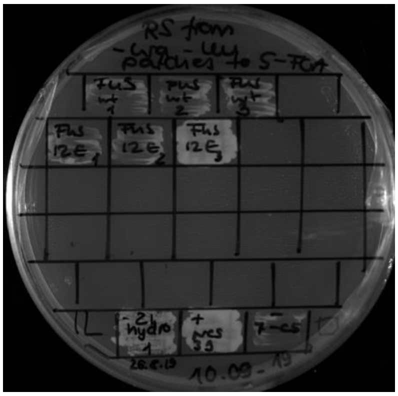

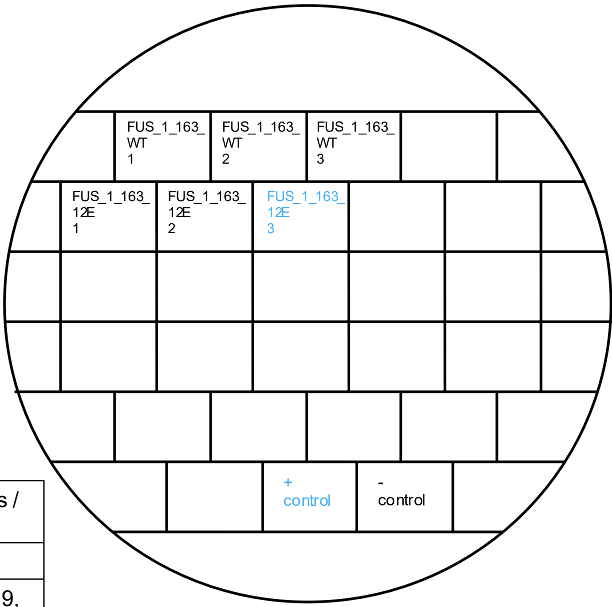

Restreak from YNB –ura –leu to 5-FOA –leu

Construct	Construct	Number of (in)viable clones / total number of clones
FUS$^{1\text{-}163}$	inviable	3 out of 3
FUS$^{1\text{-}163}$12E	inviable	2 out of 3 (in total: 8 out of 9, see also slides 8 and 15)

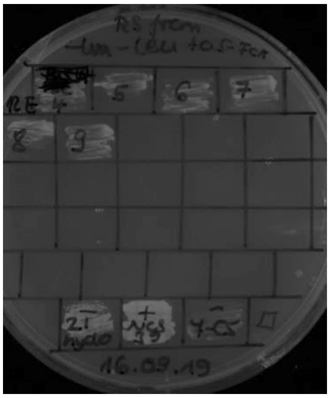

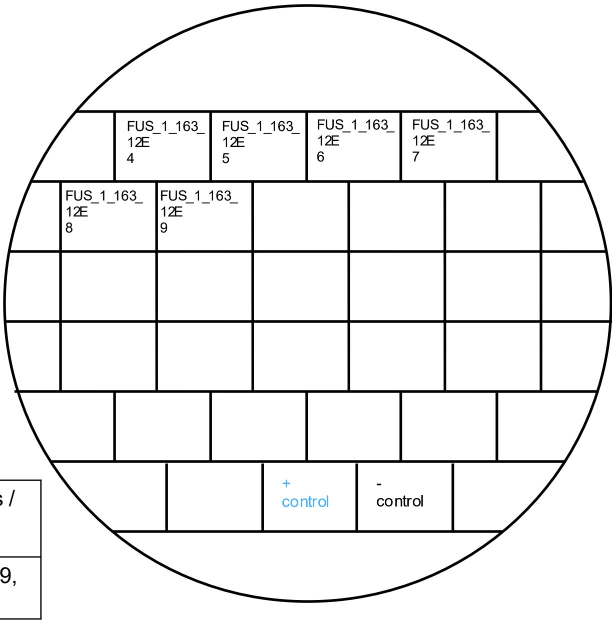

Restreak from YNB –ura –leu to 5-FOA –leu

Construct	Result regarding viability on 5-FOA	Number of (in)viable clones / total number of clones
FUS$^{1\text{-}163}$12E	Inviable	6 out of 6 ((in total 8 out of 9, see also slides 7 and 15)

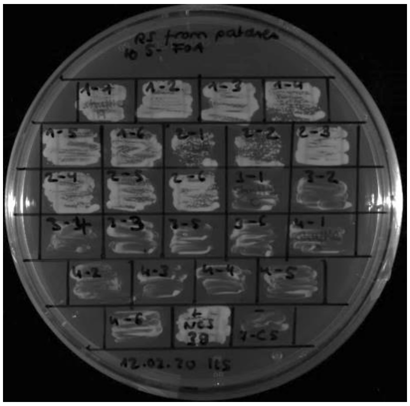

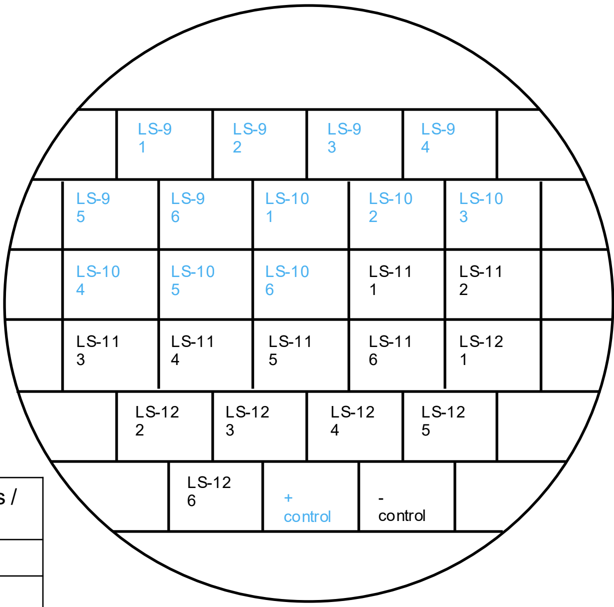

Restreak from YNB –ura –leu to 5-FOA –leu

Construct	Result regarding viability on 5-FOA	Number of (in)viable clones / total number of clones
LS-9	viable	6 out of 6
LS-10	viable	6 out of 6
LS-11	inviable	6 out of 6
LS-12	inviable	6 out of 6

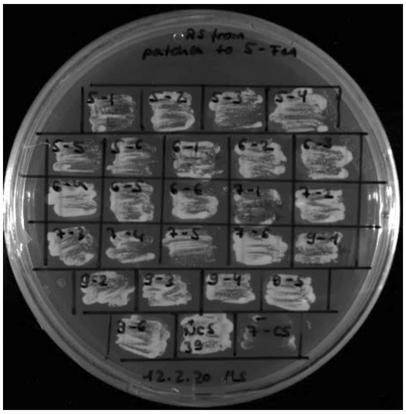

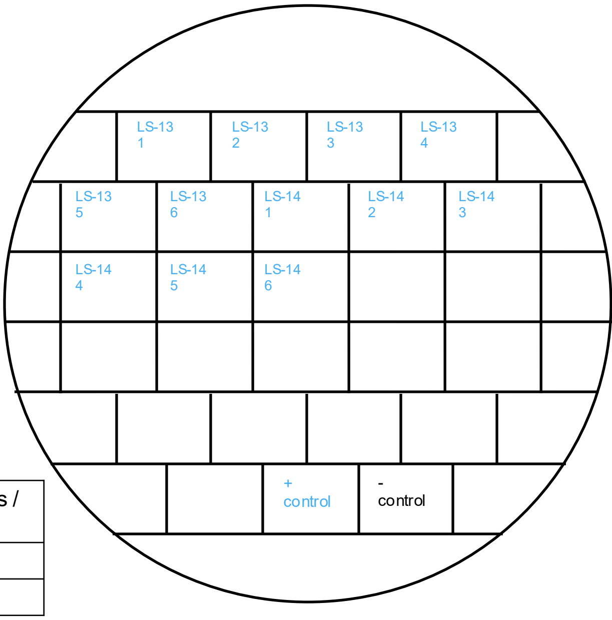

Restreak from YNB –ura –leu to 5-FOA –leu

Construct	Construct	Number of (in)viable clones / total number of clones
LS-13	viable	6 out of 6
LS-14	viable	6 out of 6

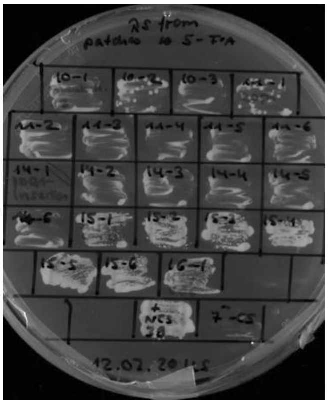

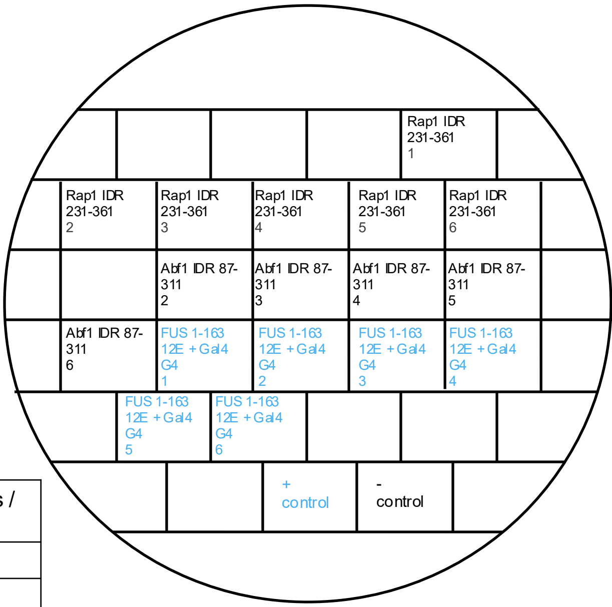

Restreak from YNB –ura –leu to 5-FOA –leu

Construct	Result regarding viability on 5-FOA	Number of (in)viable clones / total number of clones
Rap1$^{231-361}$	inviable	6 out of 6
Abf1 IDR1$^{87-311}$	inviable	6 out of 6
FUS$^{1-163}$12E + Gal4^{G4}	viable	6 out of 6

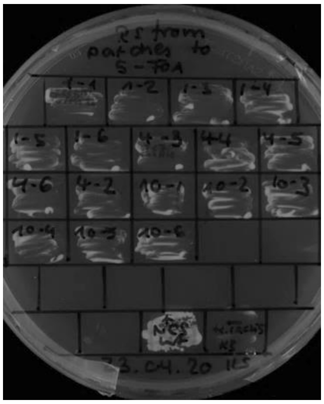

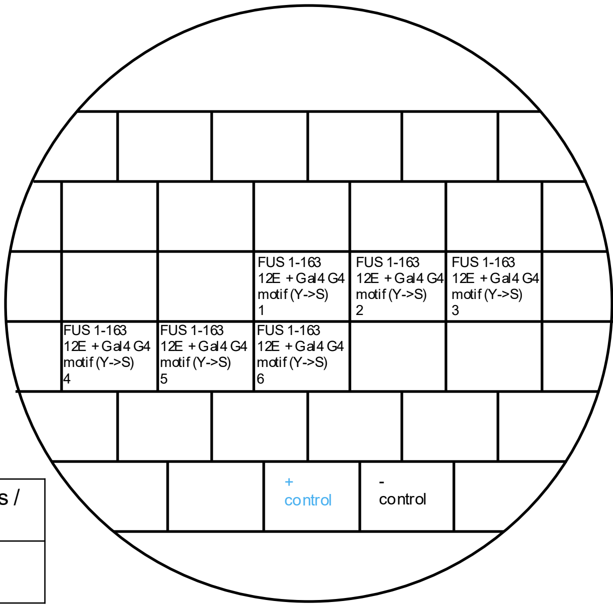

Restreak from YNB –ura –leu to 5-FOA –leu

Construct	Construct	Number of (in)viable clones / total number of clones
FUS$^{1\text{-}163}$12E + Gal4^{G4} Y→S in context	inviable	6 out of 6

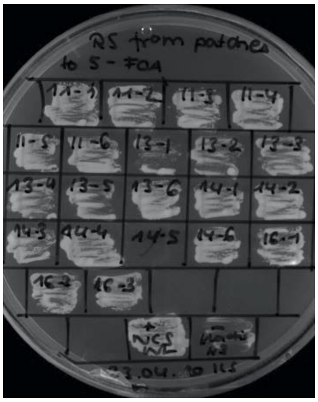

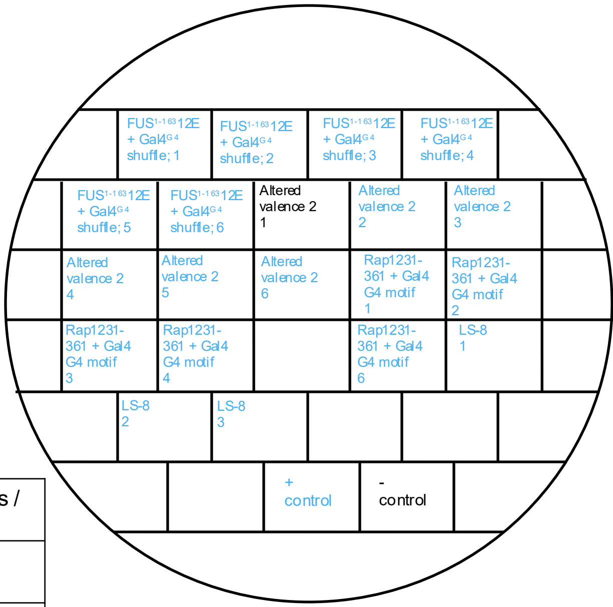

Restreak from YNB –ura –leu to 5-FOA –leu

Construct	Construct	Number of (in)viable clones / total number of clones
FUS$^{1\text{-}163}$12E + Gal4^{G4} shuffle	viable	6 out of 6
Altered valence 2	viable	5 out of 6 (in total 5 out of 6, see also slide 23)
Rap1$^{231\text{-}361}$ + Gal4 G4	viable	5 out of 5
LS-8	viable	3 out of 3

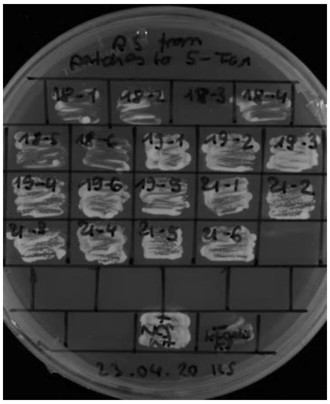

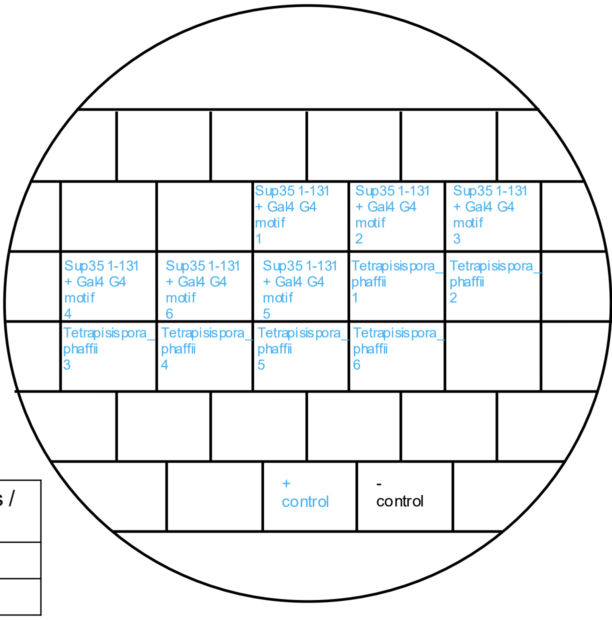

Restreak from YNB –ura –leu to 5-FOA –leu

Construct	Result regarding viability on 5-FOA	Number of (in)viable clones / total number of clones
Sup35$^{1-131}$ + Gal4^{G4}	viable	6 out of 6
T. phaffii	viable	6 out of 6

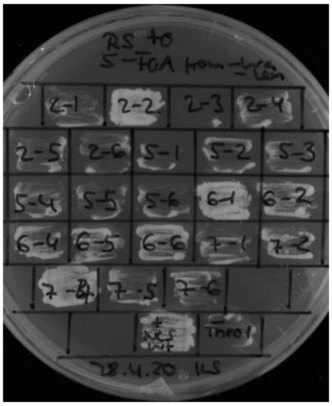

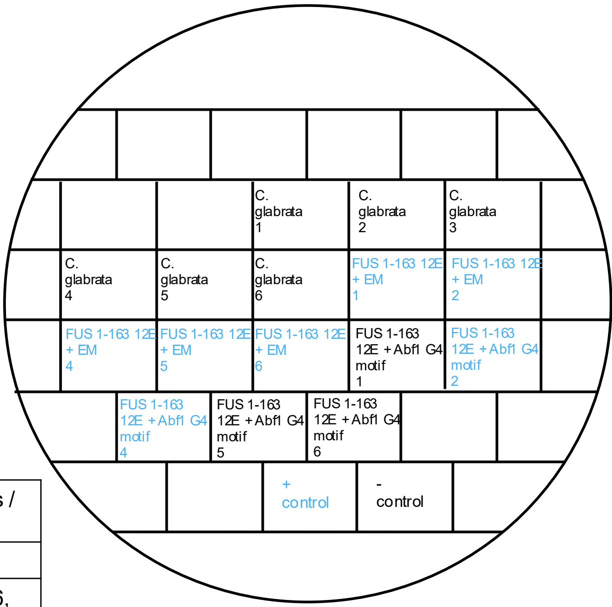

Restreak from YNB –ura –leu to 5-FOA –leu

Construct	Result regarding viability on 5-FOA	Number of (in)viable clones / total number of clones
C. glabrata	inviable	6 out of 6
FUS$^{1-163}$12E + EM	viable	5 out of 5 (in total 6 out of 6, see also slides 12, 23, 24)
FUS$^{1-163}$12E + Abf1^{G4}	viable	2 out of 5 (in total 2 out of 2, technical replicate, see also slide 12)

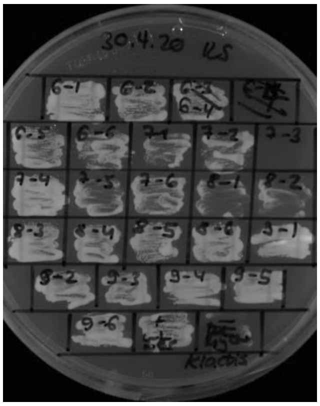

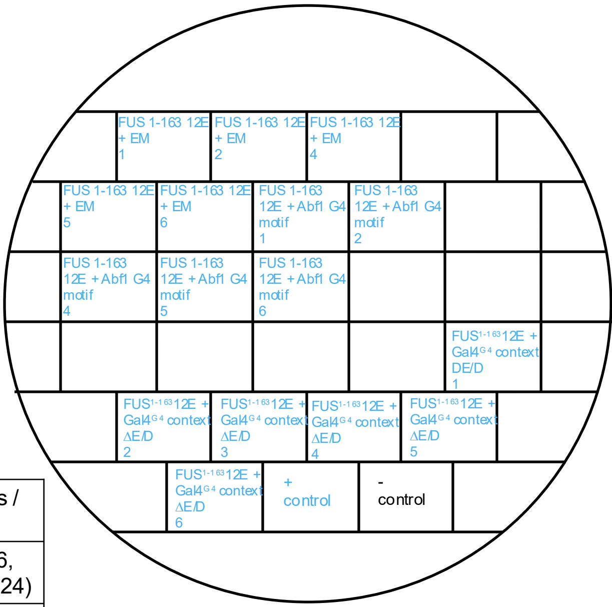

Restreak from YNB –ura –leu to 5-FOA –leu

Construct	Construct	Number of (in)viable clones / total number of clones
FUS$^{1\text{-}163}$12E + EM	viable	5 out of 5 (in total 6 out of 6, see also slides 11, 23 and 24)
FUS$^{1\text{-}163}$12E + Abf1^{G4}	viable	5 out of 5 (in total 2out of 2, technical replicate, see also slide 11)
FUS$^{1\text{-}163}$12E + Gal4^{G4} context ΔE/D	viable	6 out of 6

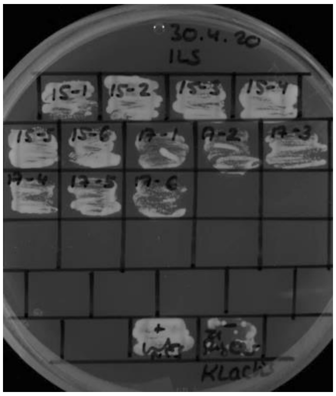

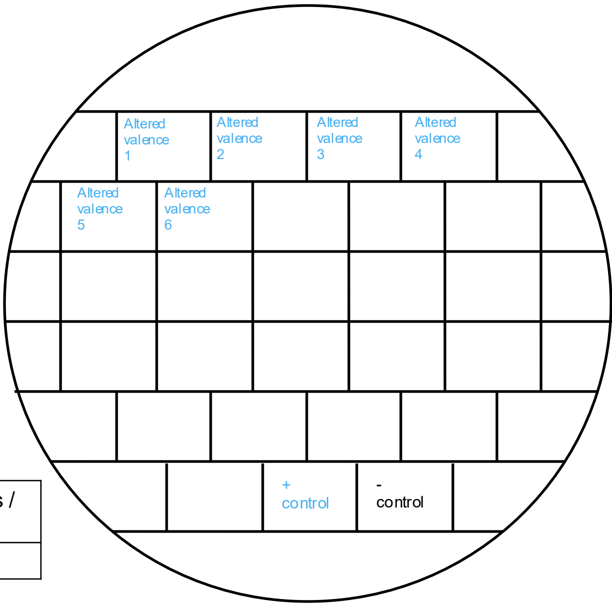

Restreak from YNB –ura –leu to 5-FOA –leu

Construct	Result regarding viability on 5-FOA	Number of (in)viable clones / total number of clones
Altered valence 1	viable	6 out of 6

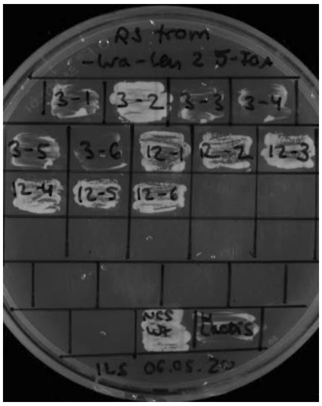

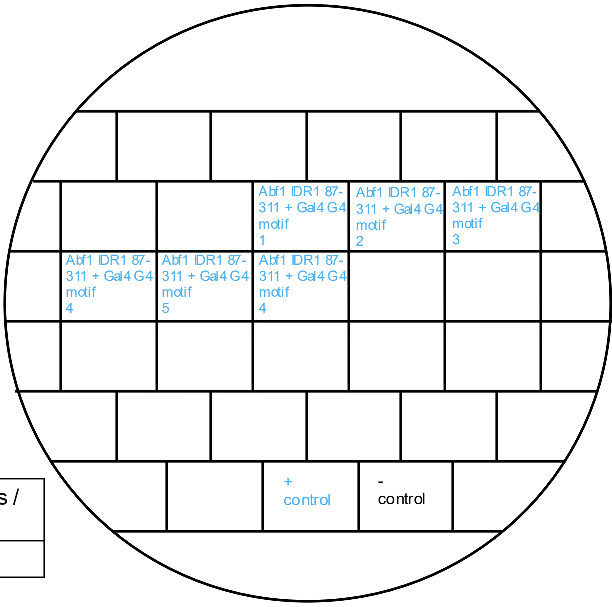

Restreak from YNB –ura –leu to 5-FOA –leu

Construct	Construct	Number of (in)viable clones / total number of clones
Abf1 IDR1$^{87-311}$ + Gal4^{G4}	viable	6 out of 6

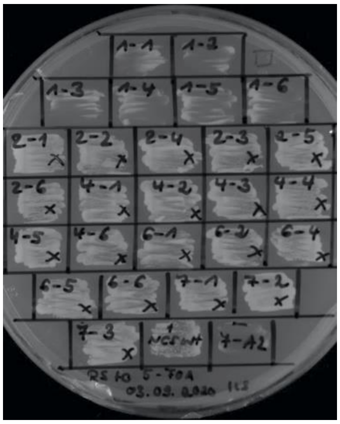

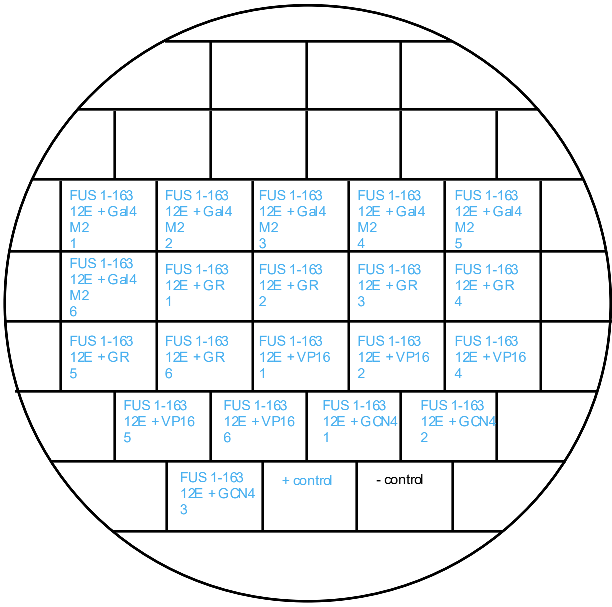

Restreak from YNB –ura –leu to 5-FOA –leu

Construct	Result regarding viability on 5-FOA	Number of (in)viable clones / total number of clones
FUS$^{1-163}$12E+ Gal4^{M2}	viable	6 out of 6
FUS$^{1-163}$12E+ GR	viable	6 out of 6
FUS$^{1-163}$12E+ VP16	viable	5 out of 5
FUS$^{1-163}$12E+ Gcn4	viable	3 out of 3 (in total 6 out of 6, see also slide 14)

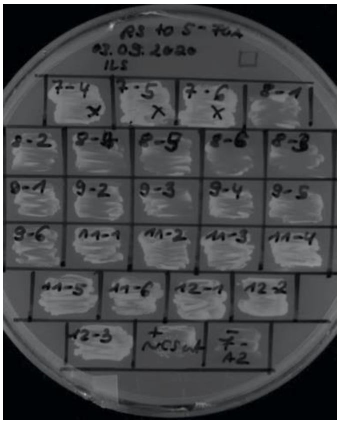

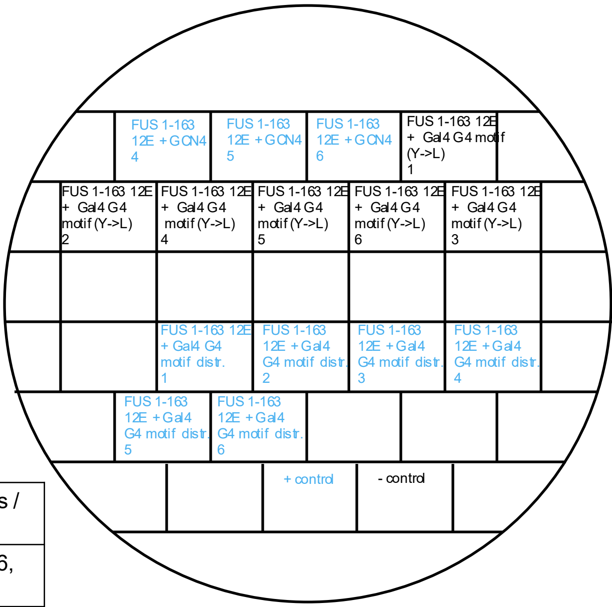

Restreak from YNB –ura –leu to 5-FOA –leu

Construct	Result regarding viability on 5-FOA	Number of (in)viable clones / total number of clones
FUS$^{1-163}$12E + Gcn4	viable	3 out of 3 (in total 6 out of 6, see also slide 13)
FUS$^{1-163}$12E + Gal4^{G4} Y→L in context	inviable	6 out of 6
FUS$^{1-163}$12E + Gal4^{G4} distr.	viable	6 out of 6

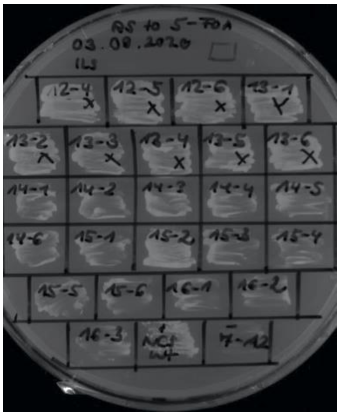

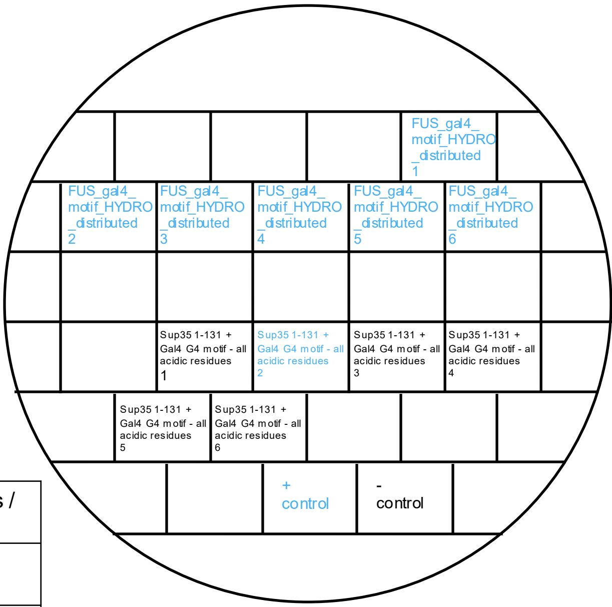

Restreak from YNB –ura –leu to 5-FOA –leu

Construct	Result regarding viability on 5-FOA	Number of (in)viable clones / total number of clones
FUS$^{1-163}$12E + Gal4^{G4} motif hydro distr.	viable	6 out of 6
Sup35$^{1-131}$ + Gal4^{G4} - all acidic residues	inviable	5 out of 6

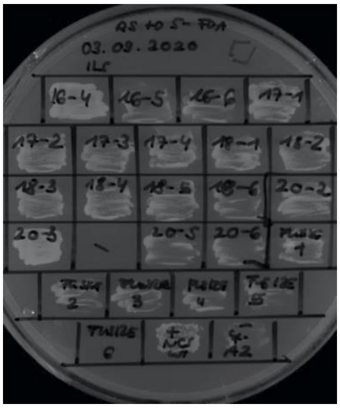

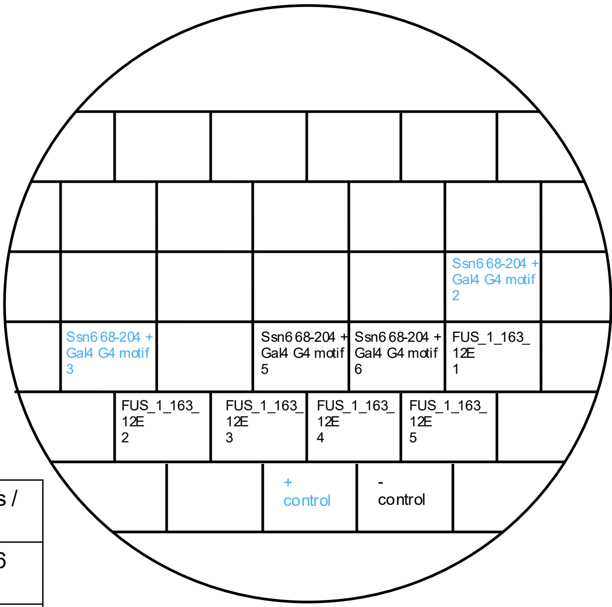

Restreak from YNB –ura –leu to 5-FOA –leu

Construct	Result regarding viability on 5-FOA	Number of (in)viable clones / total number of clones
Ssn6$^{68\text{-}204}$ + Gal4^{G4}	inviable	2 out of 4 (in total 4 out of 6 see plate below)
FUS$^{1\text{-}163}$12E	inviable	5 out of 5 (in total 8 out of 9, see also slides 7 and 8)

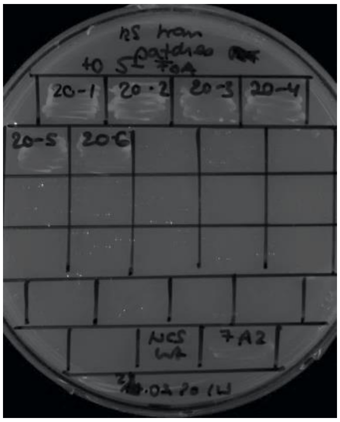

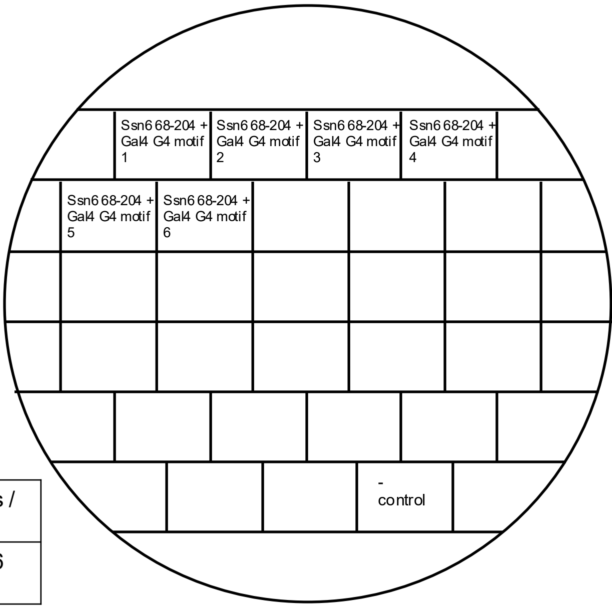

Restreak from YNB –ura –leu to 5-FOA –leu

Construct	Result regarding viability on 5-FOA	Number of (in)viable clones / total number of clones
Ssn6$^{68\text{-}204}$ + Gal4^{G4}	inviable	6 out of 6 (in total 4 out of 6 see plate above)

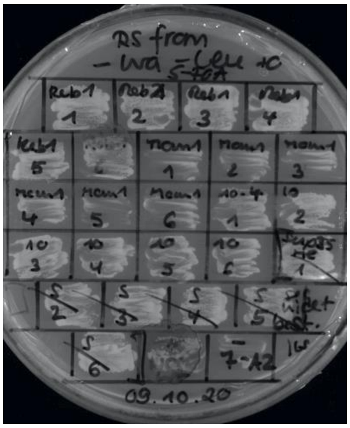

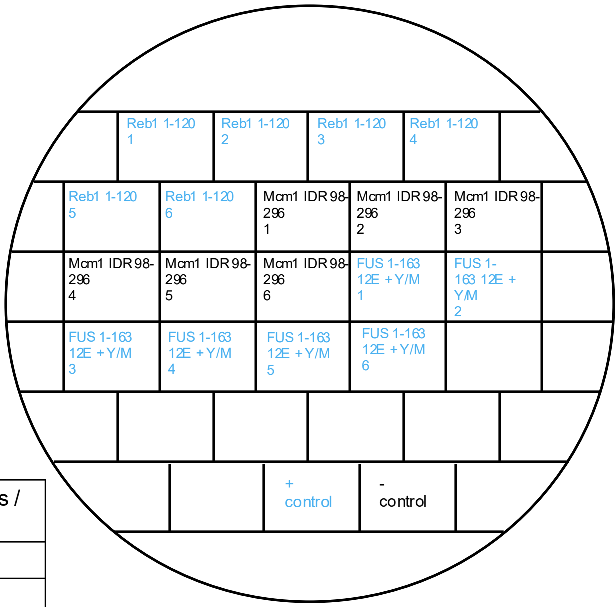

Restreak from YNB –ura –leu to 5-FOA –leu

Construct	Result regarding viability on 5-FOA	Number of (in)viable clones / total number of clones
Reb1$^{1-120(+PKEEEEGGL)}$	viable	6 out of 6
Mcm1$^{98-296}$	inviable	6 out of 6
FUS$^{1-163}$12E+ Y/M	viable	6 out of 6

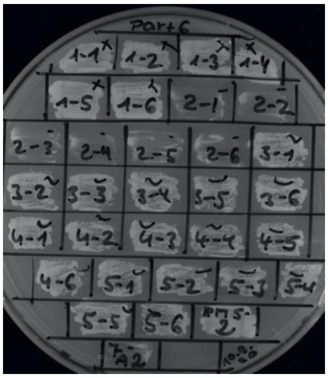

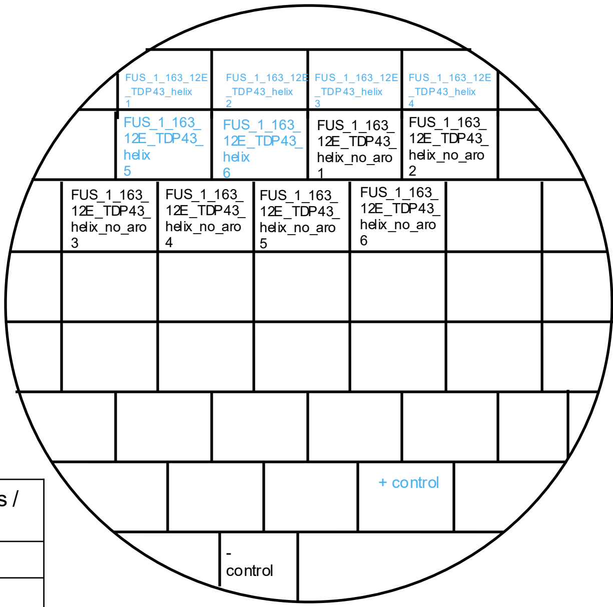

Restreak from YNB –ura –leu to 5-FOA –leu

Construct	Result regarding viability on 5-FOA	Number of (in)viable clones / total number of clones
FUS$^{1-163}$12E + TDP-43	viable	6 out of 6
FUS$^{1-163}$12E + TDP-43 Y→S in context	inviable	6 out of 6

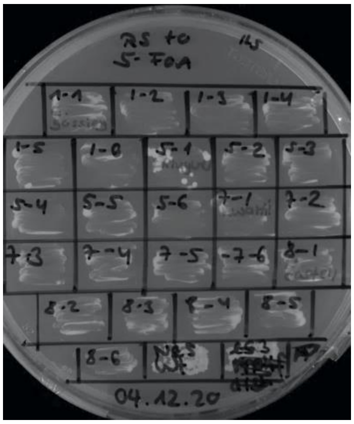

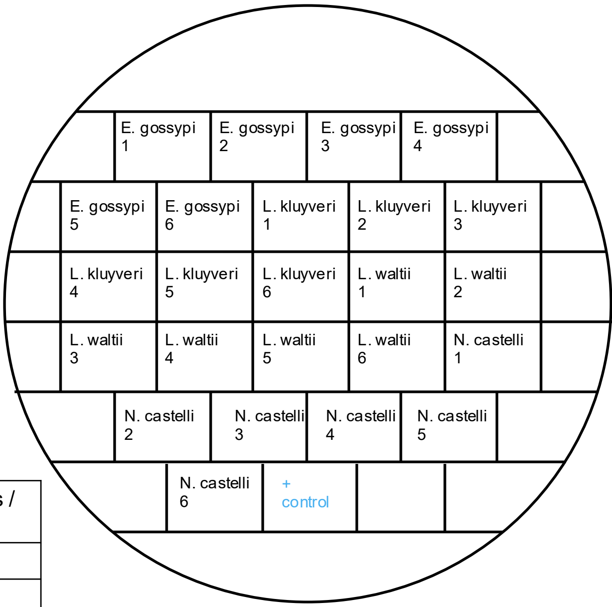

Restreak from YNB –ura –leu to 5-FOA –leu

Construct	Result regarding viability on 5-FOA	Number of (in)viable clones / total number of clones
E. gossypi	inviable	6 out of 6
L. kluyveri	inviable	6 out of 6
L. waltii	inviable	6 out of 6
N. castelli	inviable	6 out of 6

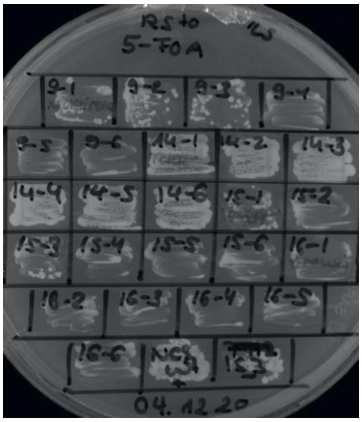

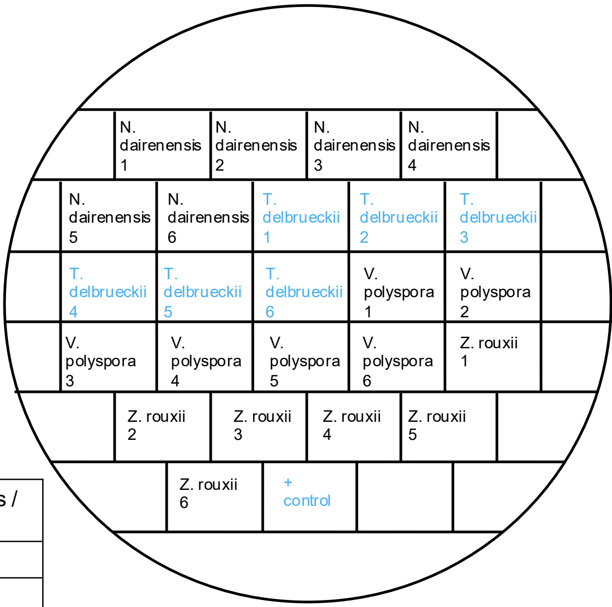

Restreak from YNB –ura –leu to 5-FOA –leu

Construct	Result regarding viability on 5-FOA	Number of (in)viable clones / total number of clones
N. dairenensis	inviable	6 out of 6
T. delbrueckii	viable	6 out of 6
V. polyspora	inviable	6 out of 6 (in total 14 out of 14, see also slide 37)
Z. rouxii	inviable	6 out of 6

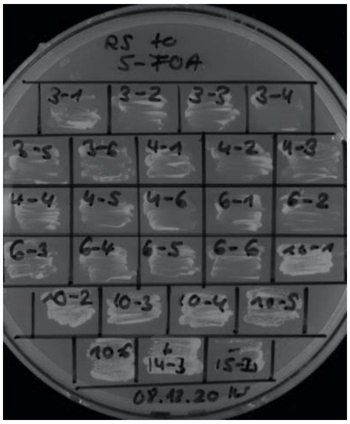

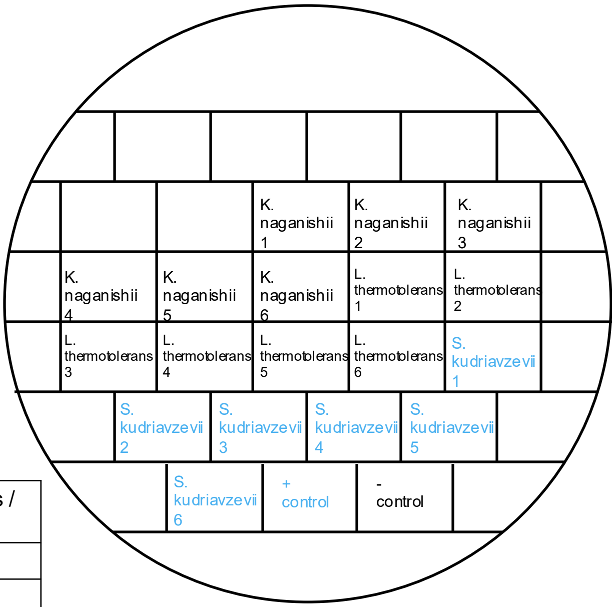

Restreak from YNB –ura –leu to 5-FOA –leu

Construct	Result regarding viability on 5-FOA	Number of (in)viable clones / total number of clones
K. naganishii	inviable	6 out of 6
L. thermotolerans	inviable	6 out of 6
S. kudriavzevii	viable	6 out of 6

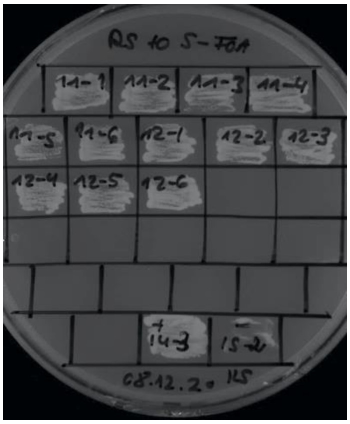

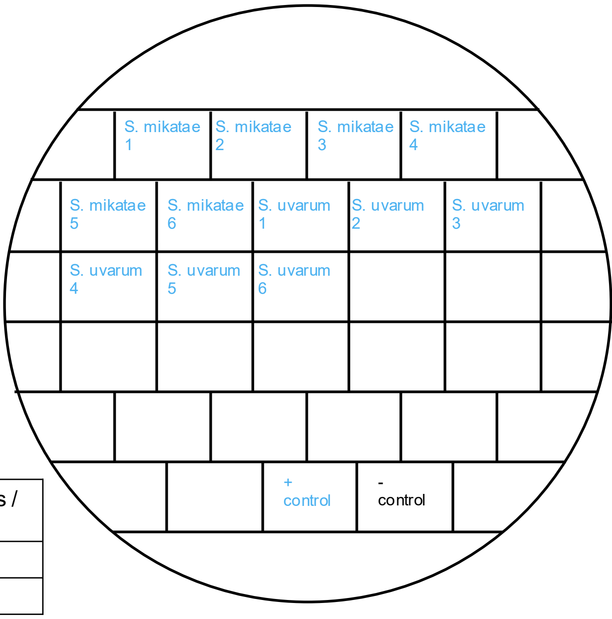

Restreak from YNB –ura –leu to 5-FOA –leu

Construct	Result regarding viability on 5-FOA	Number of (in)viable clones / total number of clones
S. mikatae	viable	6 out of 6
S. uvarum	viable	6 out of 6

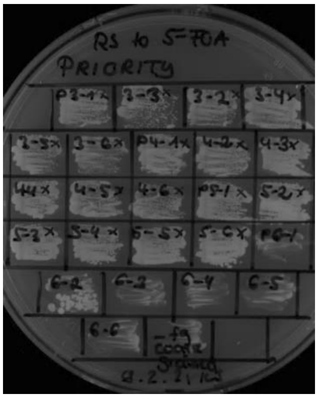

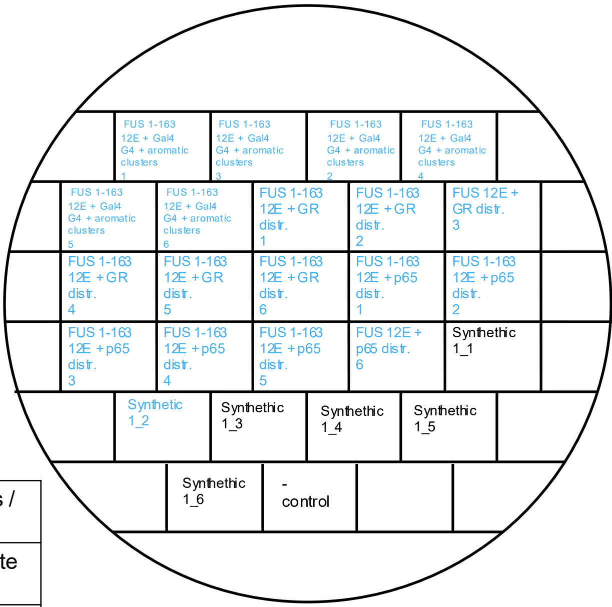

Restreak from YNB –ura –leu to 5-FOA –leu

Construct	Result regarding viability on 5-FOA	Number of (in)viable clones / total number of clones
FUS$^{1-163}$12E + Gal4^{G4} aromatic clusters	viable	6 out of 6 (technical replicate see slide 22)
FUS$^{1-163}$12E + GR distr.	viable	6 out of 6
FUS$^{1-163}$12E + p65 distr.	viable	6 out of 6
Synthetic 1	inviable	5 out of 6

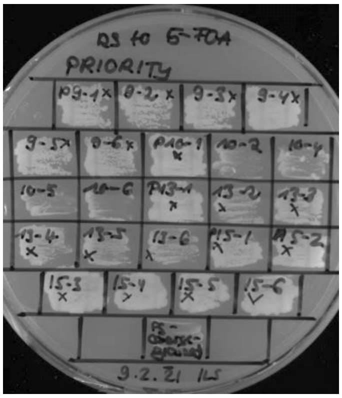

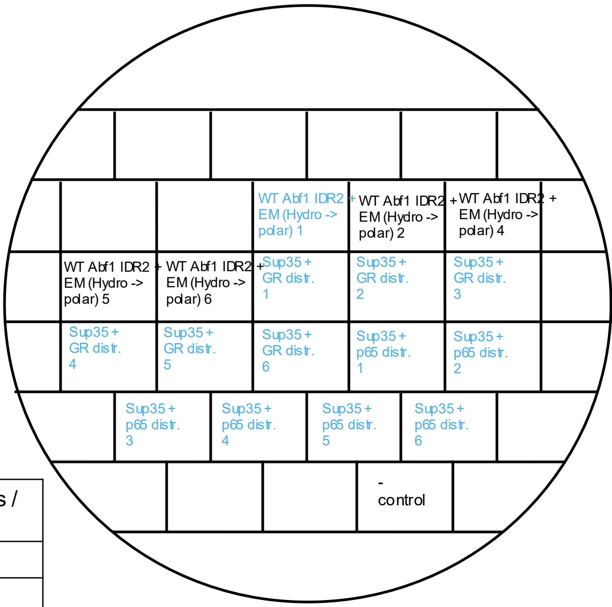

Restreak from YNB –ura –leu to 5-FOA –leu

Construct	Result regarding viability on 5-FOA	Number of (in)viable clones / total number of clones
IDR2 WT hydro→polar	inviable	4 out of 5
Sup35$^{1-131}$ + GR distr.	viable	6 out of 6
Sup35$^{1-131}$ + p65 distr.	viable	6 out of 6

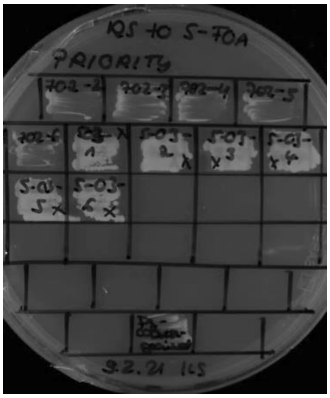

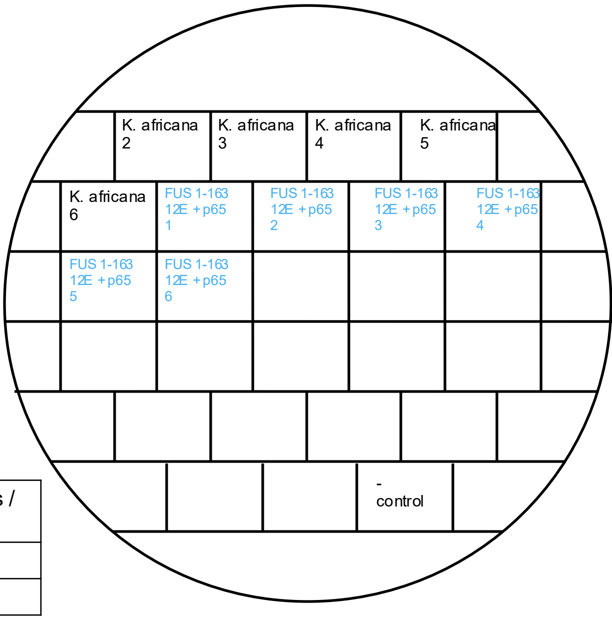

Restreak from YNB –ura –leu to 5-FOA –leu

Construct	Result regarding viability on 5-FOA	Number of (in)viable clones / total number of clones
K. africana	inviable	5 out of 5
FUS$^{1-163}$12E + p65	viable	6 out of 6

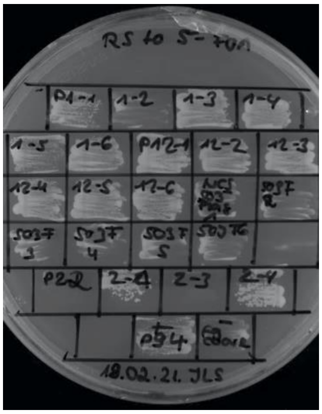

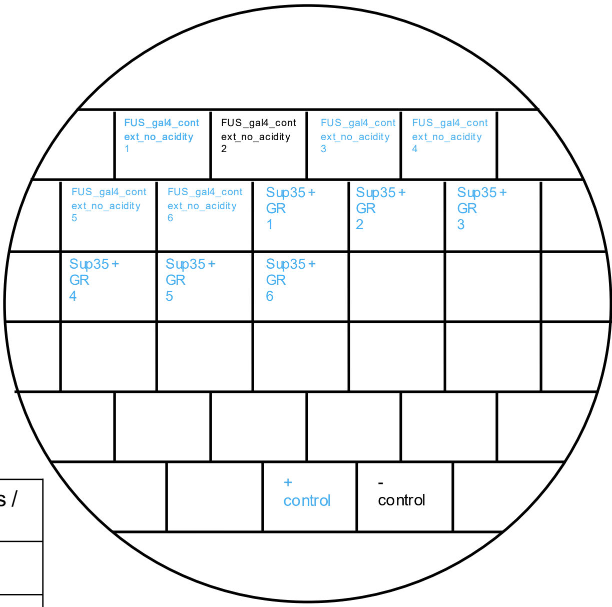

Restreak from YNB –ura –leu to 5-FOA –leu

Construct	Result regarding viability on 5-FOA	Number of (in)viable clones / total number of clones
FUS$^{1-163}$12E + Gal4^{G4} context ΔE/D	viable	5 out of 6
Sup35$^{1-131}$ + GR	viable	6 out of 6

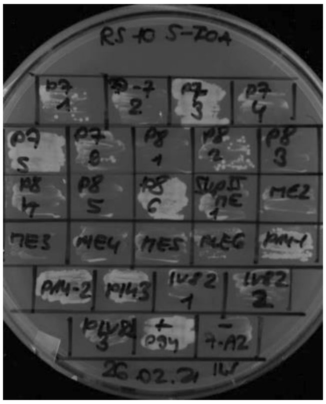

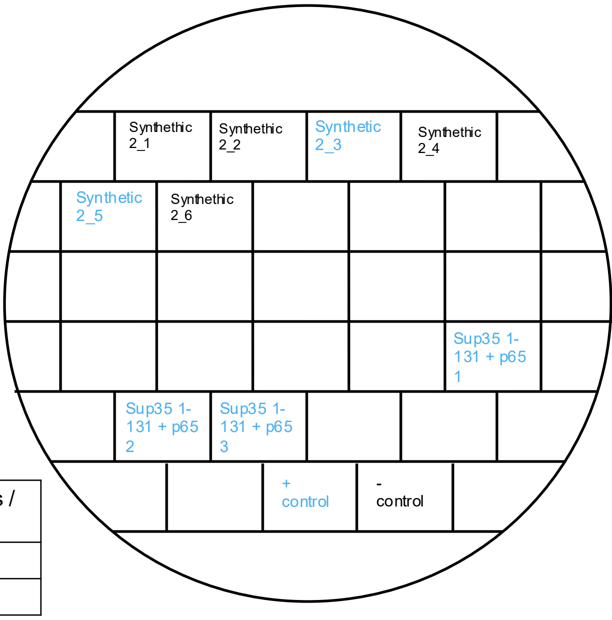

Restreak from YNB –ura –leu to 5-FOA –leu

Construct	Result regarding viability on 5-FOA	Number of (in)viable clones / total number of clones
Synthetic 2	inviable	4 out of 6
Sup35$^{1\text{-}131}$ + p65	viable	3 out of 3

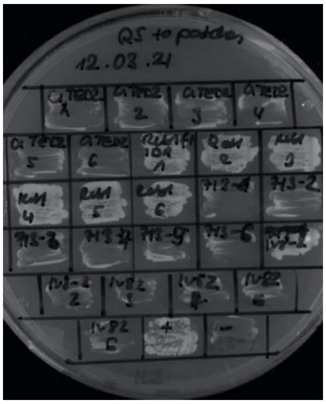

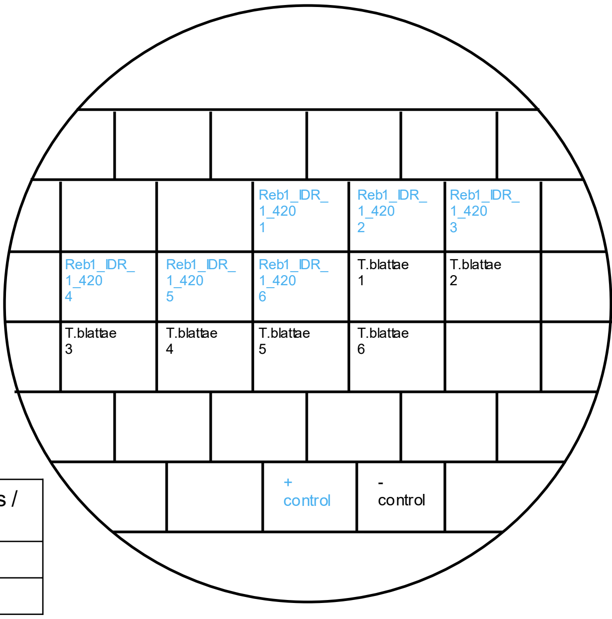

Restreak from YNB –ura –leu to 5-FOA –leu

Construct	Result regarding viability on 5-FOA	Number of (in)viable clones / total number of clones
Reb1$^{1\text{-}420}$	viable	6 out of 6
T. blattae	inviable	6 out of 6

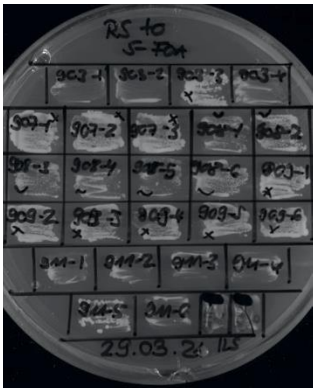

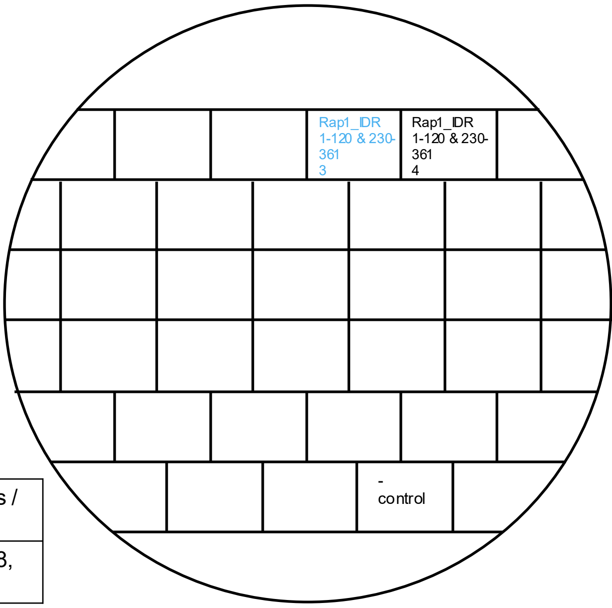

Restreak from YNB –ura –leu to 5-FOA –leu

Construct	Result regarding viability on 5-FOA	Number of (in)viable clones / total number of clones
Rap1$^{1-120\ \&\ 230-361}$	inviable	1 out of 2 (in total 7 out of 8, see also slide 40)

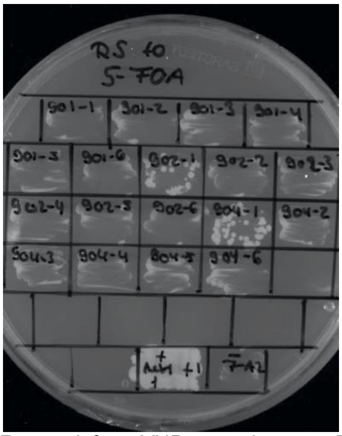

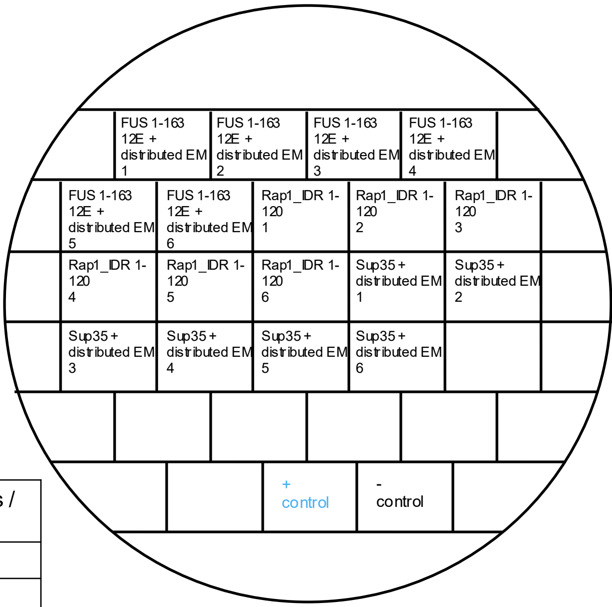

Restreak from YNB –ura –leu to 5-FOA –leu

Construct	Result regarding viability on 5-FOA	Number of (in)viable clones / total number of clones
FUS$^{1-163}$12E + EM distr.	inviable	6 out of 6
Rap1$^{1-120}$	inviable	6 out of 6
Sup35$^{1-131}$ + EM distr.	inviable	6 out of 6

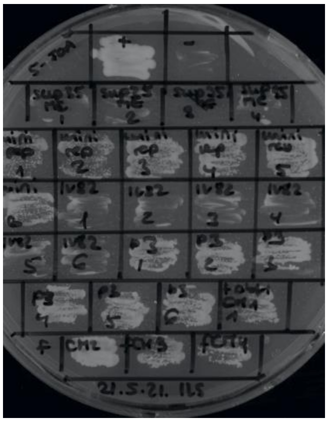

Restreak from YNB –ura –leu to 5-FOA –leu

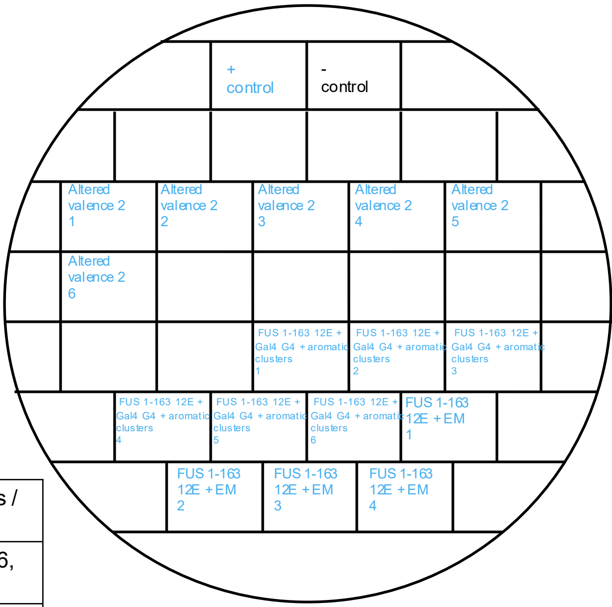

Construct	Result regarding viability on 5-FOA	Number of (in)viable clones / total number of clones
Altered valence 2	viable	6 out of 6 (in total 5 out of 6, see also slide 10)
FUS$^{1-163}$12E + Gal4^{G4} aromatic clusters	viable	6 out of 6 (technical replicate see slide 19)
FUS$^{1-163}$12E + EM	viable	4 out of 4 (in total 6 out of 6, see also slides 11, 12, 24)

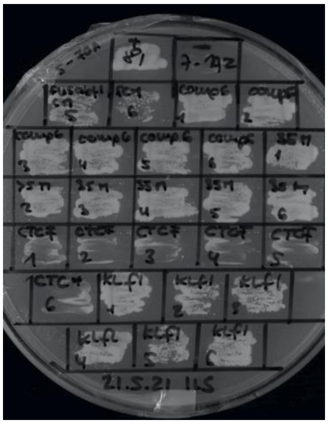

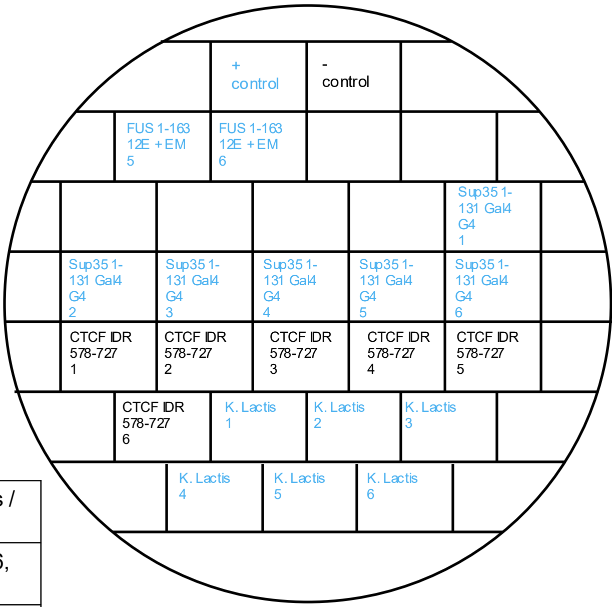

Restreak from YNB –ura –leu to 5-FOA –leu

Construct	Result regarding viability on 5-FOA	Number of (in)viable clones / total number of clones
FUS$^{1\text{-}163}$12E + EM	viable	2 out of 2 (in total 6 out of 6, see also slides 11, 12, 23)
Sup35$^{1\text{-}131}$ + Gal4^{G4}	viable	6 out of 6
CTCF$^{578\text{-}727}$	inviable	6 out of 6 (technical repliate, see slide 25)
K. Lactis	viable	6 out of 6

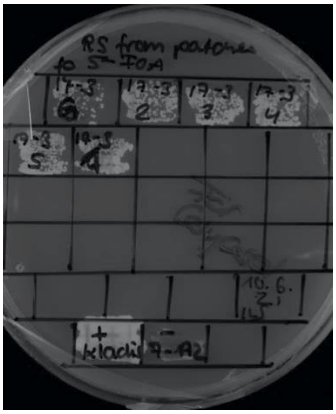

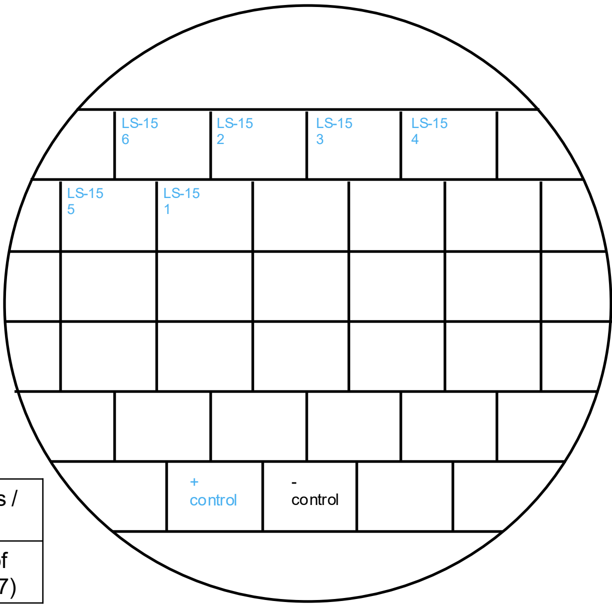

Restreak from YNB –ura –leu to 5-FOA –leu

Construct	Result regarding viability on 5-FOA	Number of (in)viable clones / total number of clones
LS-15	viable	6 out of 6 (in total 14 out of 14 clones, see also slide 37)

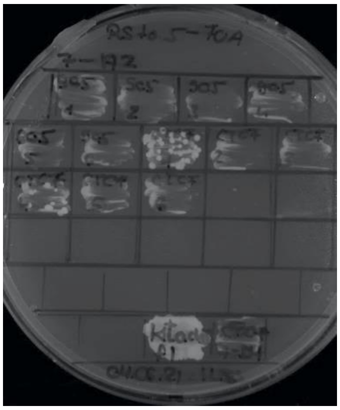

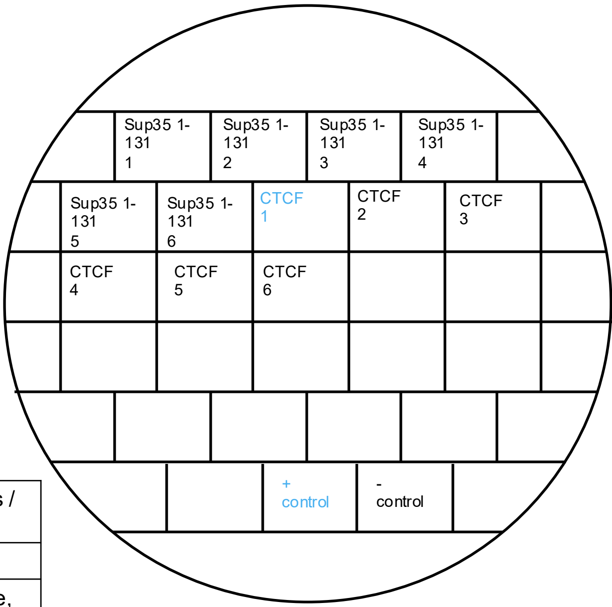

Restreak from YNB –ura –leu to 5-FOA –leu

Construct	Result regarding viability on 5-FOA	Number of (in)viable clones / total number of clones
Sup35$^{1-131}$	inviable	6 out of 6
CTCF$^{578-727}$	inviable	5 out of 6 (technical repliate, see slide 24)

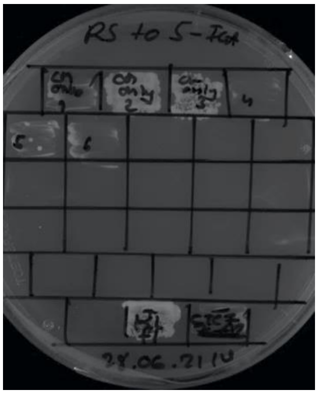

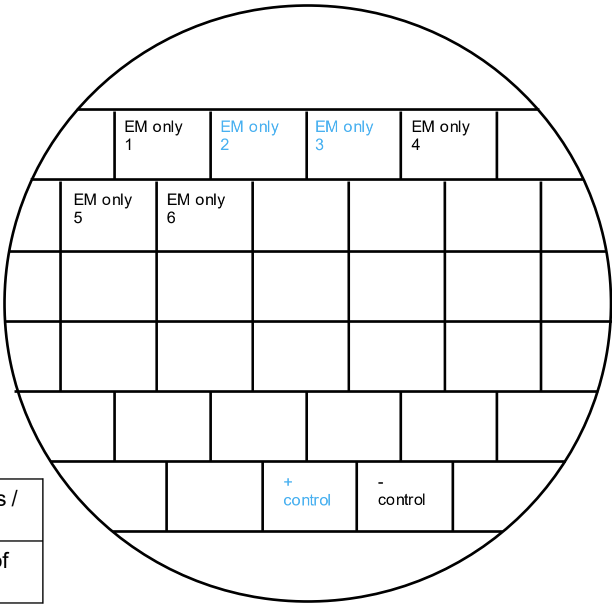

Restreak from YNB –ura –leu to 5-FOA –leu

Construct	Result regarding viability on 5-FOA	Number of (in)viable clones / total number of clones
EM only	inviable	4 out of 6 (in total: 10 out of 12, see also slide 26)

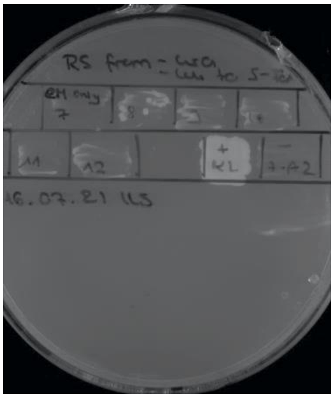

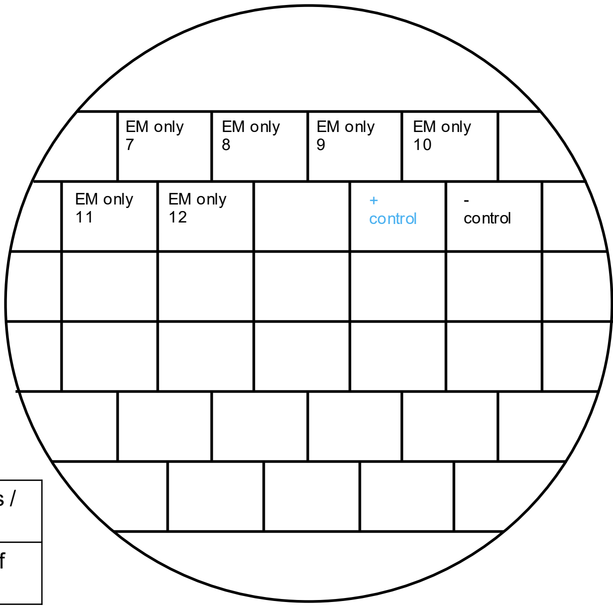

Restreak from YNB –ura –leu to 5-FOA –leu

Construct	Result regarding viability on 5-FOA	Number of (in)viable clones / total number of clones
EM only	Inviable	6 out of 6 (in total: 10 out of 12, see also slide 25)

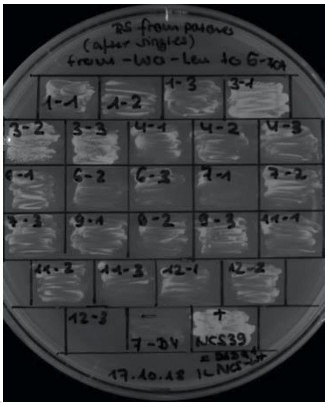

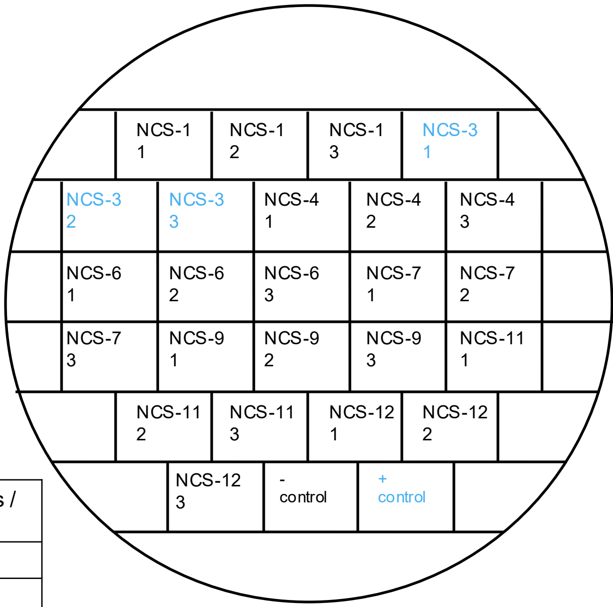

Restreak from YNB –ura –leu to 5-FOA –leu

Construct	Result regarding viability on 5-FOA	Number of (in)viable clones / total number of clones
NCS-1	inviable	3 out of 3
NCS-3	viable	3 out of 3
NCS-4	inviable	3 out of 3
NCS-6	inviable	3 out of 3
NCS-7	inviable	3 out of 3
NCS-9	inviable	3 out of 3
NCS-11	inviable	3 out of 3
NCS-12	inviable	3 out of 3

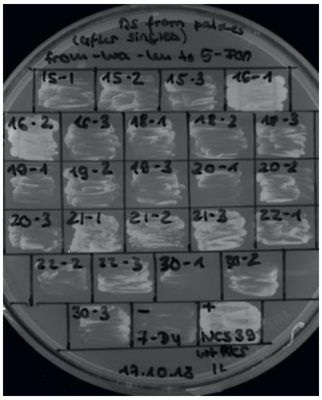

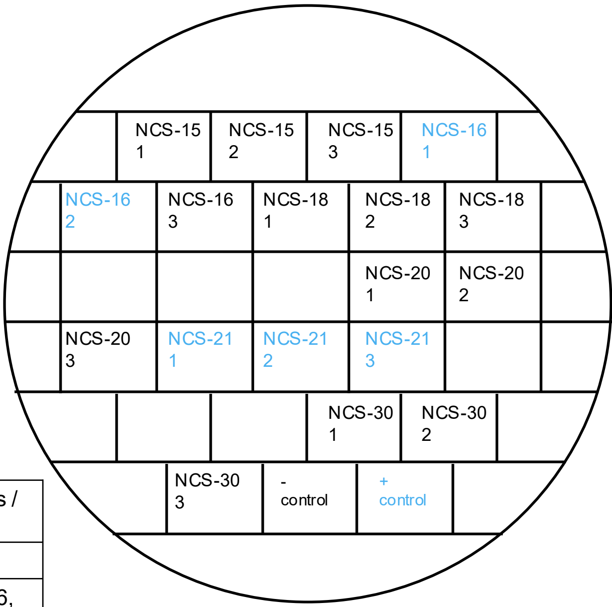

Restreak from YNB –ura –leu to 5-FOA –leu

Construct	Result regarding viability on 5-FOA	Number of (in)viable clones / total number of clones
NCS-15	inviable	3 out of 3
NCS-16	inviable	1 out of 3 (in total 4 out of 6, see slide 28)
NCS-18	inviable	3 out of 3
NCS-20	inviable	3 out of 3
NCS-21	viable	3 out of 3
NCS-30	inviable	3 out of 3

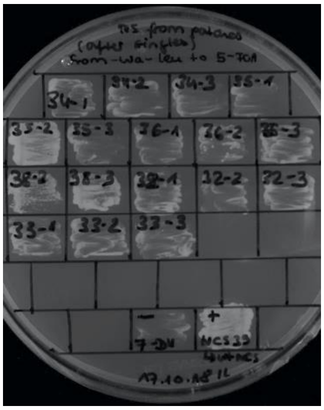

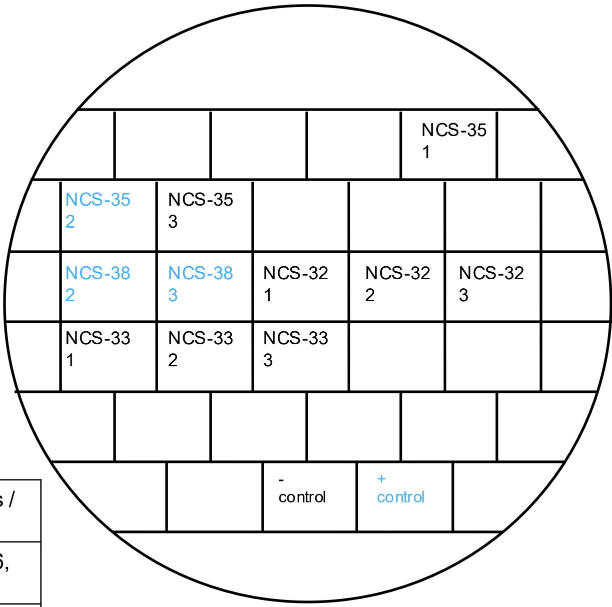

Restreak from YNB –ura –leu to 5-FOA –leu

Construct	Result regarding viability on 5-FOA	Number of (in)viable clones / total number of clones
NCS-35	inviable	2 out of 3 (in total 5 out of 6, see also slide 27)
NCS-38	viable	2 out of 2 (in total 5 out of 6, see also slide 27)
NCS-32	inviable	3 out of 3
NCS-33	inviable	3 out of 3

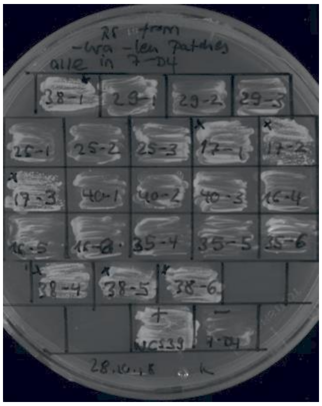

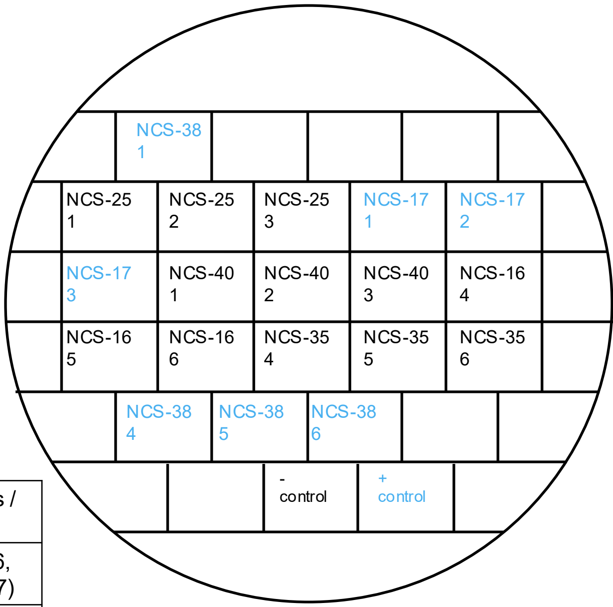

Restreak from YNB –ura –leu to 5-FOA –leu

Construct	Result regarding viability on 5-FOA	Number of (in)viable clones / total number of clones
NCS-38	viable	4 out of 4 (in total 5 out of 6, see also below and slide 27)
NCS-25	inviable	3 out of 3
NCS-17	viable	3 out of 3
NCS-40	inviable	3 out of 3
NCS-16	inviable	3 out of 3 (in total 4 out of 6, see below and slide 27)
NCS-35	inviable	3 out of 3 (in total 5 out of 6, see also below and slide 27)

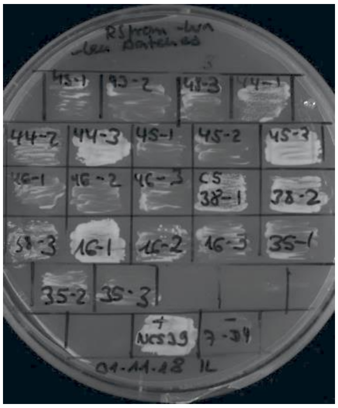

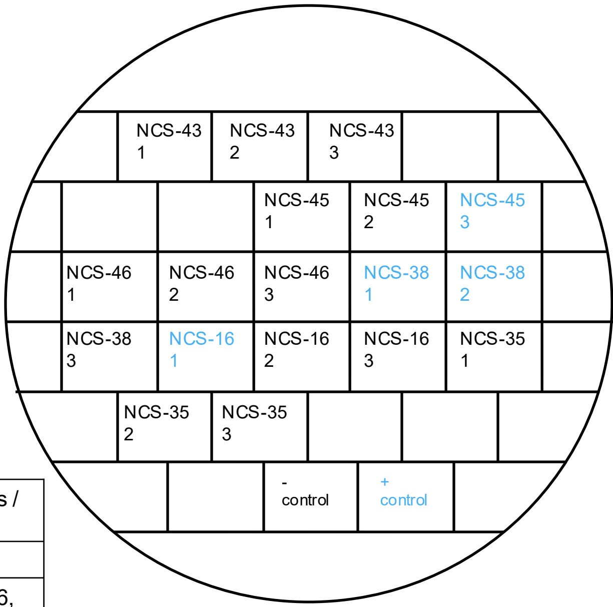

Restreak from YNB –ura –leu to 5-FOA –leu

Construct	Result regarding viability on 5-FOA	Number of (in)viable clones / total number of clones
NCS-43	inviable	3 out of 3
NCS-45	inviable	2 out of 3 (in total 5 out of 6, see also slide 30)
NCS-46	inviable	3 out of 3
NCS-38	viable	2 out of 3 (in total 5 out of 6, see also above and slide 27)
NCS-16	inviable	2 out of 3 (in total 4 out of 6, see above and slide 27)
NCS-35	inviable	3 out of 3 (in total 5 out of 6, see also above and slide 27)

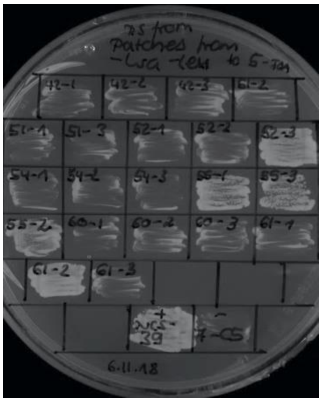

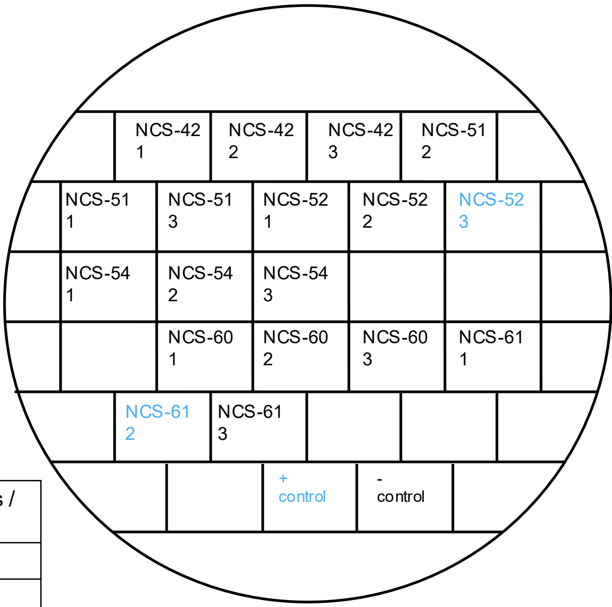

Restreak from YNB –ura –leu to 5-FOA –leu

Construct	Result regarding viability on 5-FOA	Number of (in)viable clones / total number of clones
NCS-42	inviable	3 out of 3
NCS-51	inviable	3 out of 3
NCS-52	inviable	2 out of 3 (in total 5 out of 6 see also below)
NCS-54	inviable	3 out of 3
NCS-60	inviable	3 out of 3
NCS-61	inviable	2 out of 3 (in total 5 out of 6 see also below)

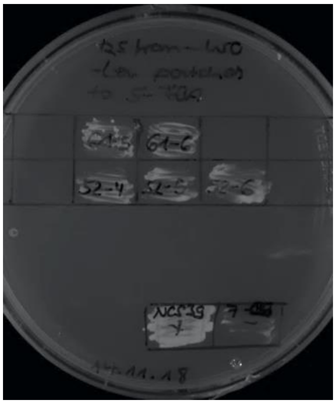

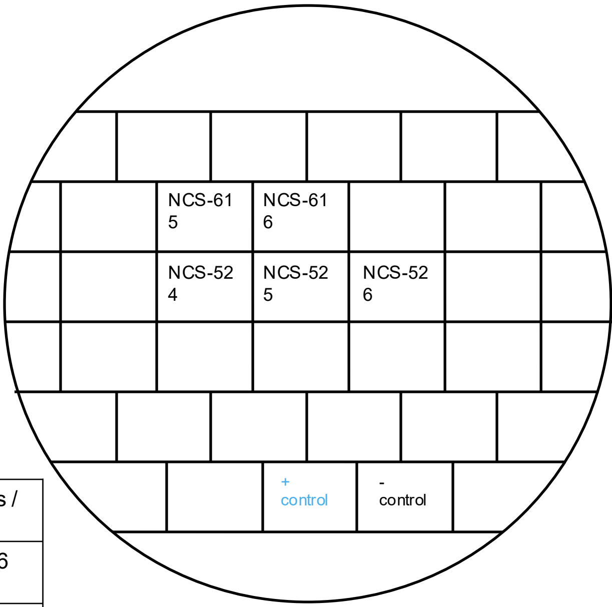

Restreak from YNB –ura –leu to 5-FOA –leu

Construct	Result regarding viability on 5-FOA	Number of (in)viable clones / total number of clones
NCS-61	inviable	2 out of 2 (in total 5 out of 6 see also above)
NCS-52	inviable	3 out of 3 (in total 5 out of 6 see also above)

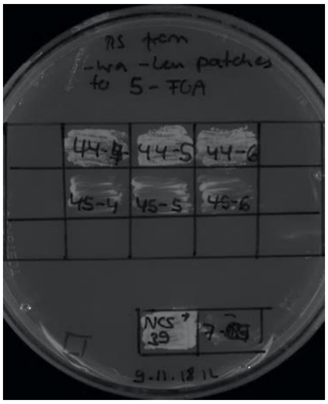

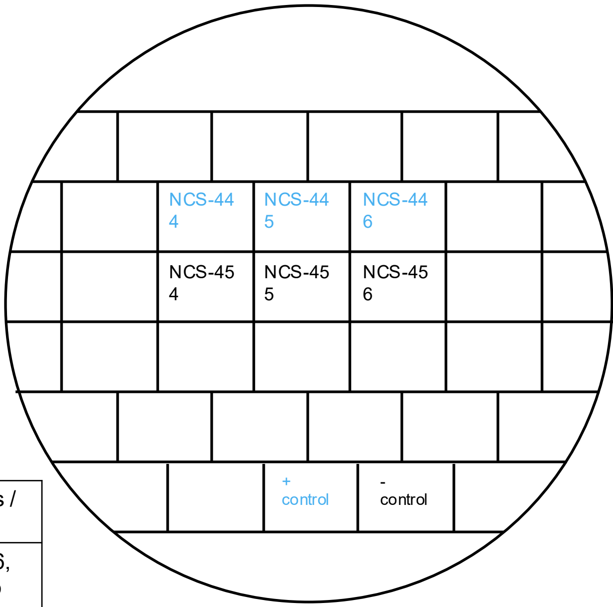

Restreak from YNB –ura –leu to 5-FOA –leu

Construct	Result regarding viability on 5-FOA	Number of (in)viable clones / total number of clones
NCS-44	viable	3 out of 3 (in total 6 out of 6, technical replicate see also below)
NCS-45	inviable	3 out of 3 (in total 5 out of 6, see also slide 28)

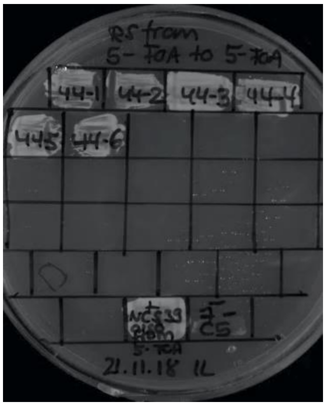

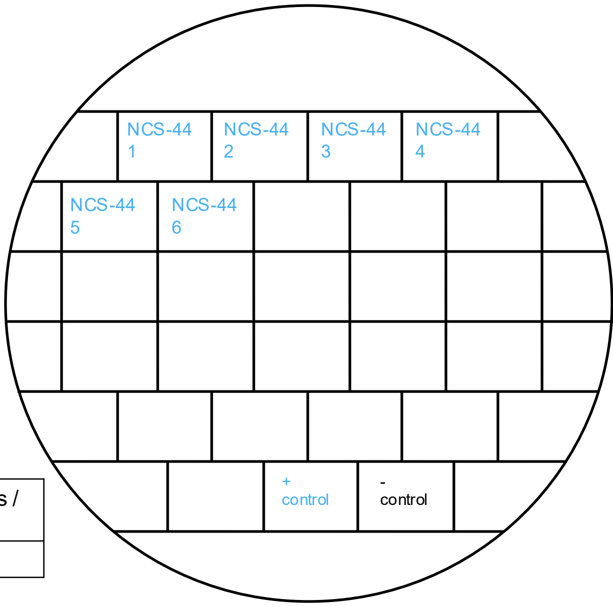

Restreak from 5-FOA – leu to 5-FOA –leu

Construct	Result regarding viability on 5-FOA	Number of (in)viable clones / total number of clones
NCS-44	viable	6 out of 6

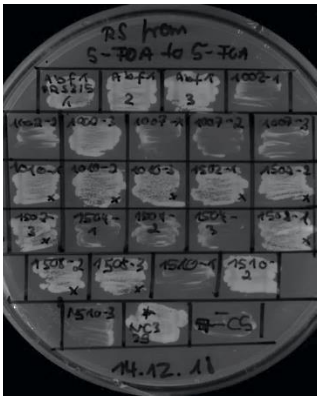

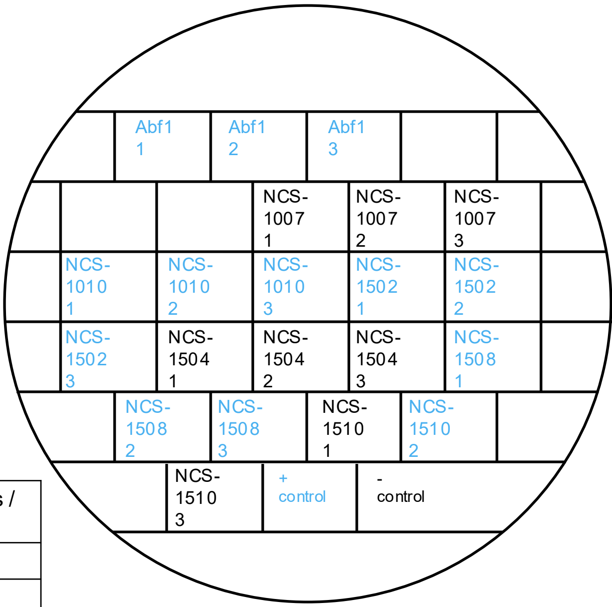

Restreak from YNB –ura –leu to 5-FOA –leu

Construct	Result regarding viability on 5-FOA	Number of (in)viable clones / total number of clones
WT Abf1	viable	3 out of 3
NCS-1007	inviable	3 out of 3
NCS-1010	viable	3 out of 3
NCS-1502	viable	3 out of 3
NCS-1504	inviable	3 out of 3
NCS-1508	viable	3 out of 3
NCS-1510	inviable	2 out of 3 (in total 12 out of 15, see also slides 32 and 33)

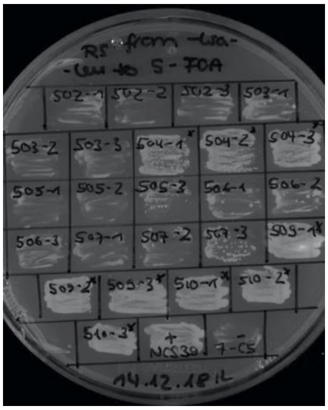

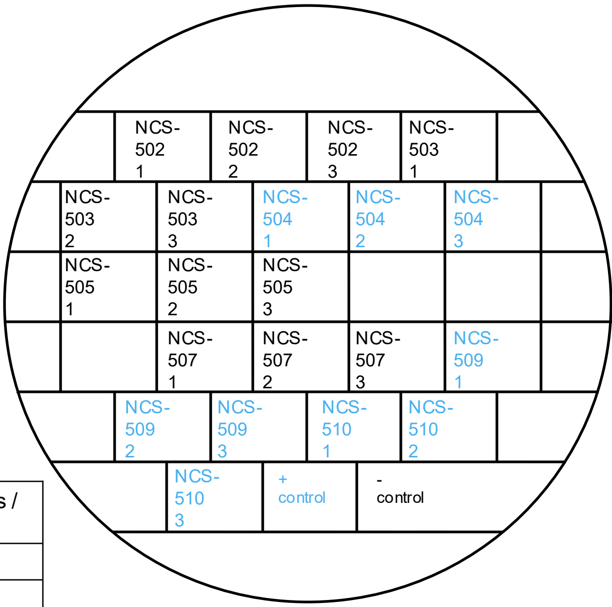

Restreak from YNB –ura –leu to 5-FOA –leu

Construct	Result regarding viability on 5-FOA	Number of (in)viable clones / total number of clones
NCS-502	inviable	3 out of 3
NCS-503	inviable	3 out of 3
NCS-504	viable	3 out of 3
NCS-505	inviable	3 out of 3
NCS-507	inviable	3 out of 3
NCS-509	viable	3 out of 3
NCS-510	viable	3 out of 3

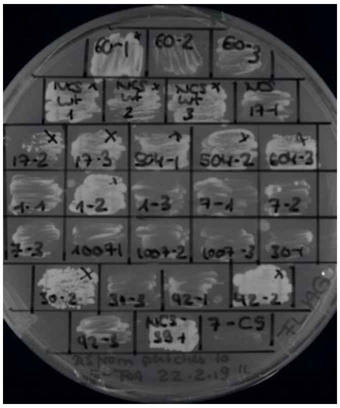

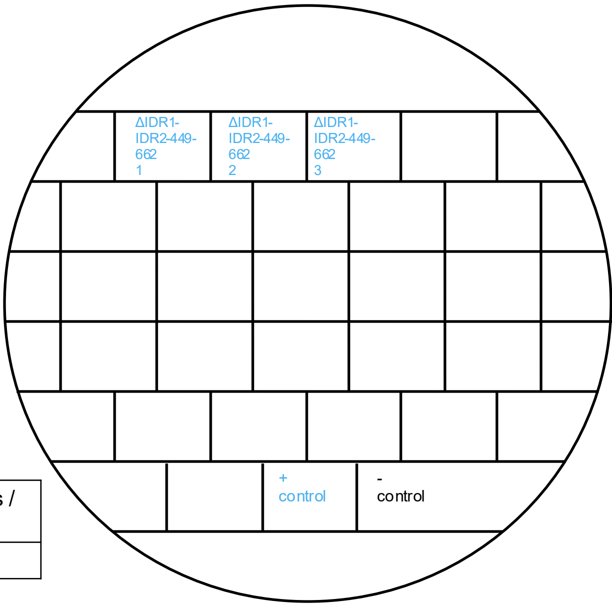

Restreak from YNB –ura –leu to 5-FOA –leu

Construct	Result regarding viability on 5-FOA	Number of (in)viable clones / total number of clones
ΔIDR1 & IDR2$^{449\text{-}662}$	viable	3 out of 3

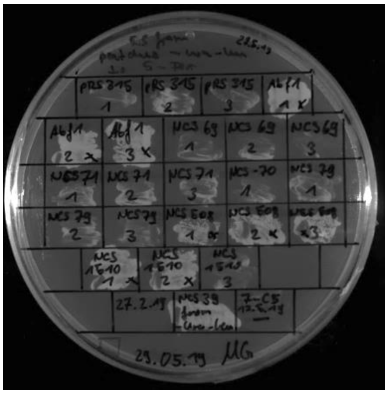

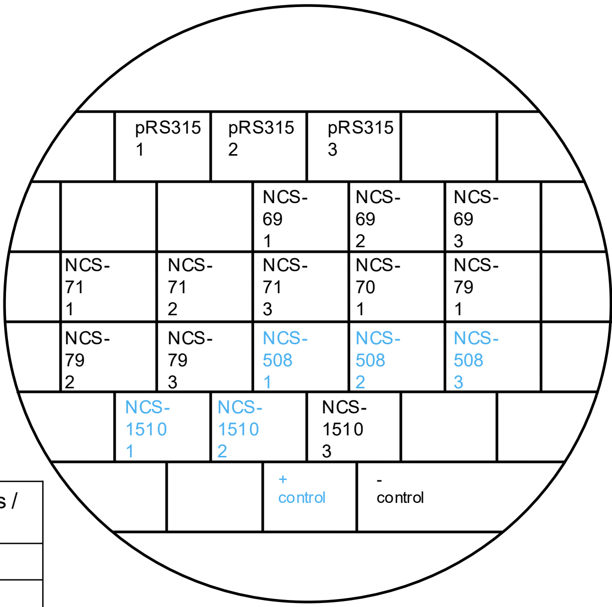

Restreak from YNB –ura –leu to 5-FOA –leu

Construct	Result regarding viability on 5-FOA	Number of (in)viable clones / total number of clones
pRS315 empty	inviable	3 out of 3
NCS-69	inviable	3 out of 3
NCS-71	inviable	3 out of 3
NCS-70	inviable	1 out of 1 (in total 3 out of 3, see slide 33)
NCS-79	inviable	3 out of 3
NCS-508	viable	3 out of 3
NCS-1510	inviable	1 out of 3 (in total: 12 out of 15, see also slides 31 and 33)

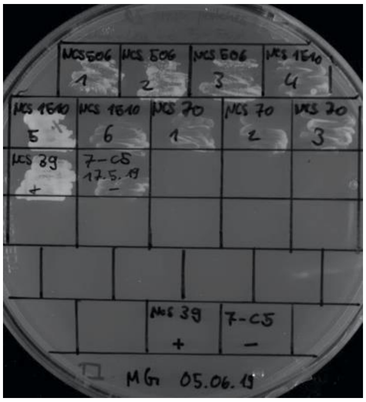

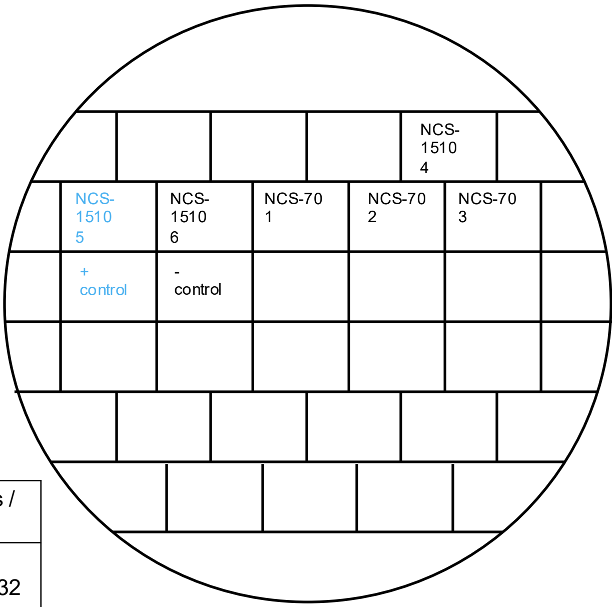

Restreak from YNB –ura –leu to 5-FOA –leu

Construct	Result regarding viability on 5-FOA	Number of (in)viable clones / total number of clones
NCS-1510	inviable	2 out of 3 (in total 12 out of 15, see also slides 31 and 32 and below)
NCS-70	inviable	3 out of 3 (in total 3 out of 3, see slide 32)

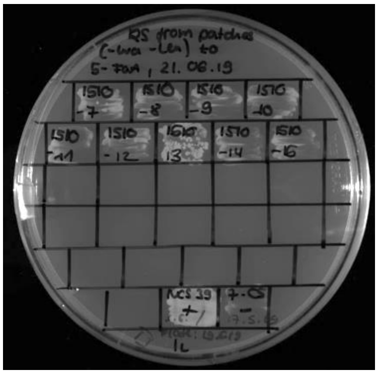

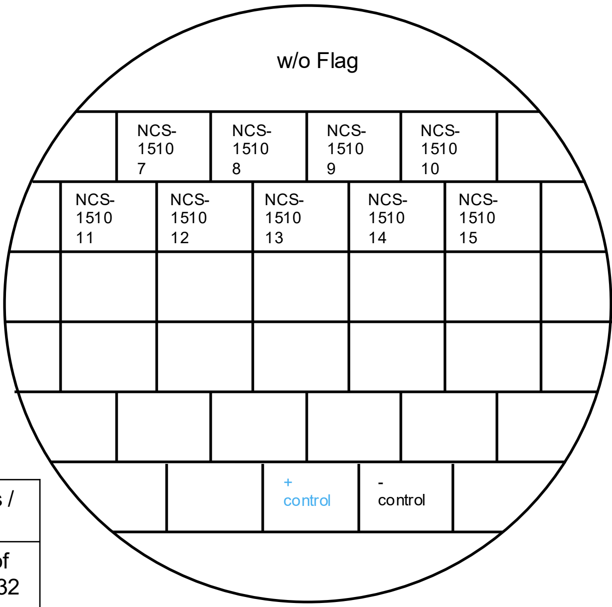

Restreak from YNB –ura –leu to 5-FOA –leu

Construct	Result regarding viability on 5-FOA	Number of (in)viable clones / total number of clones
NCS-1510	inviable	9 out of 9 (in total: 12 out of 15, see also slides 31 and 32 and above)

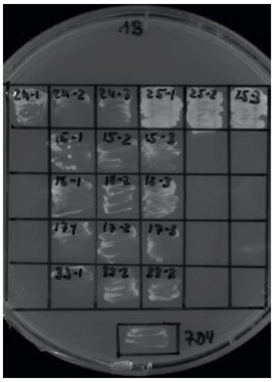

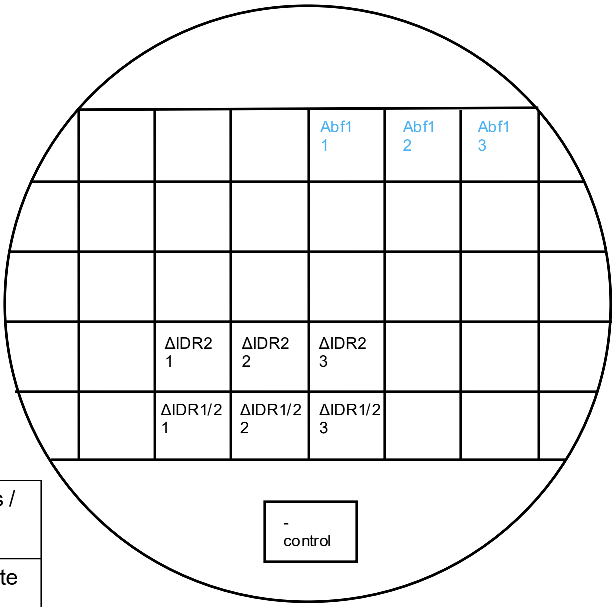

Restreak from YNB –ura –leu to 5-FOA –leu

Construct	Result regarding viability on 5-FOA	Number of (in)viable clones / total number of clones
Abf1 (NLS)	viable	3 out of 3 (technical replicate see below)
ΔIDR2	inviable	3 out of 3
ΔIDR1/2	inviable	3 out of 3

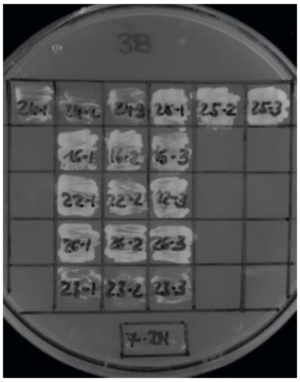

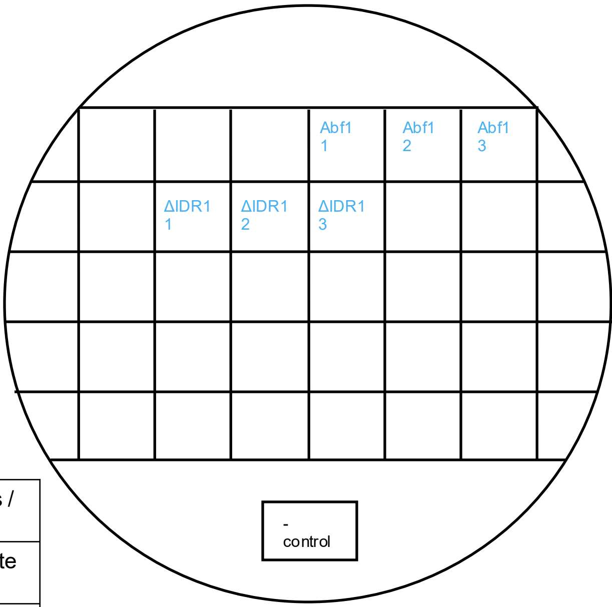

Restreak from YNB –ura –leu to 5-FOA –leu

Construct	Result regarding viability on 5-FOA	Number of (in)viable clones / total number of clones
Abf1 (NLS)	viable	3 out of 3 (technical replicate see above)
ΔIDR1	viable	3 out of 3

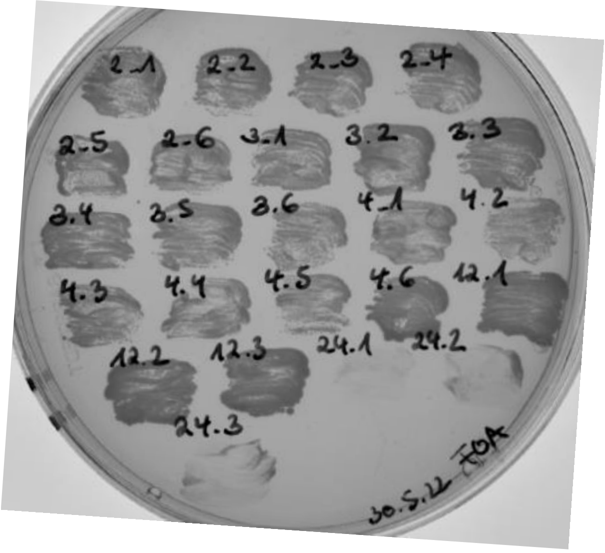

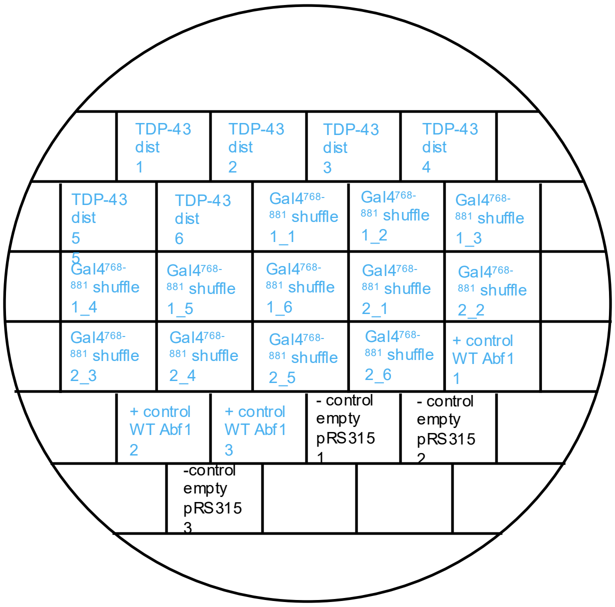

Restreak from YNB –ura –leu to 5-FOA –leu

Construct	Result regarding viability on 5-FOA	Number of (in)viable clones / total number of clones
TDP-43 distr.	viable	6 out of 6
Gal4$^{768-881}$ shuffle 1	viable	6 out of 6
Gal4$^{768-881}$ shuffle 2	viable	6 out of 6

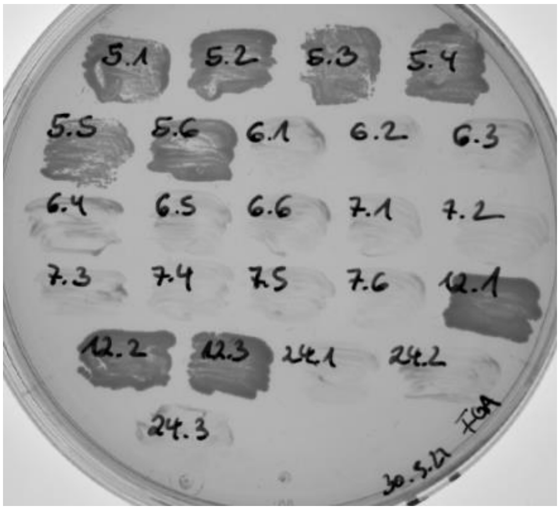

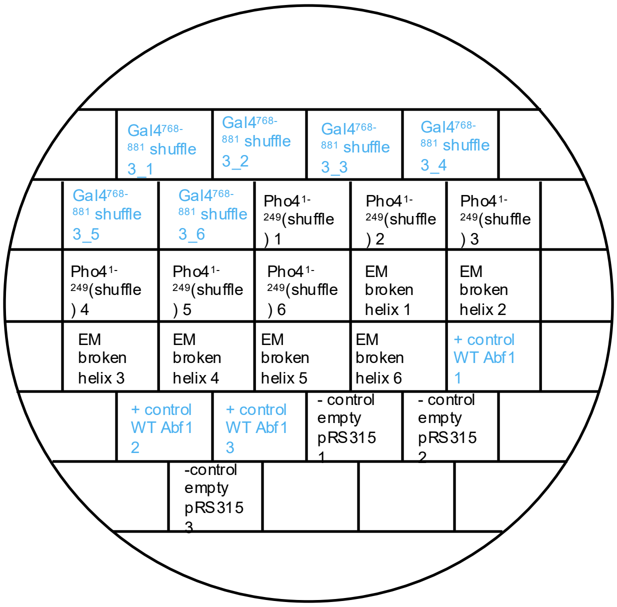

Restreak from YNB –ura –leu to 5-FOA –leu

Construct	Result regarding viability on 5-FOA	Number of (in)viable clones / total number of clones
Gal4$^{768-881}$ shuffle 3	viable	6 out of 6
Pho4$^{1-249}$ shuffle	inviable	6 out of 6
IDR2 WT broken helix	inviable	6 out of 6

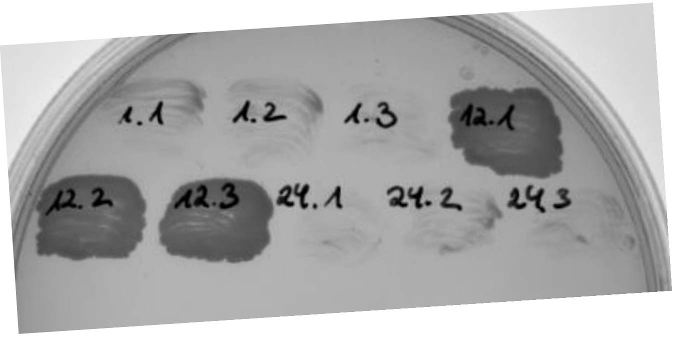

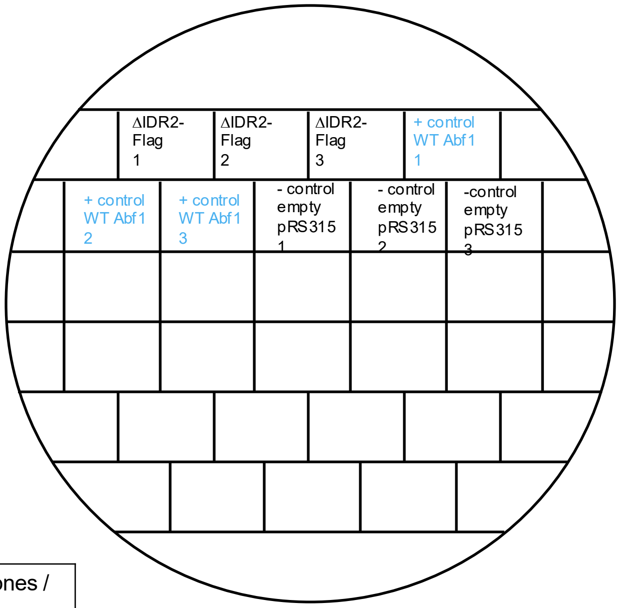

Restreak from YNB –ura –leu to 5-FOA –leu

Construct	Result regarding viability on 5-FOA	Number of (in)viable clones / total number of clones
ΔIDR2-Flag	inviable	3 out of 3

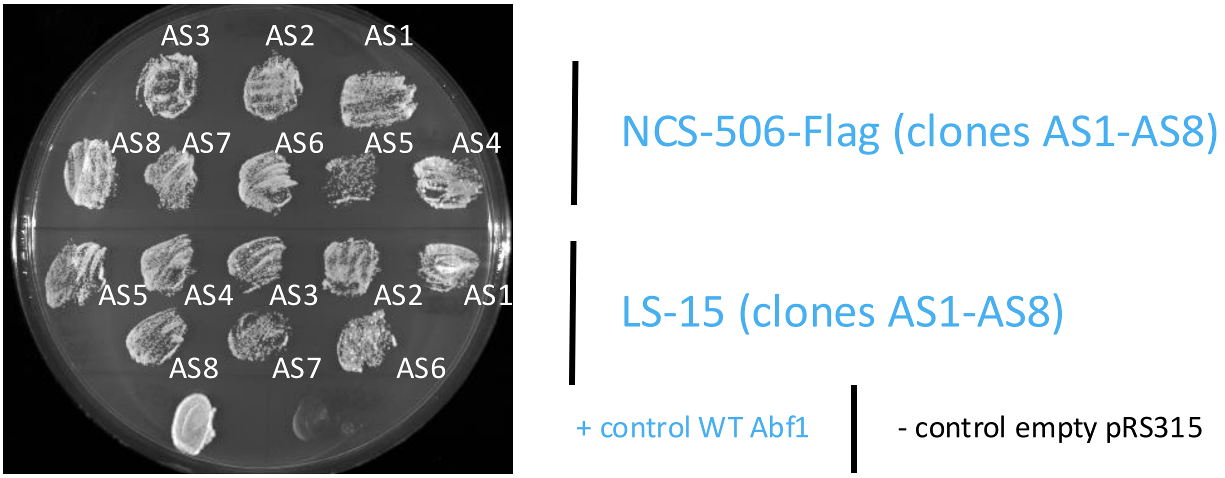

Restreak from YNB –ura –leu to 5-FOA –leu

Construct	Result regarding viability on 5-FOA	Number of (in)viable clones / total number of clones
NCS-506-Flag	viable	8 out of 8
LS-15	viable	8 out of 8 (in total 14 out of 14, see also slide 24)

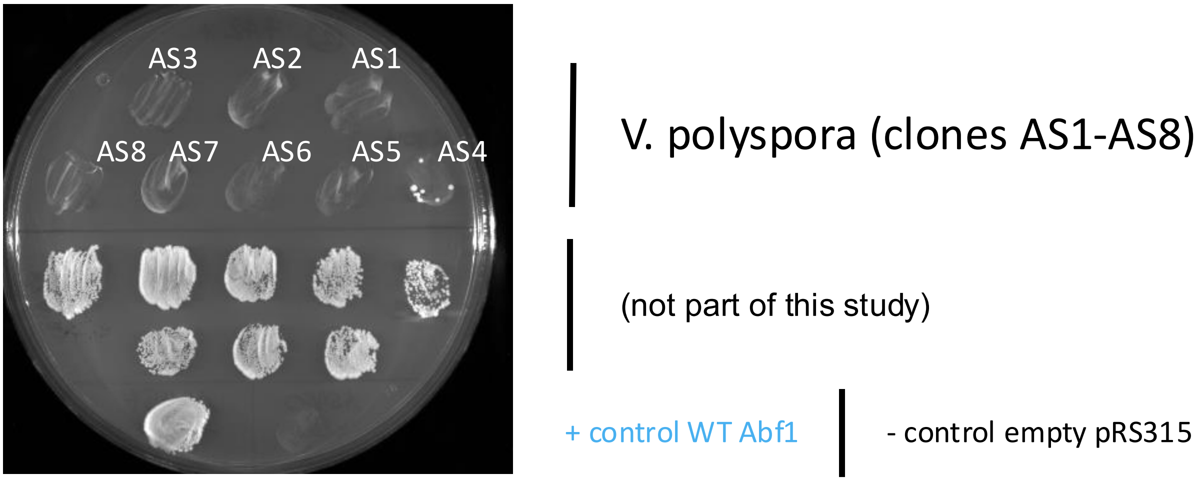

Restreak from YNB –ura –leu to 5-FOA –leu

Construct	Result regarding viability on 5-FOA	Number of (in)viable clones / total number of clones
V. polyspora	inviable	8 out of 8 (in total 14 out of 14, see also slide 17)

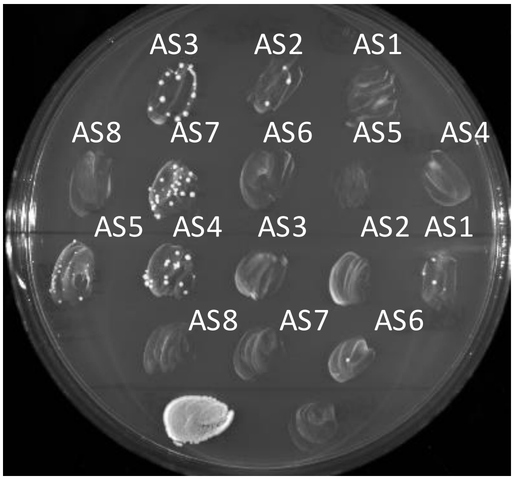

FUS$^{1\text{-}163}$12E + Abf1^{G4} distr.
(clones AS1-AS8)

FUS$^{1\text{-}163}$12E + EM shuffle
(clones AS1-AS8)

+ control WT Abf1 | - control empty pRS315

Restreak from YNB –ura –leu to 5-FOA –leu

Construct	Result regarding viability on 5-FOA	Number of (in)viable clones / total number of clones
FUS$^{1\text{-}163}$12E + Abf1^{G4} distr.	inviable	8 out of 8 (in total 14 out of 14, see also slide 40)
FUS$^{1\text{-}163}$12E + EM shuffle	inviable	8 out of 8 (in total 14 out of 14, see also slide 40)

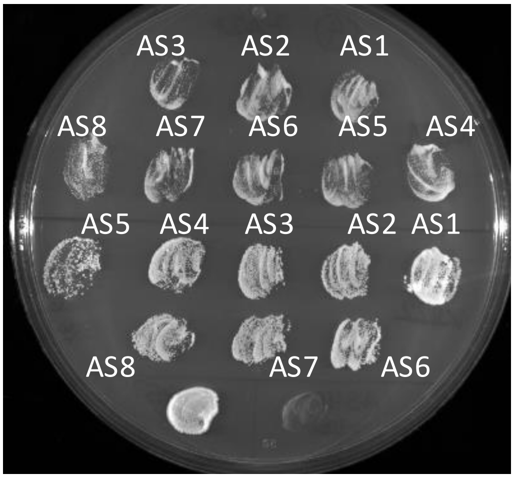

FUS$^{1\text{-}163}$12E + Gal4^{G4} all ΔE/D
(clones AS1-AS8)

NCS-21 hydro -> polar
(clones AS1-AS8)

+ control WT Abf1 | - control empty pRS315

Restreak from YNB –ura –leu to 5-FOA –leu

Construct	Result regarding viability on 5-FOA	Number of (in)viable clones / total number of clones
FUS$^{1\text{-}163}$12E + Gal4^{G4} all ΔE/D	viable	8 out of 8
NCS-21 hydro→polar	viable	8 out of 8

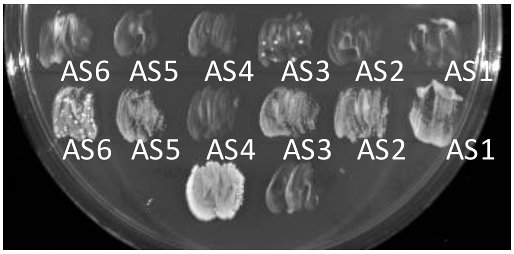

Pho4$^{1-249}$ segmental shuffle (clones AS1-AS6)

Sup35$^{1-131}$ + EM (clones AS1-AS6)

+ control WT Abf1 | - control empty pRS315

Restreak from YNB –ura –leu to 5-FOA –leu

Construct	Result regarding viability on 5-FOA	Number of (in)viable clones / total number of clones
Pho4$^{1-249}$ segmental shuffle	inviable	6 out of 6
Sup35$^{1-131}$ + EM	viable	5 out of 6

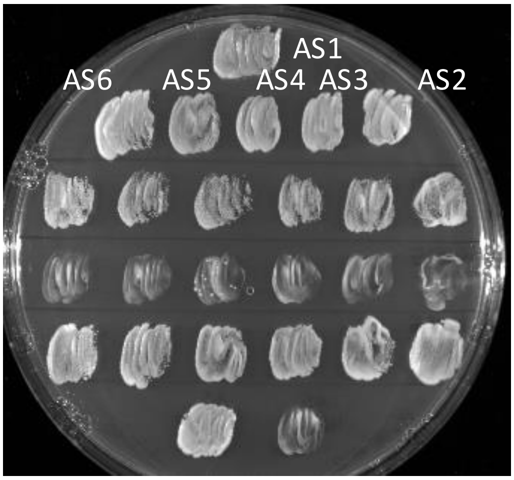

FUS$^{1-163}$12E + Gal4^{G4} distr. II (clones AS1-AS6)

(not part of this study)

+ control WT Abf1 | - control empty pRS315

Restreak from YNB –ura –leu to 5-FOA –leu

Construct	Result regarding viability on 5-FOA	Number of (in)viable clones / total number of clones
FUS$^{1-163}$12E Gal4^{G4} distr. II	viable	6 out of 6

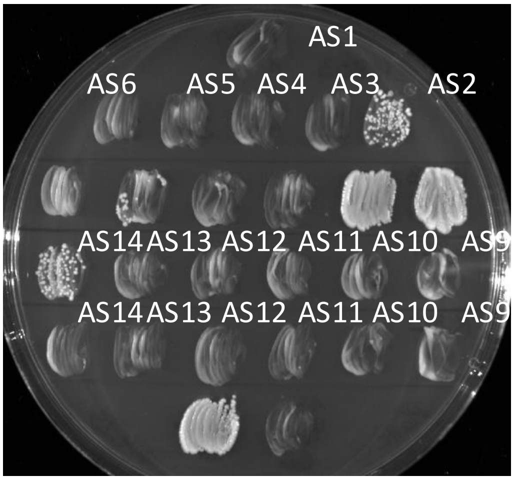

Rap1 IDR$^{1-120}$ & IDR$^{230-361}$ (clones AS1-AS6)

(not part of this study)

FUS$^{1-163}$12E + Abf1^{G4} distr. (clones AS9-AS14)

FUS$^{1-163}$12E + EM shuffle (clones AS9-AS14)

+ control WT Abf1 | - control empty pRS315

Restreak from YNB –ura –leu to 5-FOA –leu

Construct	Result regarding viability on 5-FOA	Number of (in)viable clones / total number of clones
Rap1$^{1-120\ \&\ 230-361}$	inviable	6 out of 6 (in total 7 out of 8, see also slide 22)
FUS$^{1-163}$12E + Abf1^{G4} distr.	inviable	6 out of 6 (in total 14 out of 14, see also slide 38)
FUS$^{1-163}$12E + EM shuffle	inviable	6 out of 6 (in total 14 out of 14, see also slide 38)

Supplementary Figure S2: **Growth scores for constructs**

Unless noted otherwise, the following images show YPDA plates that were incubated for ca. 1 day at 30 °C. All strains carried the indicated Abf1 constructs on pRS315, while the chromosomal *ABF1* gene was deleted. If one of the three independent clones after transformation for each construct did not show the same growth rate as the others, it is labeled as „outlier“ and we categorized the growth rate according to the two similarily growing clones. All rating is relative to the growth of the strain with wild type (WT) Abf1 on the same plate.

growth rate score

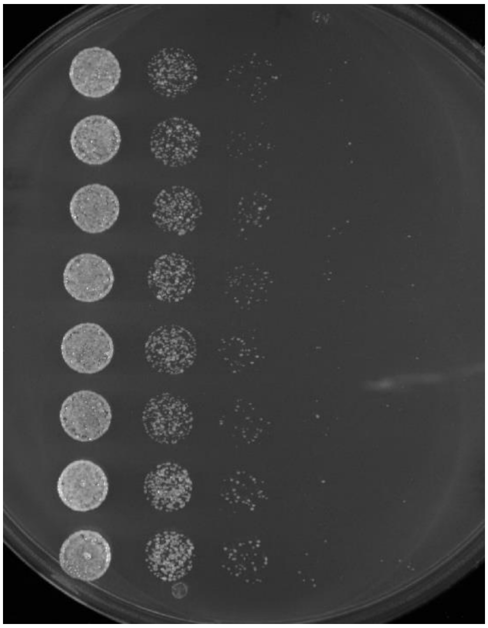

++++ FUS$^{1\text{-}163}$12E + GR

++++ FUS$^{1\text{-}163}$12E + Gal4^{M2}

++++ Abf1 WT

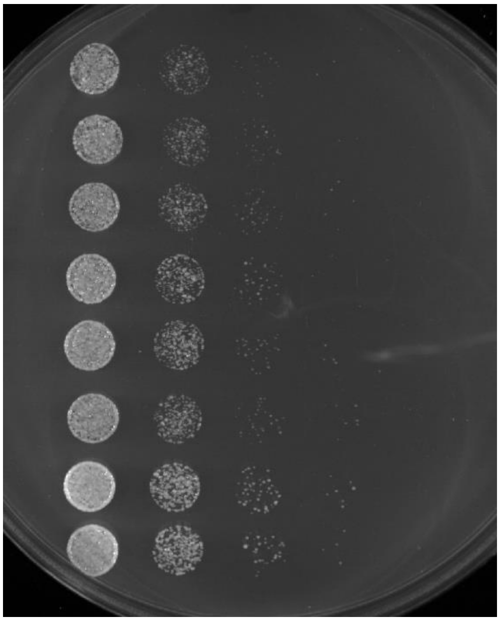

++++ FUS$^{1\text{-}163}$12E + VP16

++++ FUS$^{1\text{-}163}$12E + Gcn4

++++ Abf1 WT

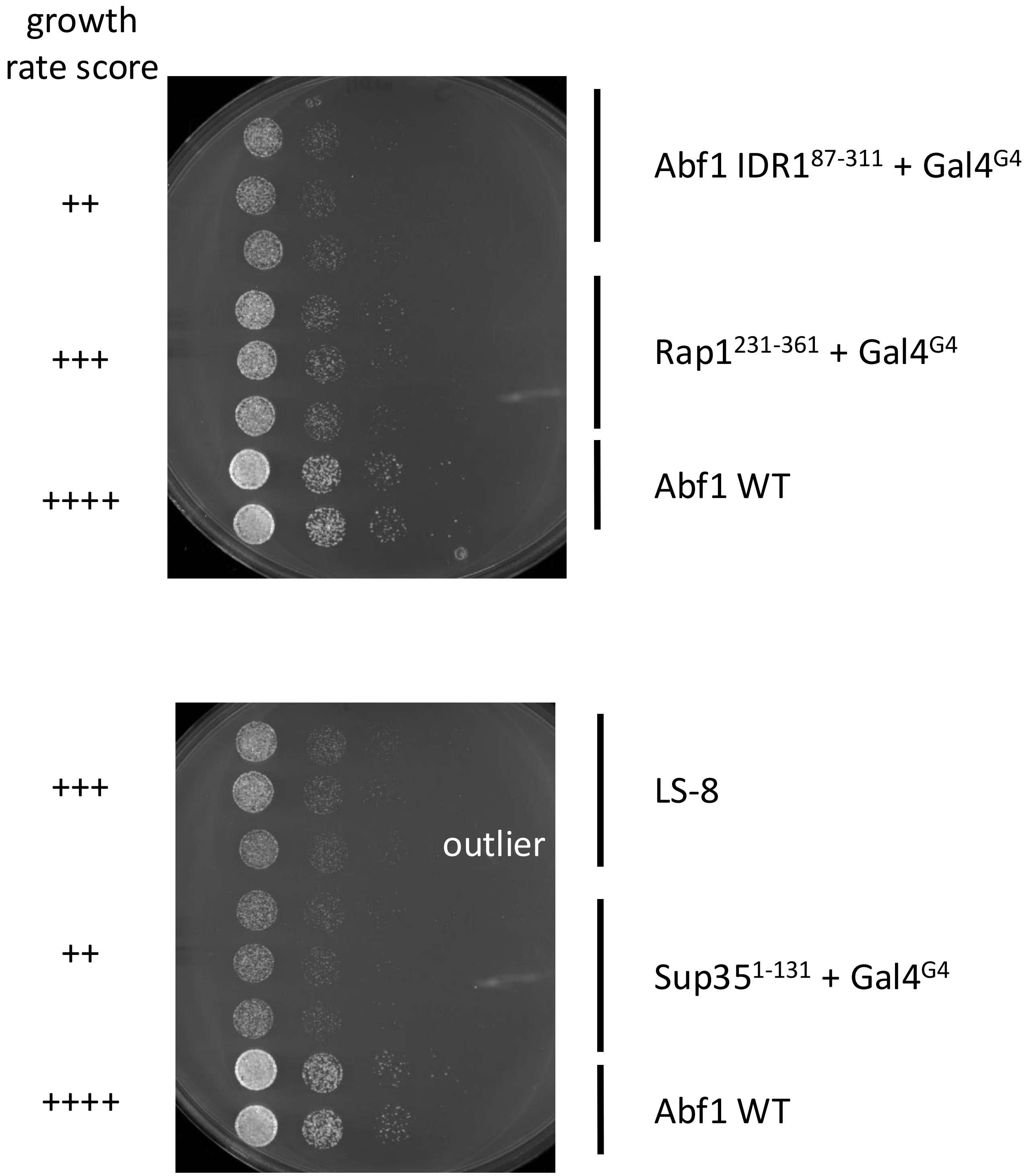
growth rate score
++
+++
++++
Abf1 IDR1$^{87-311}$ + Gal4^{G4}
Rap1$^{231-361}$ + Gal4^{G4}
Abf1 WT
+++
++
++++
outlier
LS-8
Sup35$^{1-131}$ + Gal4^{G4}
Abf1 WT

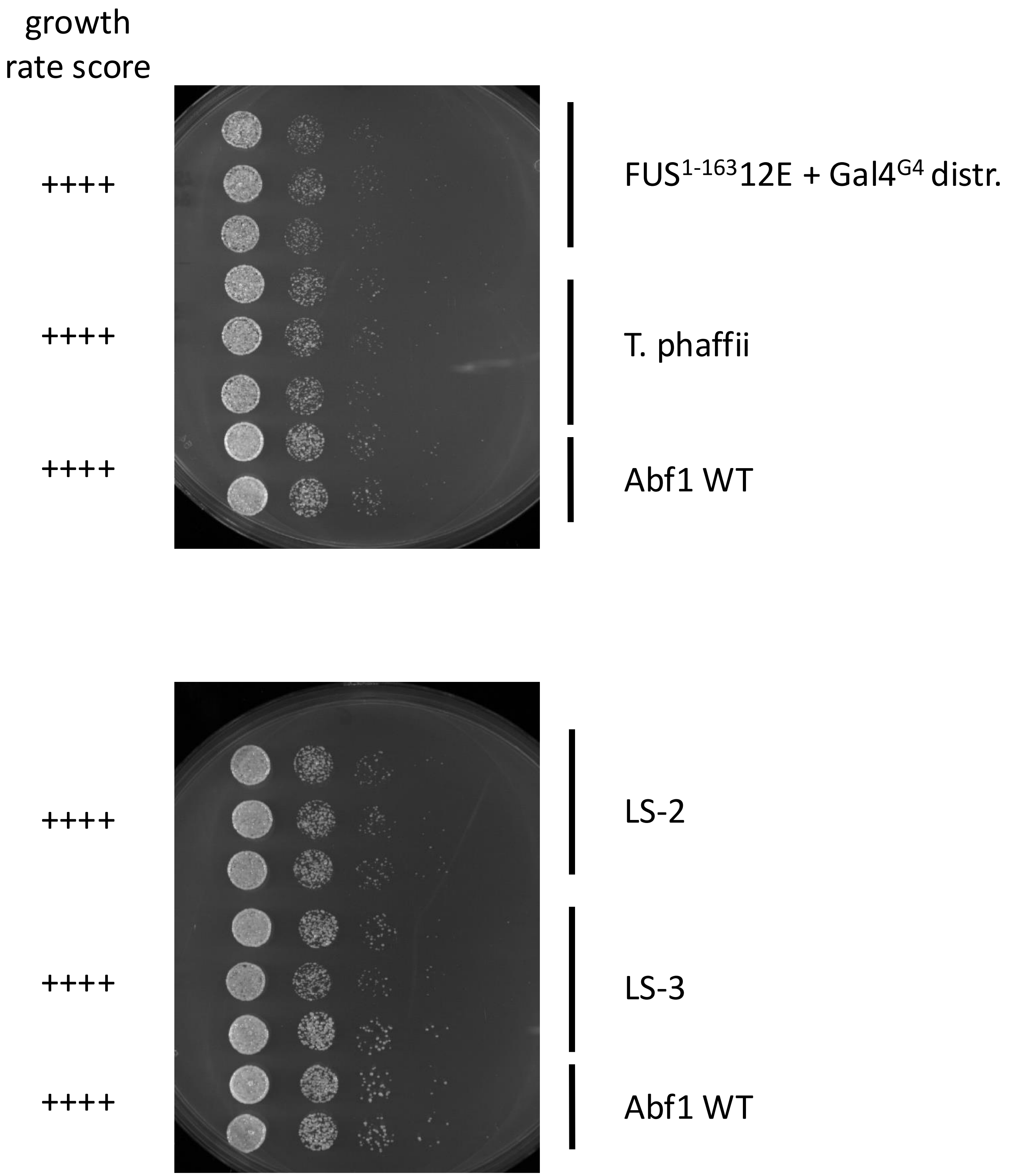
growth
rate score
++++
FUS1-163 12E + Gal4G4 distr.
++++
T. phaffii
++++
Abf1 WT
++++
LS-2
++++
LS-3
++++
Abf1 WT

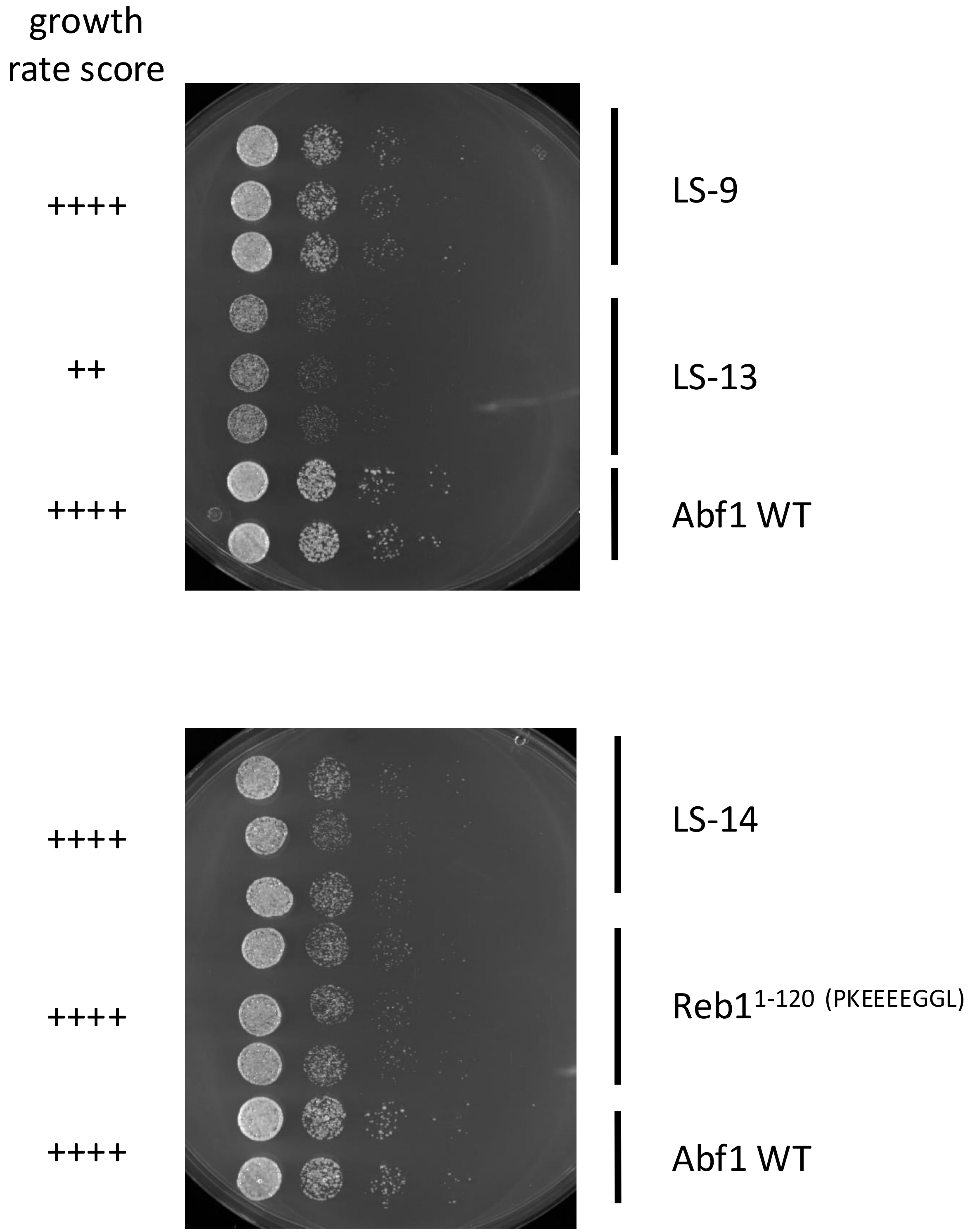

growth rate score
++++
LS-9
++
LS-13
++++
Abf1 WT
++++
LS-14
++++
Reb1$^{1-120}$ (PKEEEEGGL)
++++
Abf1 WT

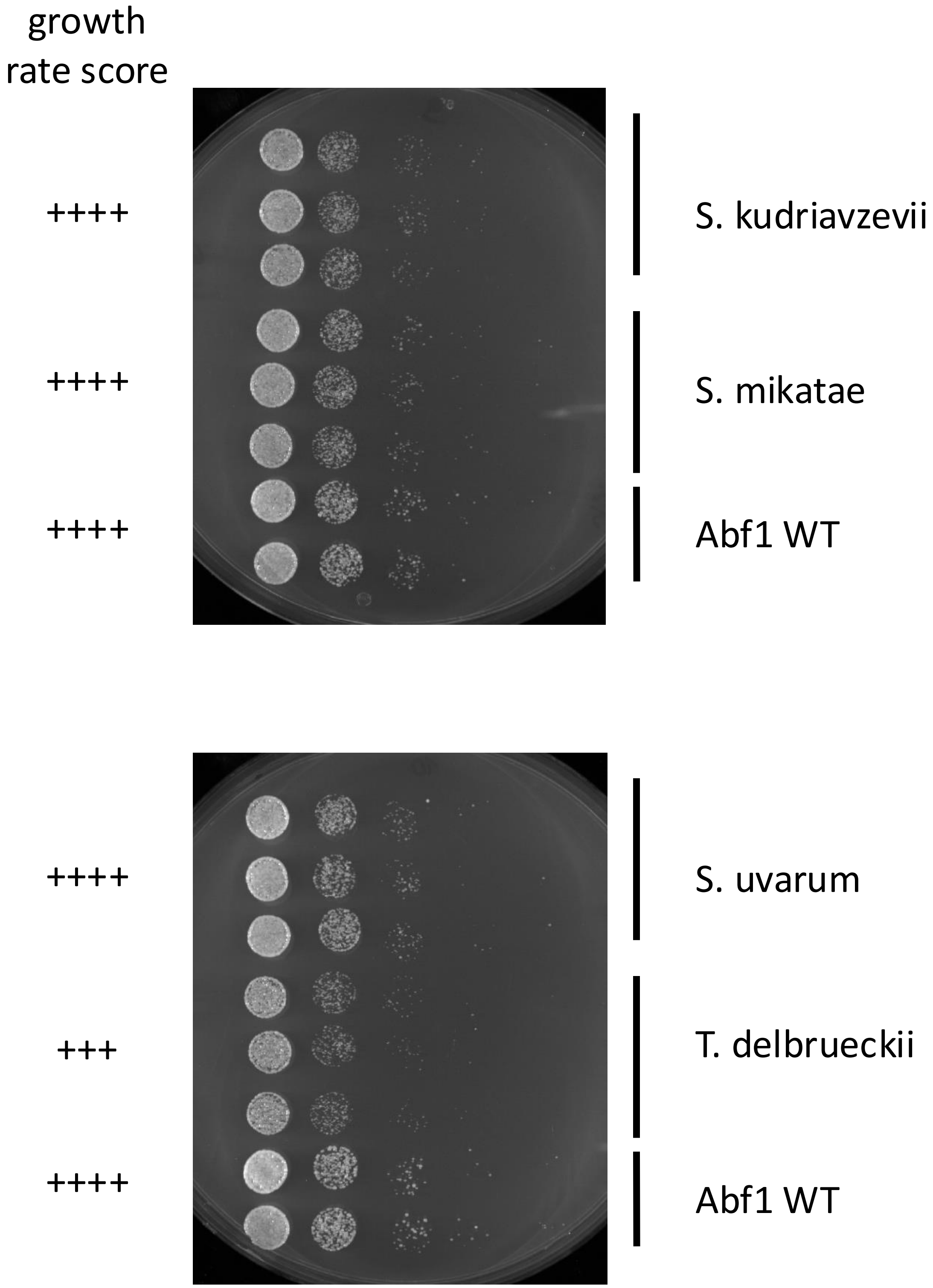
growth rate score
++++
S. kudriavzevii
++++
S. mikatae
++++
Abf1 WT
++++
S. uvarum
+++
T. delbrueckii
++++
Abf1 WT

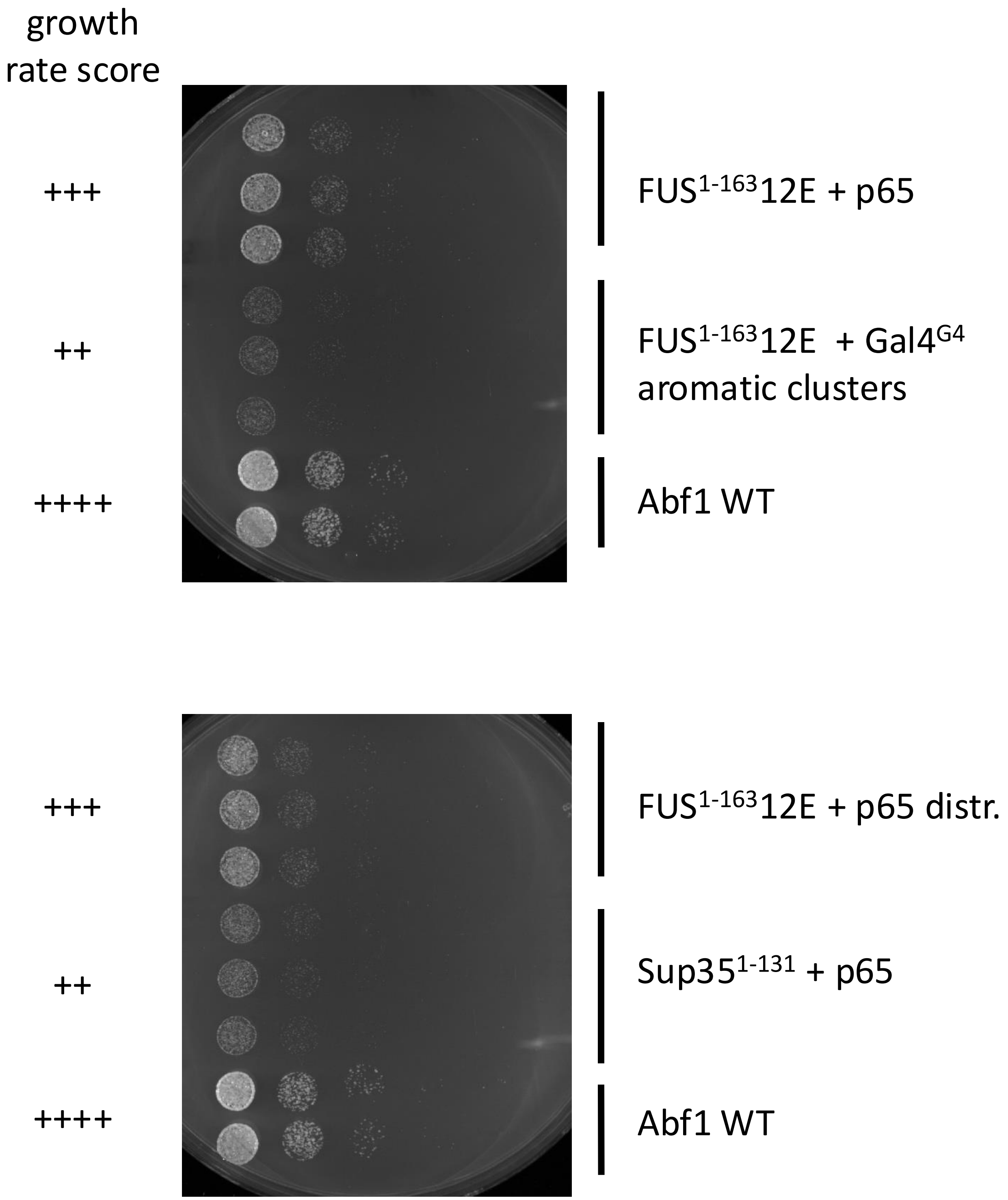
growth rate score
+++
FUS$^{1-163}$12E + p65
++
FUS$^{1-163}$12E + Gal4^{G4} aromatic clusters
++++
Abf1 WT
+++
FUS$^{1-163}$12E + p65 distr.
++
Sup35$^{1-131}$ + p65
++++
Abf1 WT

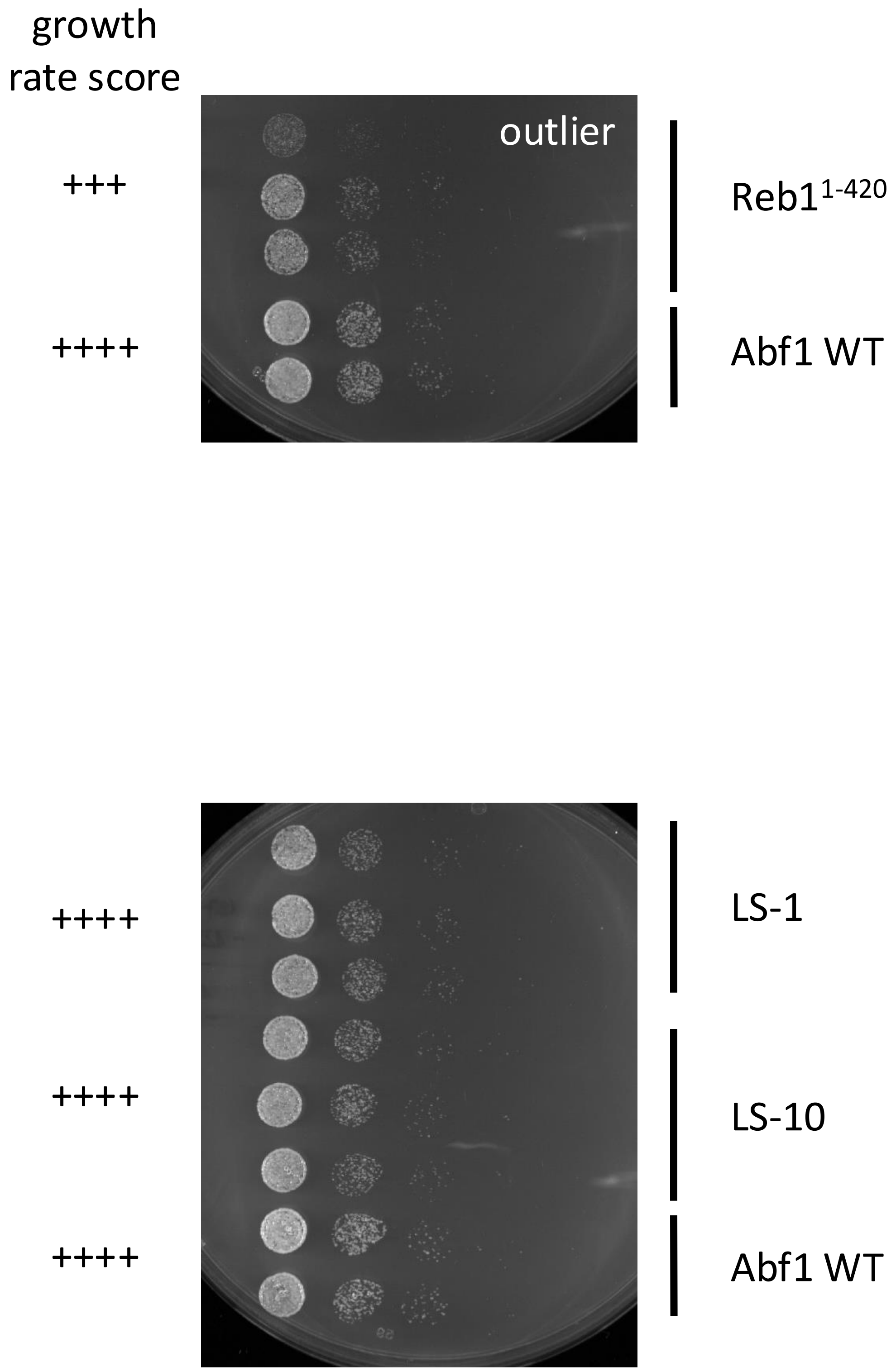
growth rate score
outlier
+++
Reb1^1-420
++++
Abf1 WT
++++
LS-1
++++
LS-10
++++
Abf1 WT

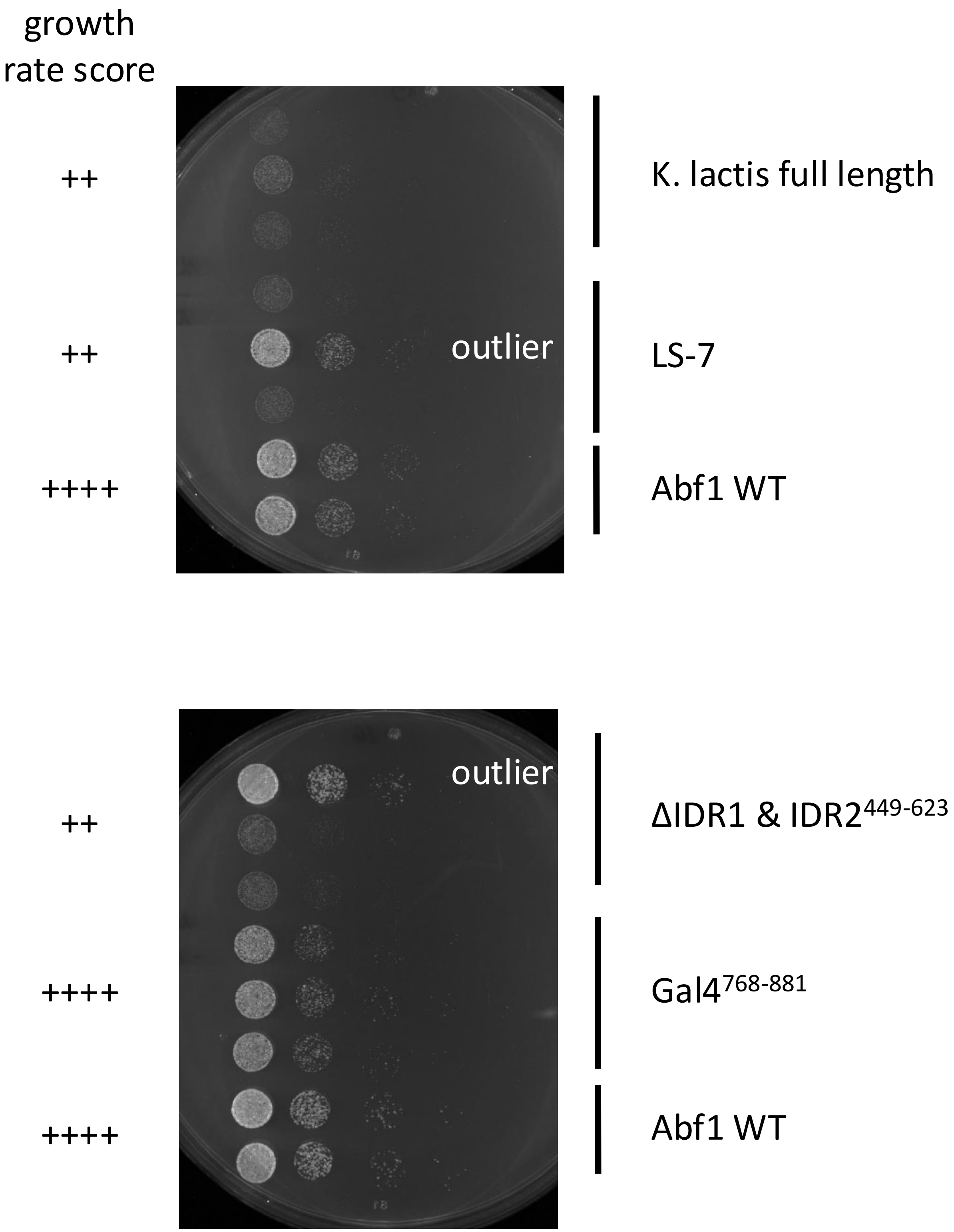
growth rate score
++
K. lactis full length
++
outlier
LS-7
++++
Abf1 WT
outlier
++
ΔIDR1 & IDR2$^{449\text{-}623}$
++++
Gal4$^{768\text{-}881}$
++++
Abf1 WT

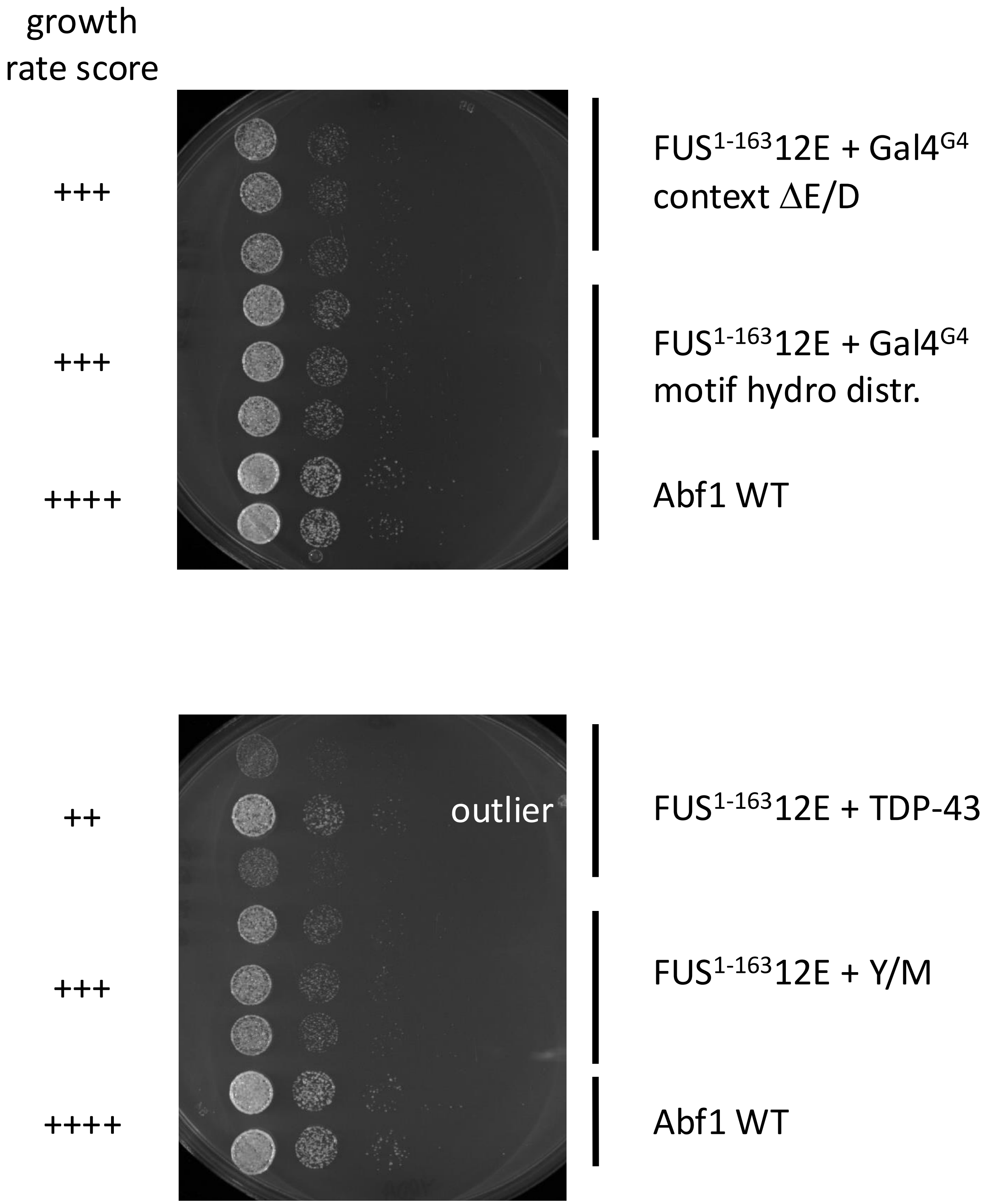
growth rate score
+++
FUS$^{1-163}$12E + Gal4^{G4} context ΔE/D
+++
FUS$^{1-163}$12E + Gal4^{G4} motif hydro distr.
++++
Abf1 WT
++
outlier
FUS$^{1-163}$12E + TDP-43
+++
FUS$^{1-163}$12E + Y/M
++++
Abf1 WT

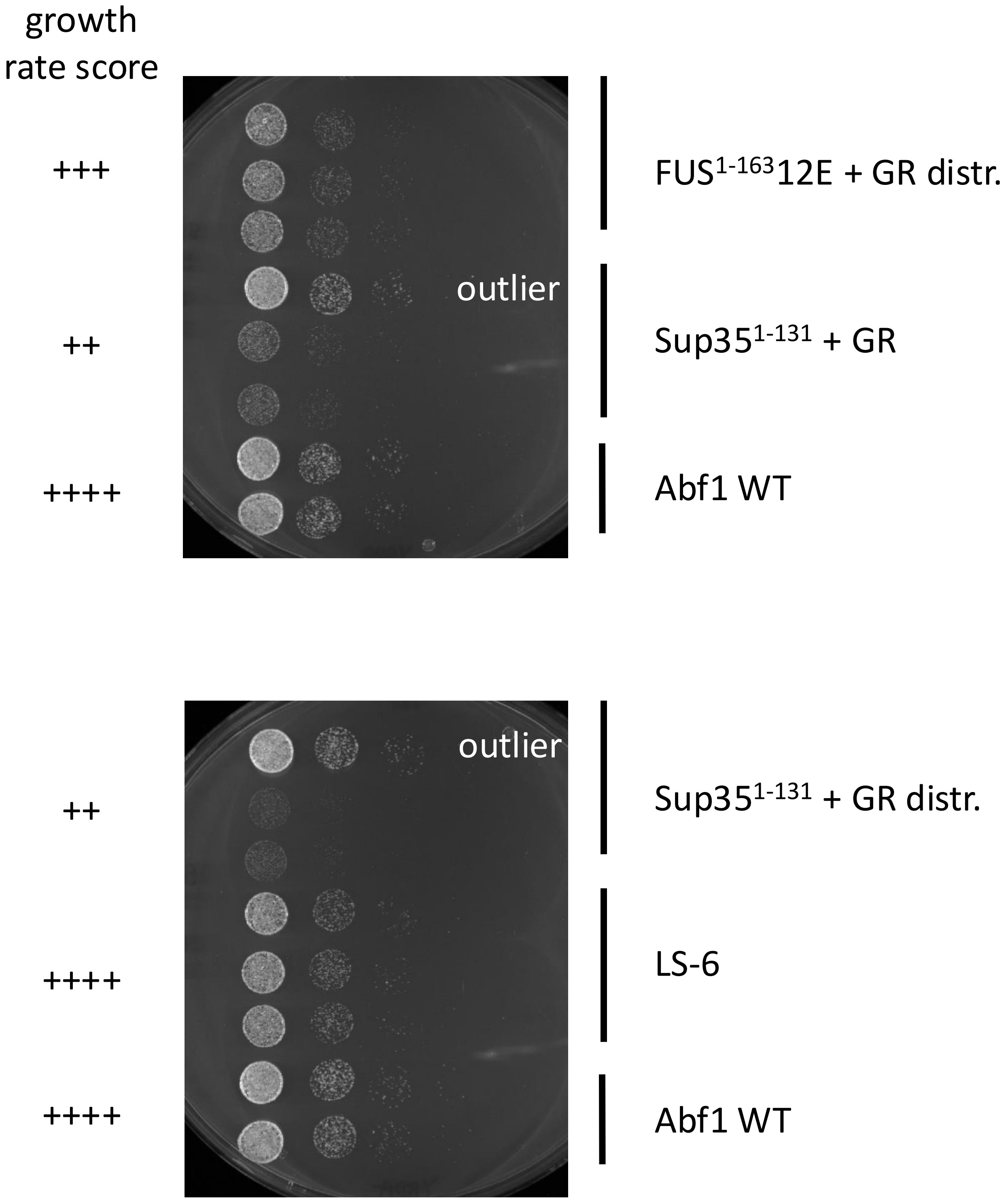
growth
rate score
+++
FUS$^{1-163}$12E + GR distr.
outlier
++
Sup35$^{1-131}$ + GR
++++
Abf1 WT
outlier
++
Sup35$^{1-131}$ + GR distr.
++++
LS-6
++++
Abf1 WT

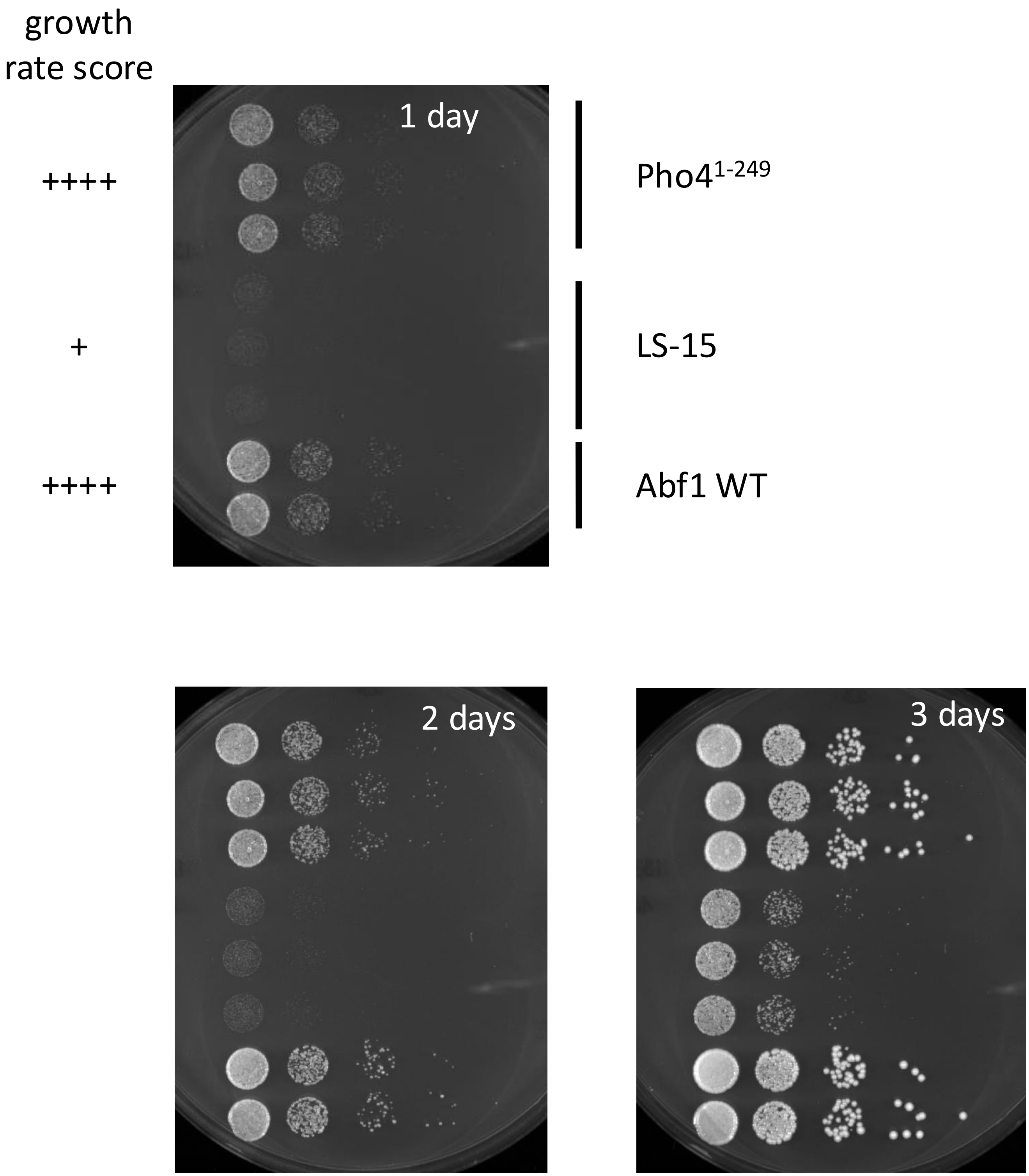
growth rate score
++++
+
++++
1 day
Pho4$^{1-249}$
LS-15
Abf1 WT
2 days
3 days

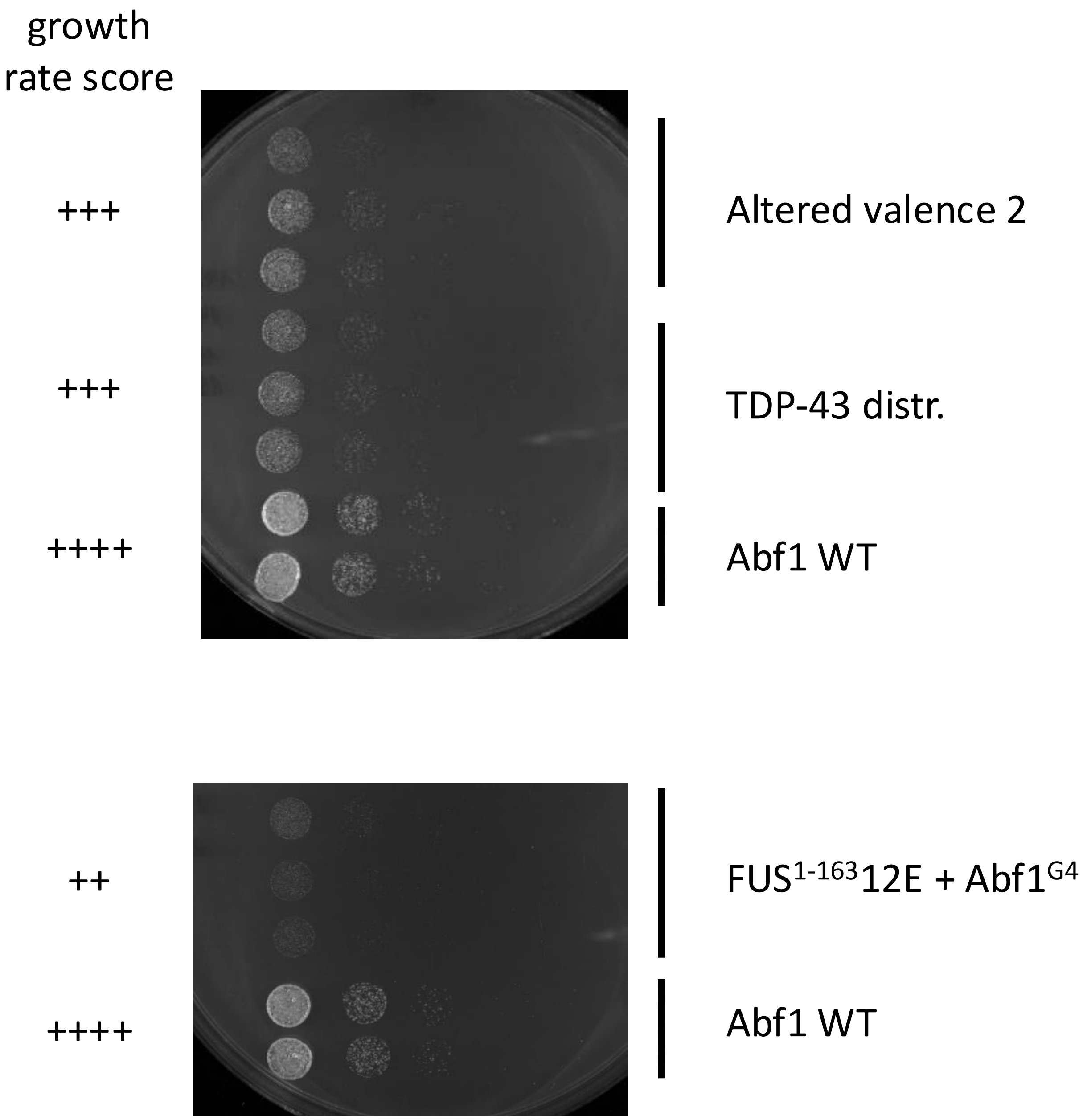
growth rate score
+++
Altered valence 2
+++
TDP-43 distr.
++++
Abf1 WT
++
FUS1-163 12E + Abf1G4
++++
Abf1 WT

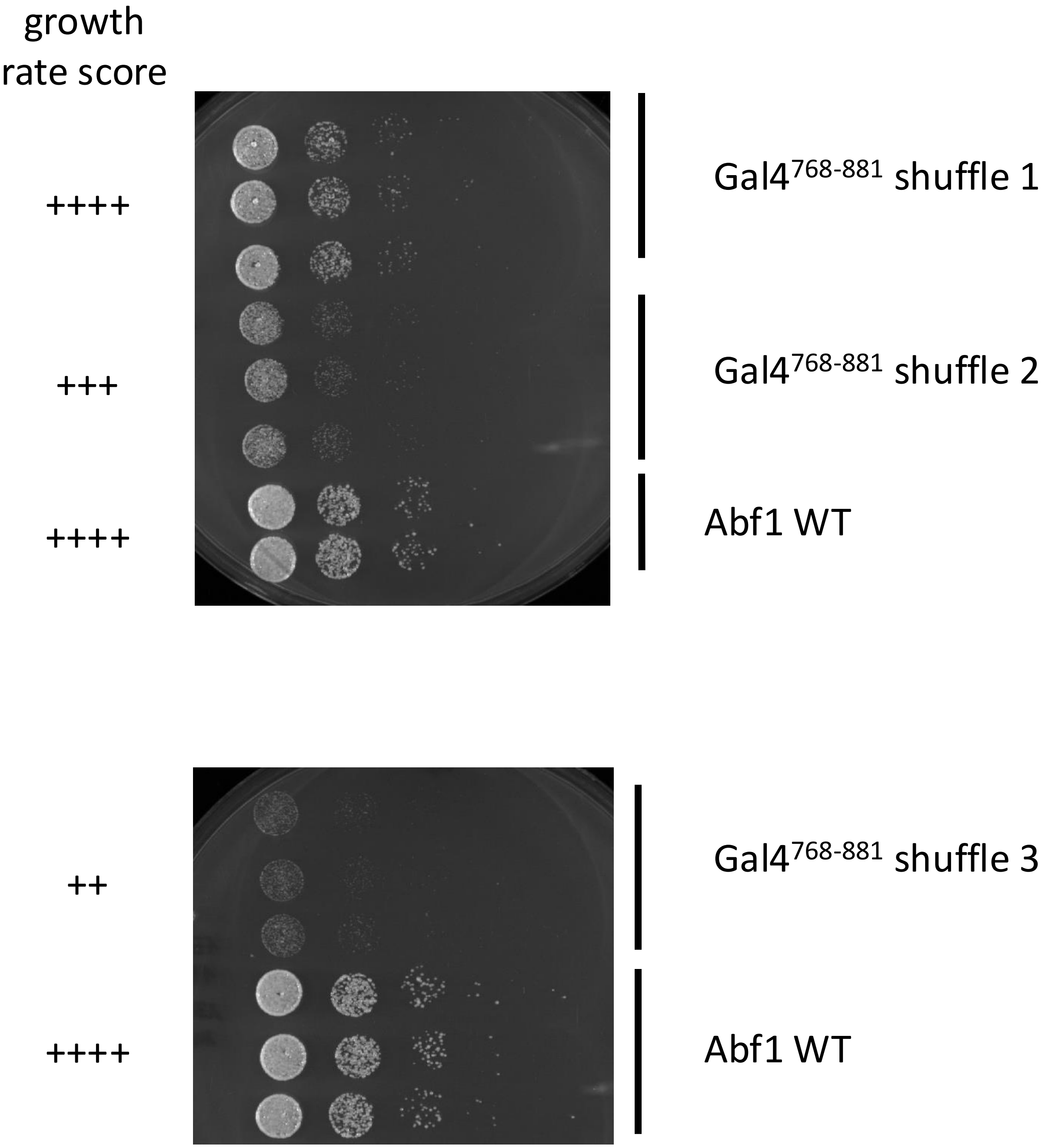
growth
rate score
++++
+++
++++
++
++++
Gal4$^{768-881}$ shuffle 1
Gal4$^{768-881}$ shuffle 2
Abf1 WT
Gal4$^{768-881}$ shuffle 3
Abf1 WT

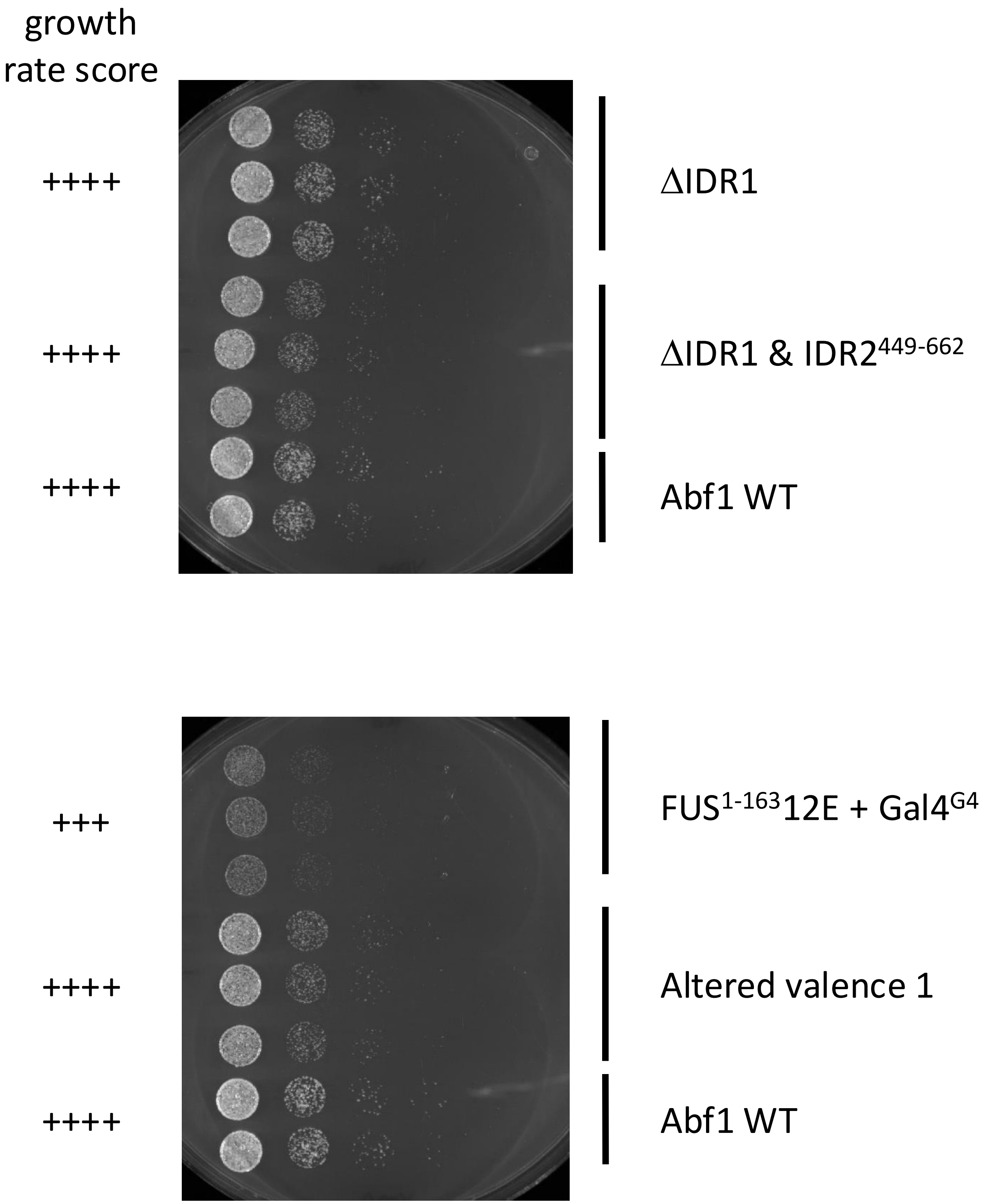
growth
rate score
++++
ΔIDR1
++++
ΔIDR1 & IDR2 449-662
++++
Abf1 WT
+++
FUS 1-163 12E + Gal4 G4
++++
Altered valence 1
++++
Abf1 WT

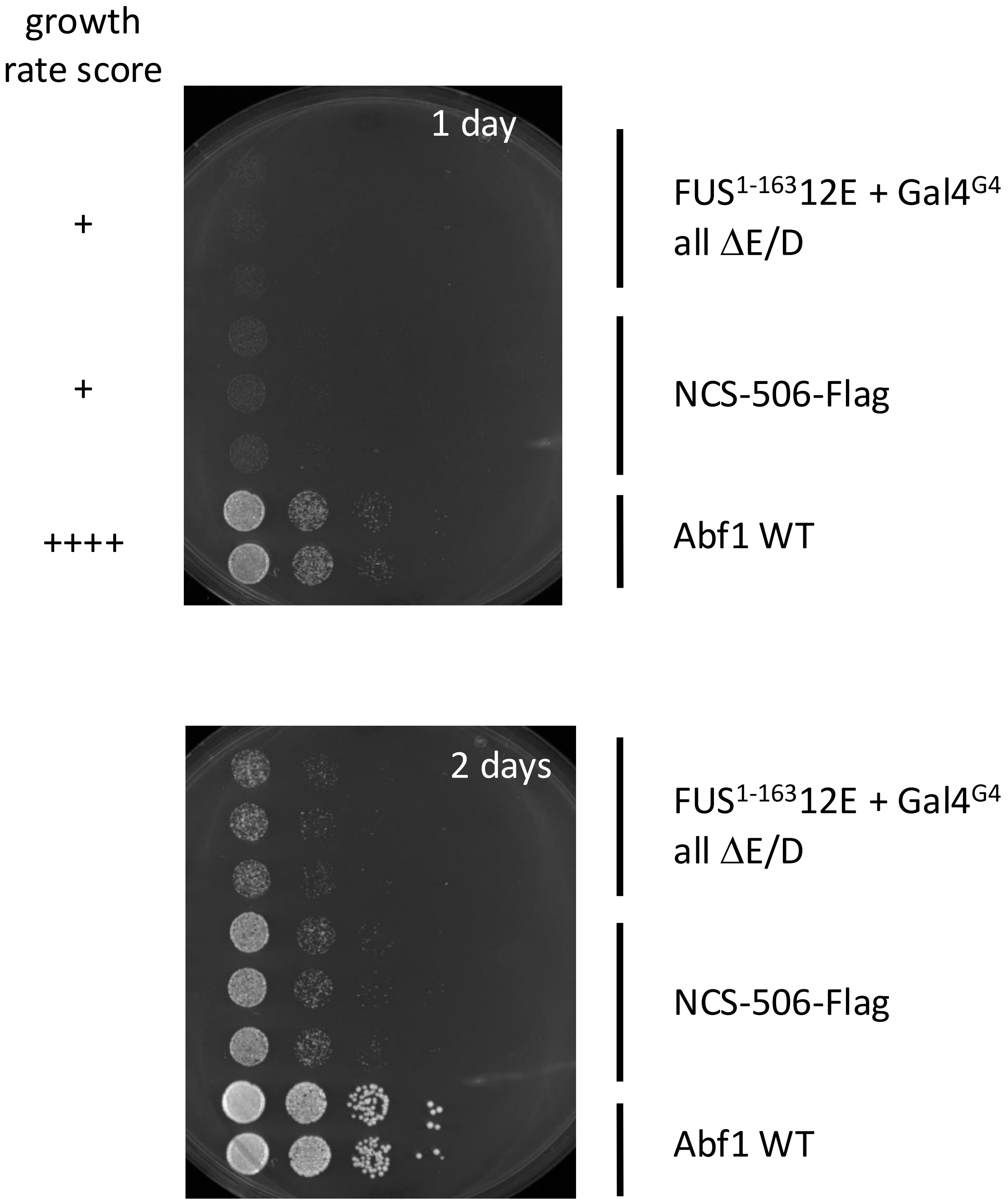
growth
rate score
1 day
+
+
++++
FUS$^{1-163}$12E + Gal4^{G4}
all ΔE/D
NCS-506-Flag
Abf1 WT
2 days
FUS$^{1-163}$12E + Gal4^{G4}
all ΔE/D
NCS-506-Flag
Abf1 WT

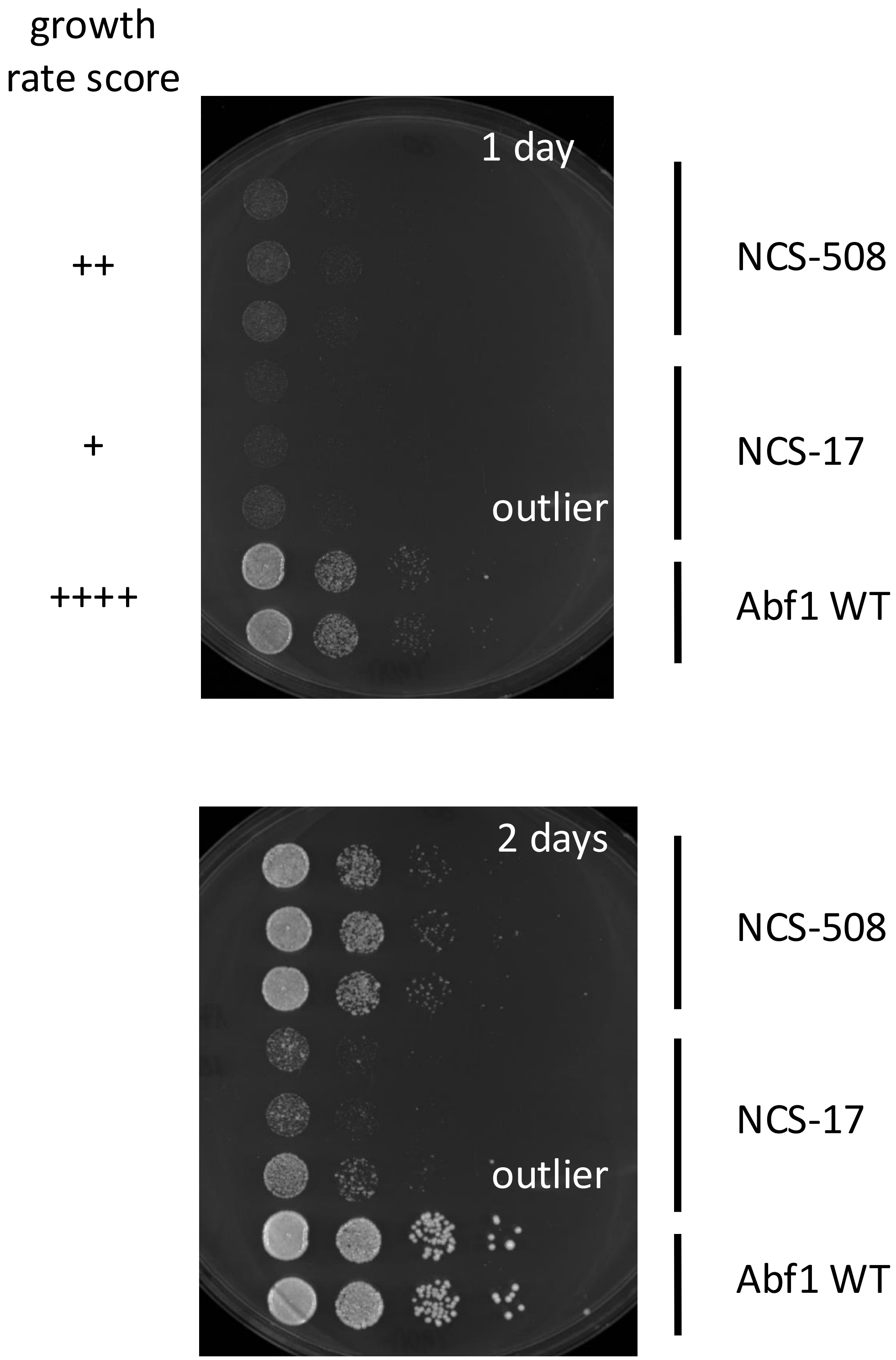
growth
rate score
++
+
++++
1 day
outlier
2 days
outlier
NCS-508
NCS-17
Abf1 WT
NCS-508
NCS-17
Abf1 WT

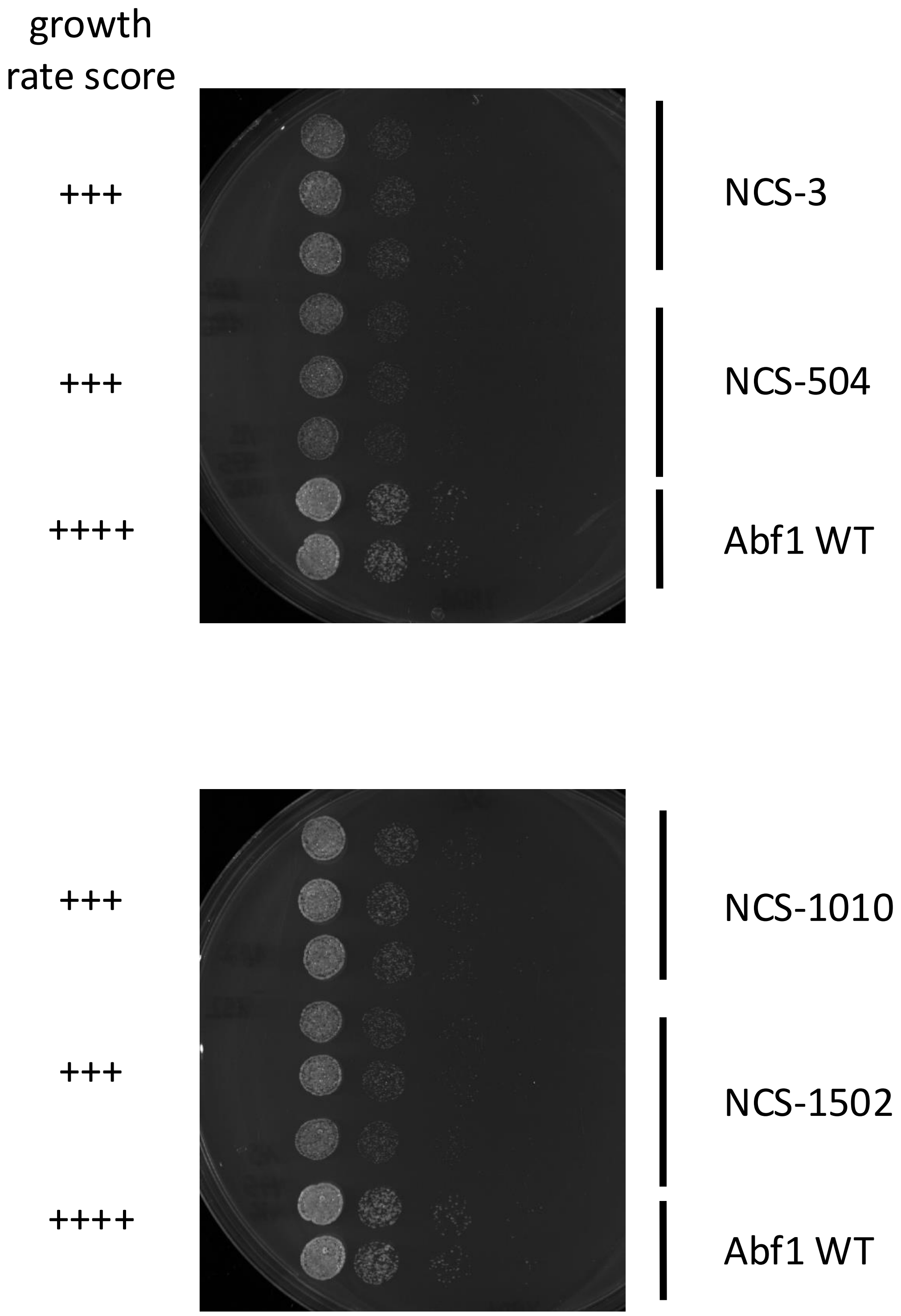
growth
rate score
+++
NCS-3
+++
NCS-504
++++
Abf1 WT
+++
NCS-1010
+++
NCS-1502
++++
Abf1 WT

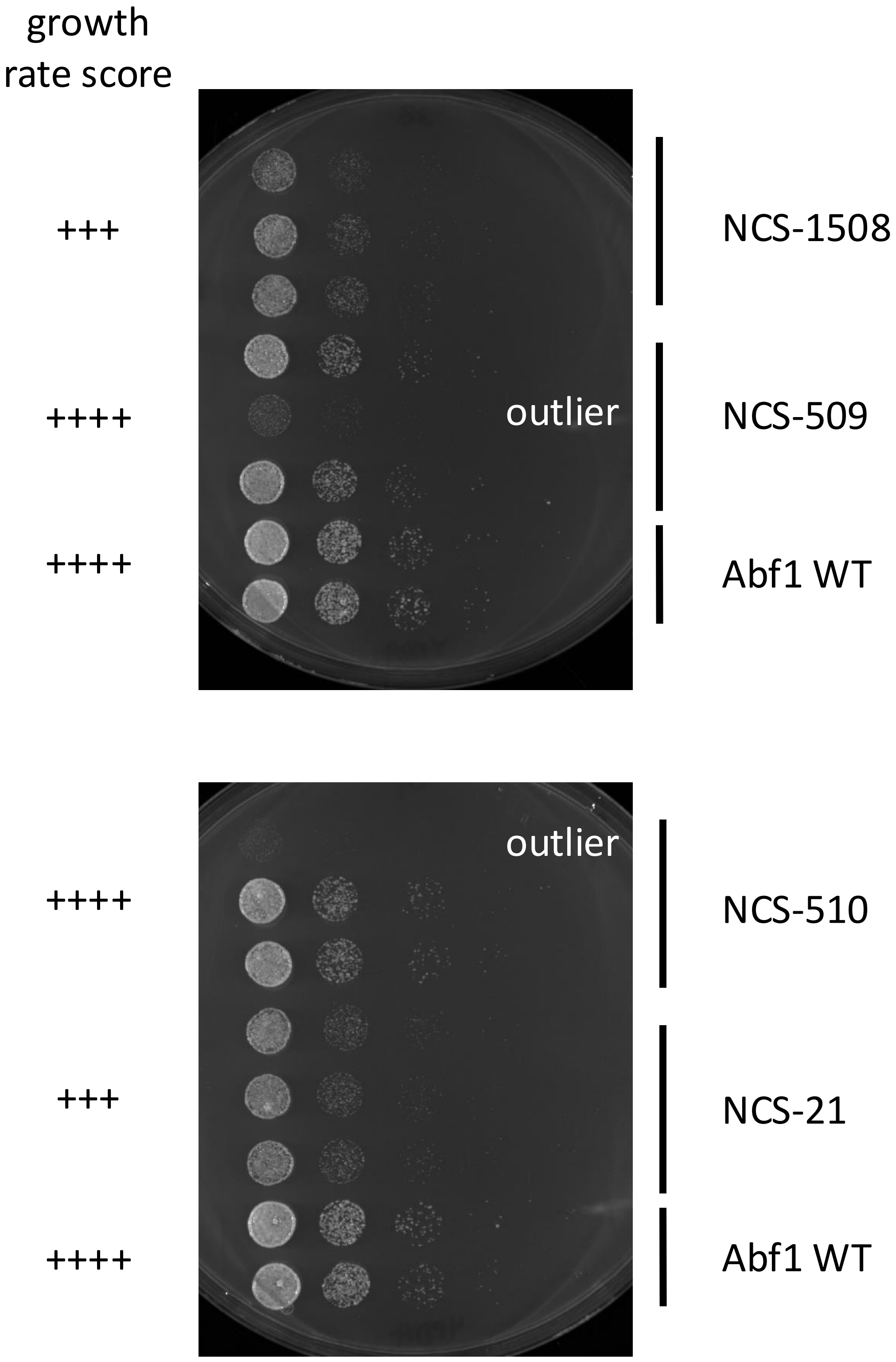
growth rate score
+++
NCS-1508
++++
outlier
NCS-509
++++
Abf1 WT
outlier
++++
NCS-510
+++
NCS-21
++++
Abf1 WT

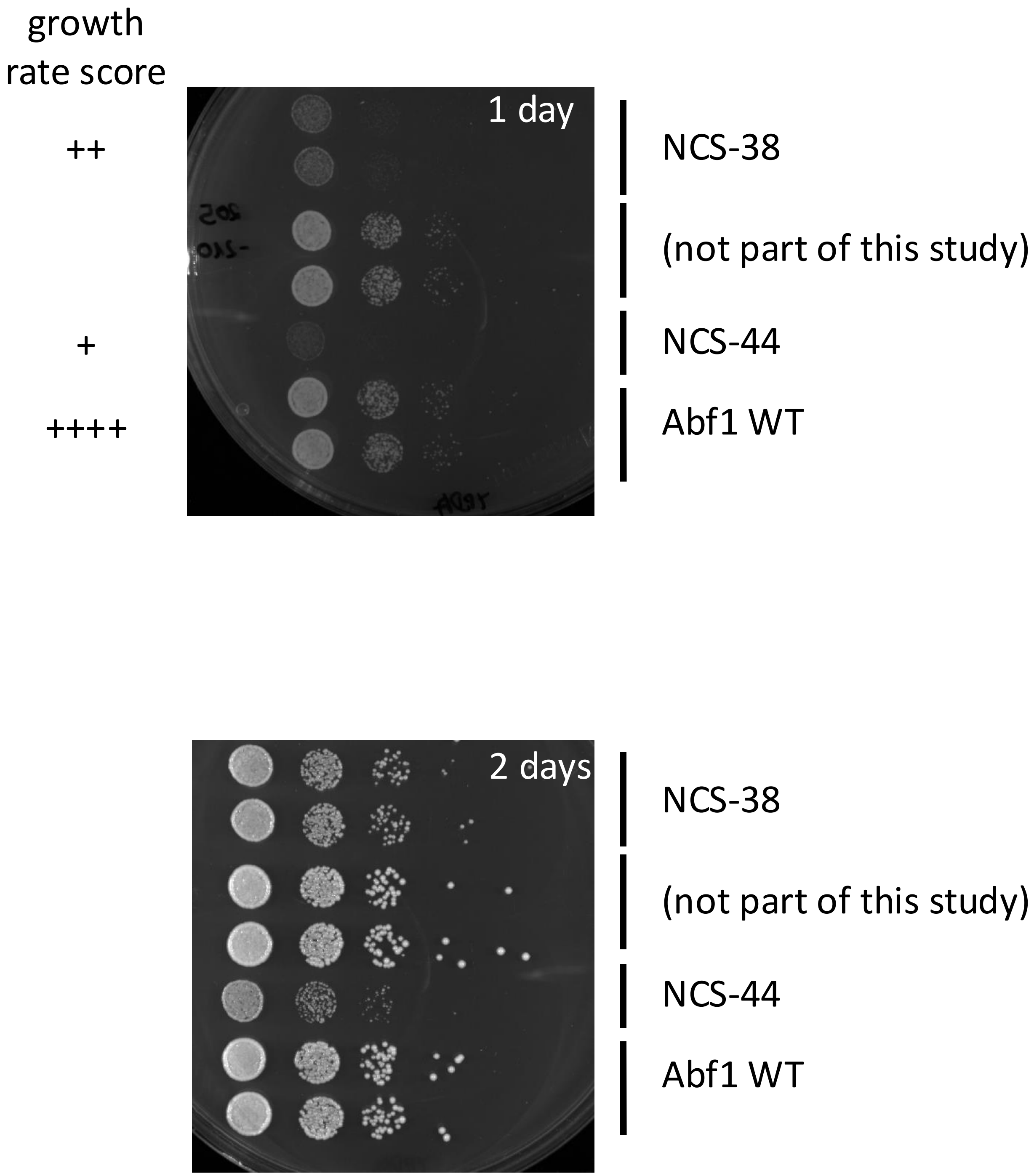
growth rate score
1 day
++
NCS-38
(not part of this study)
+
NCS-44
++++
Abf1 WT
2 days
NCS-38
(not part of this study)
NCS-44
Abf1 WT

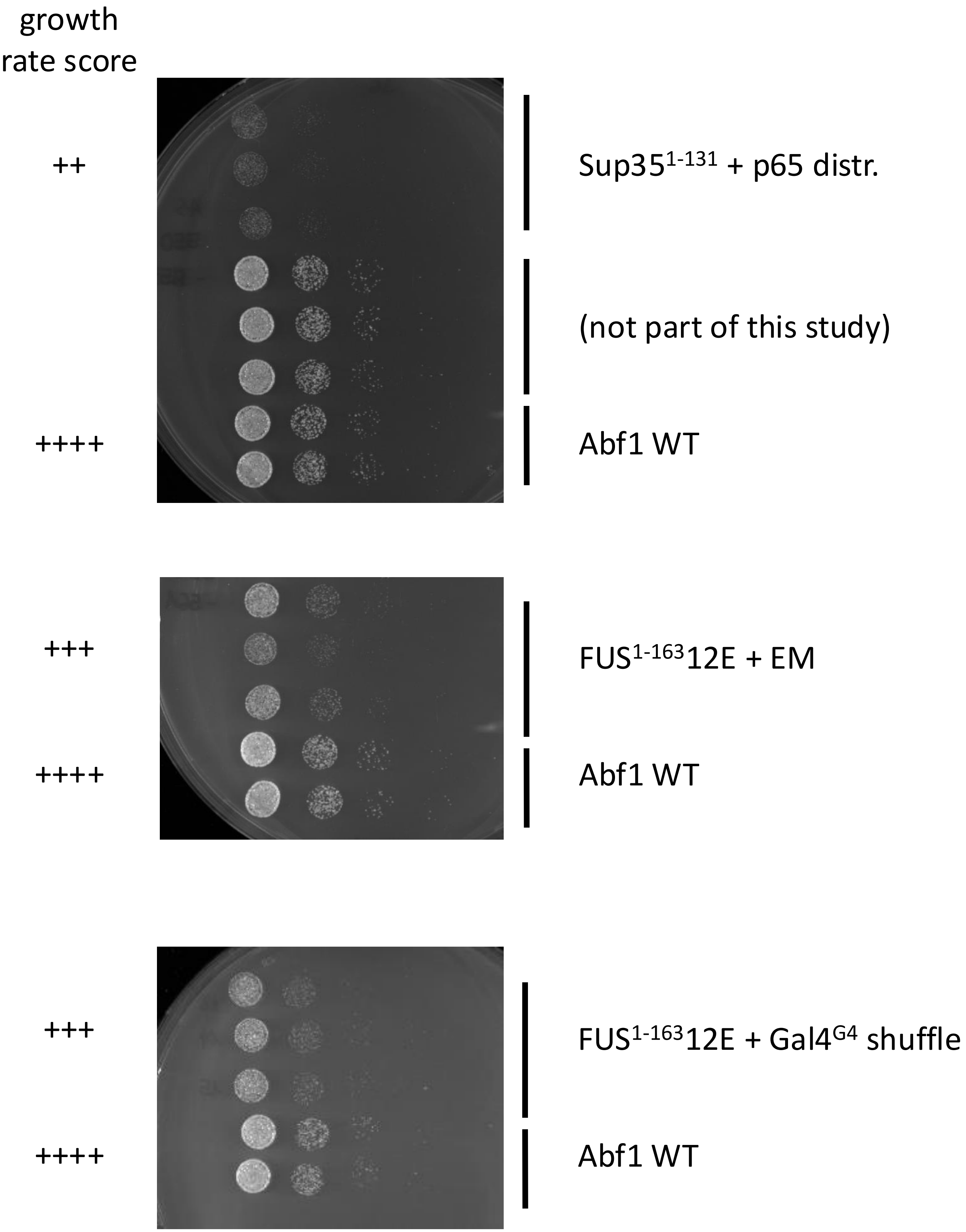
growth rate score
++
Sup35$^{1-131}$ + p65 distr.
(not part of this study)
++++
Abf1 WT
+++
FUS$^{1-163}$12E + EM
++++
Abf1 WT
+++
FUS$^{1-163}$12E + Gal4^{G4} shuffle
++++
Abf1 WT

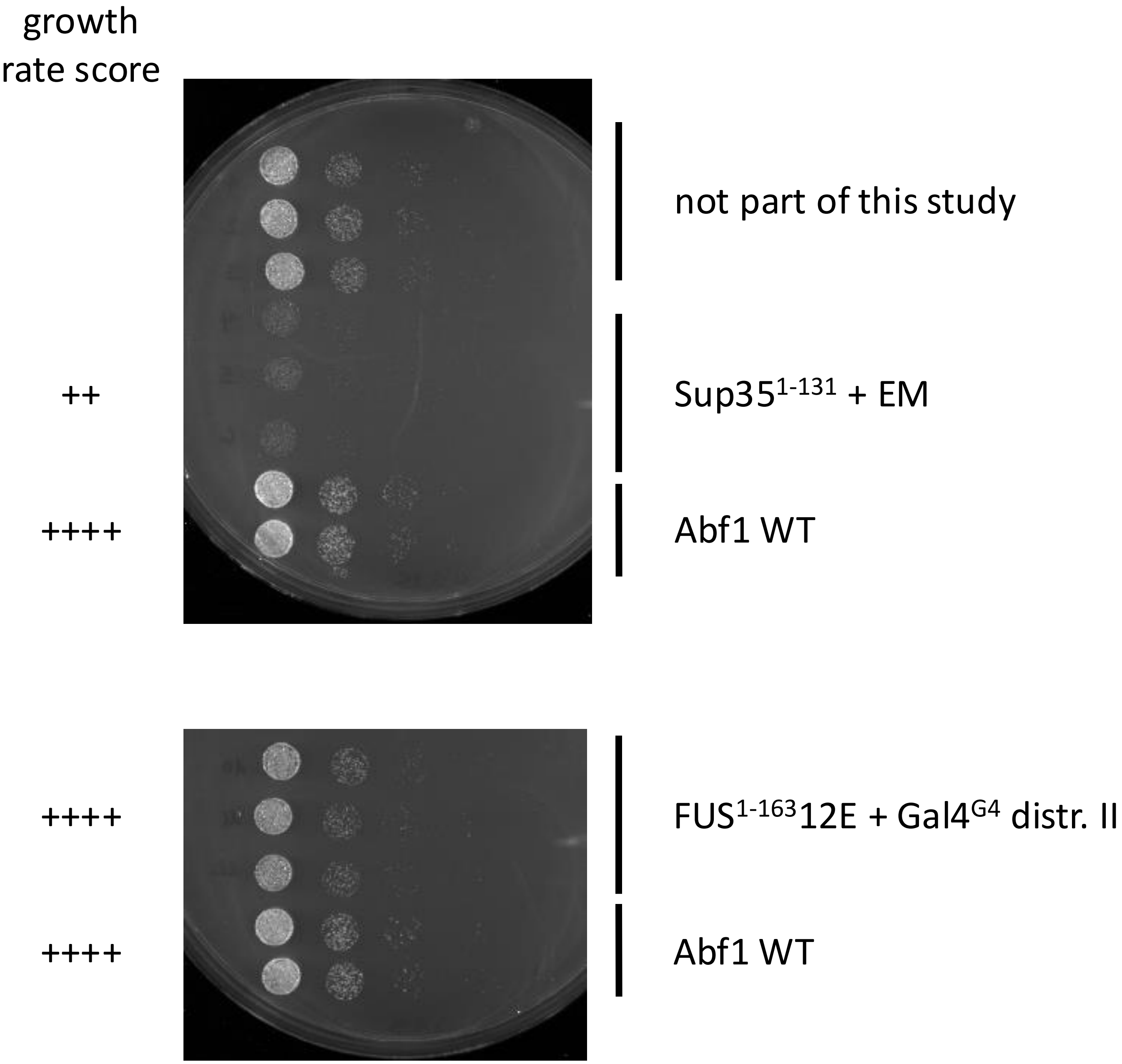
growth rate score
not part of this study
++
Sup35$^{1-131}$ + EM
++++
Abf1 WT
++++
FUS$^{1-163}$12E + Gal4^{G4} distr. II
++++
Abf1 WT

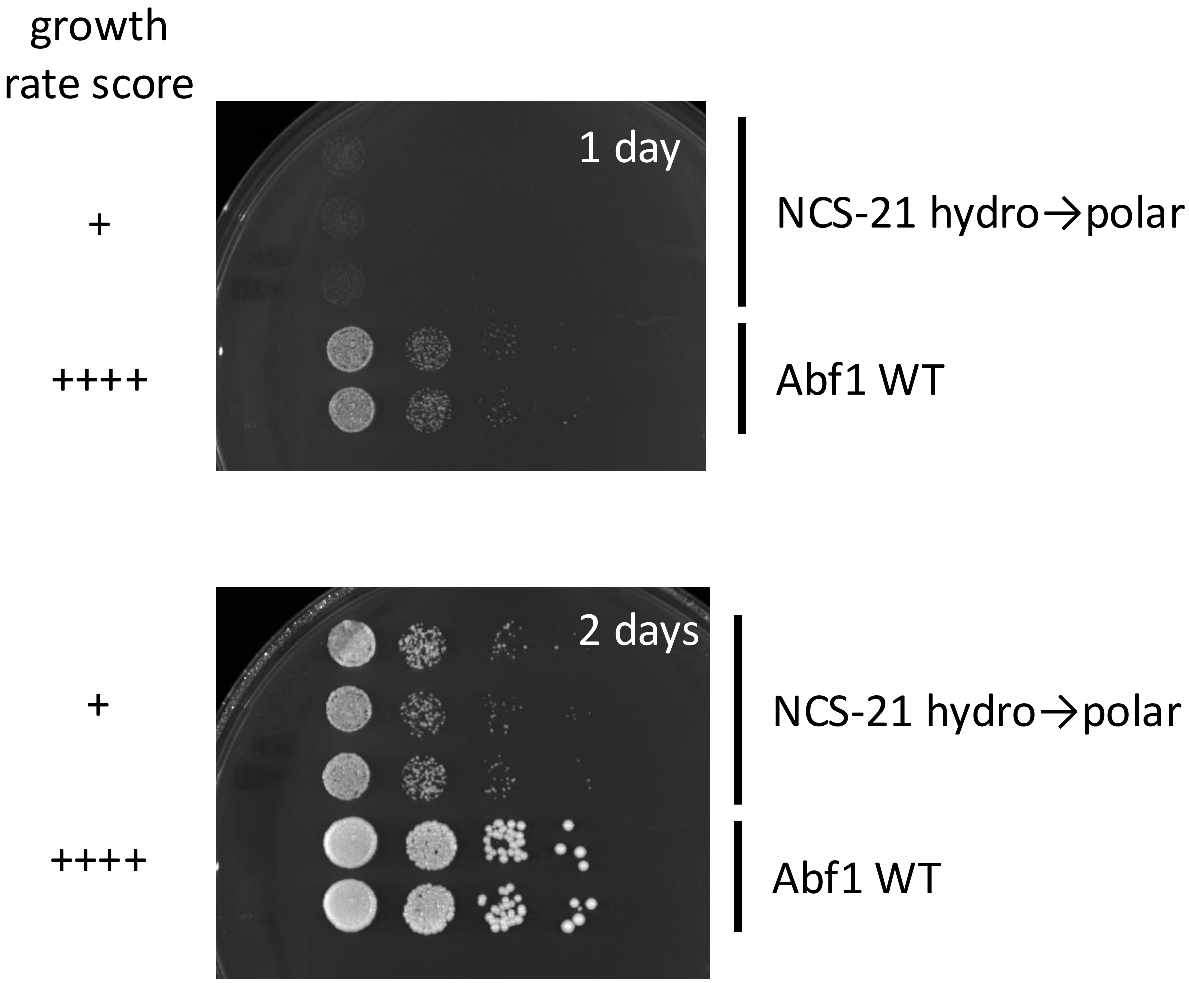
growth
rate score
1 day
+
NCS-21 hydro→polar
++++
Abf1 WT
2 days
+
NCS-21 hydro→polar
++++
Abf1 WT

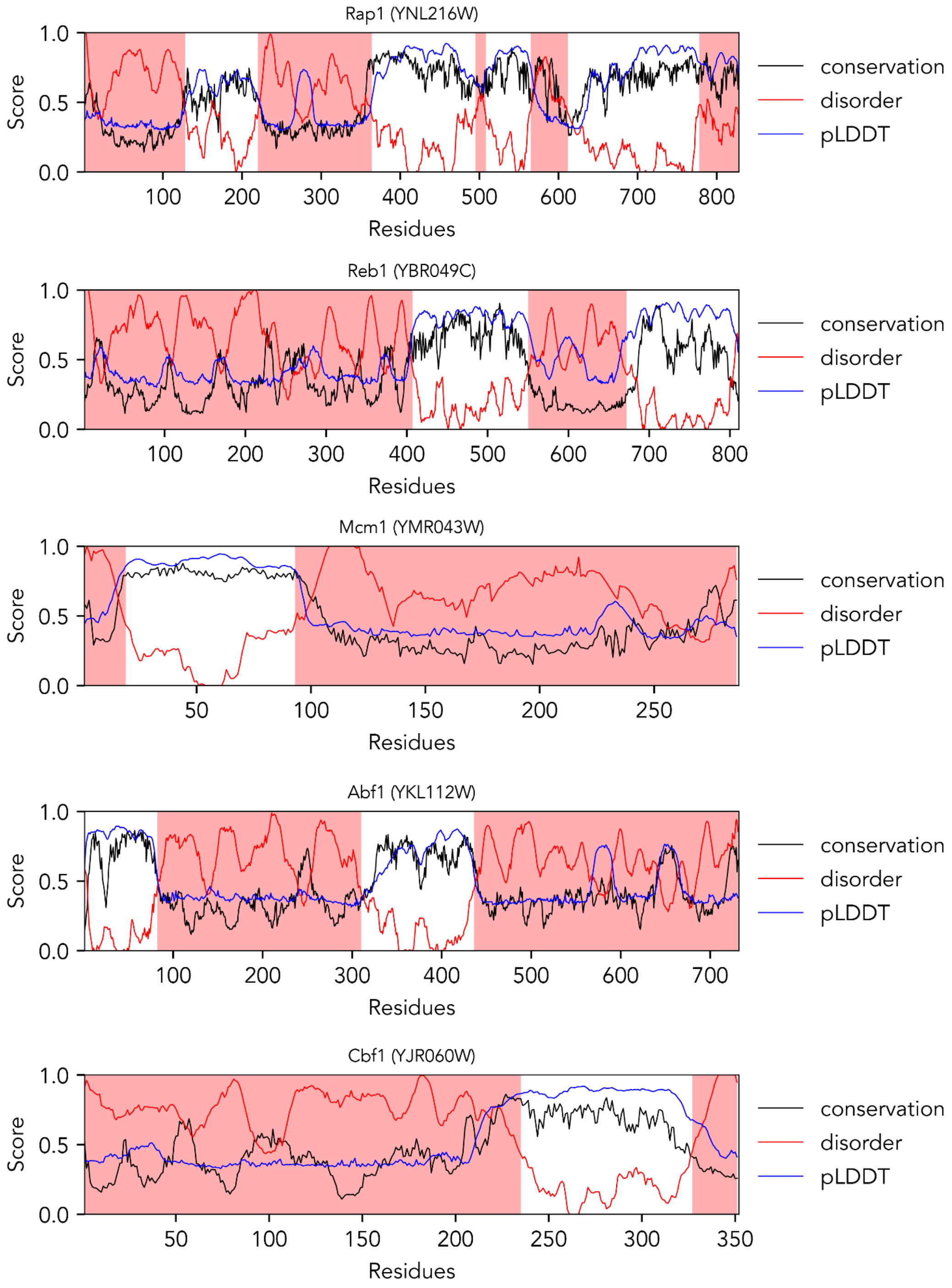

Supplementary Figure 3. Disorder, conservation, and predicted structure profiles for representative general regulatory factor (GRF) proteins. We analyzed linear sequence profiles for Rap1, Reb1, Mcm1, Abf1, and Cbf1. The shaded red regions are contiguous IDRs, the red line denotes the per-residue disorder score, the blue line denotes the per-residue predicted pLDDT score, and the black line denotes the per-residue conservation score. Disorder is predicted using metapredict V1 (see Methods for justification of this over metapredict V2 or V3). Predicted pLDDT score reports on the likelihood of structure (see *Methods*).

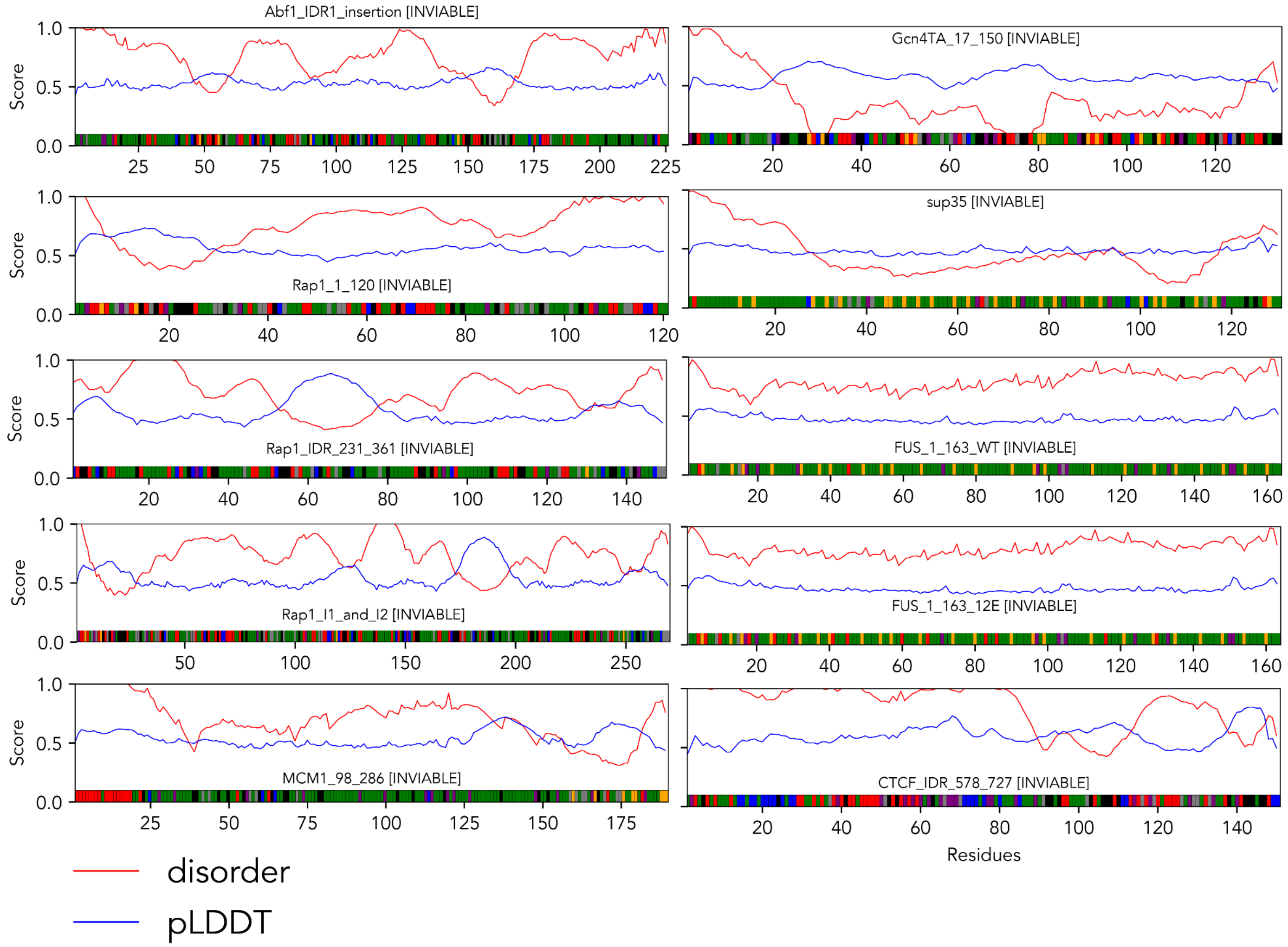

Supplementary Figure 4. Linear sequence analysis of inviable IDRs. We analyzed linear sequence profiles for IDRs taken from the yeast proteins Abf1, Gcn4, Rap1, Sup35, and Mcm1. In addition, we examined IDRs taken from the human proteins FUS and CTCF. The per-residue disorder score (red) and predicted pLDDT score (blue) provide linear sequence descriptions. The amino acid composition is shown at the bottom of each panel, based on amino acid chemistry. Acidic residues are red, basic residues are blue, polar residues are green, aliphatic hydrophobic residues are black, and prolines are purple.

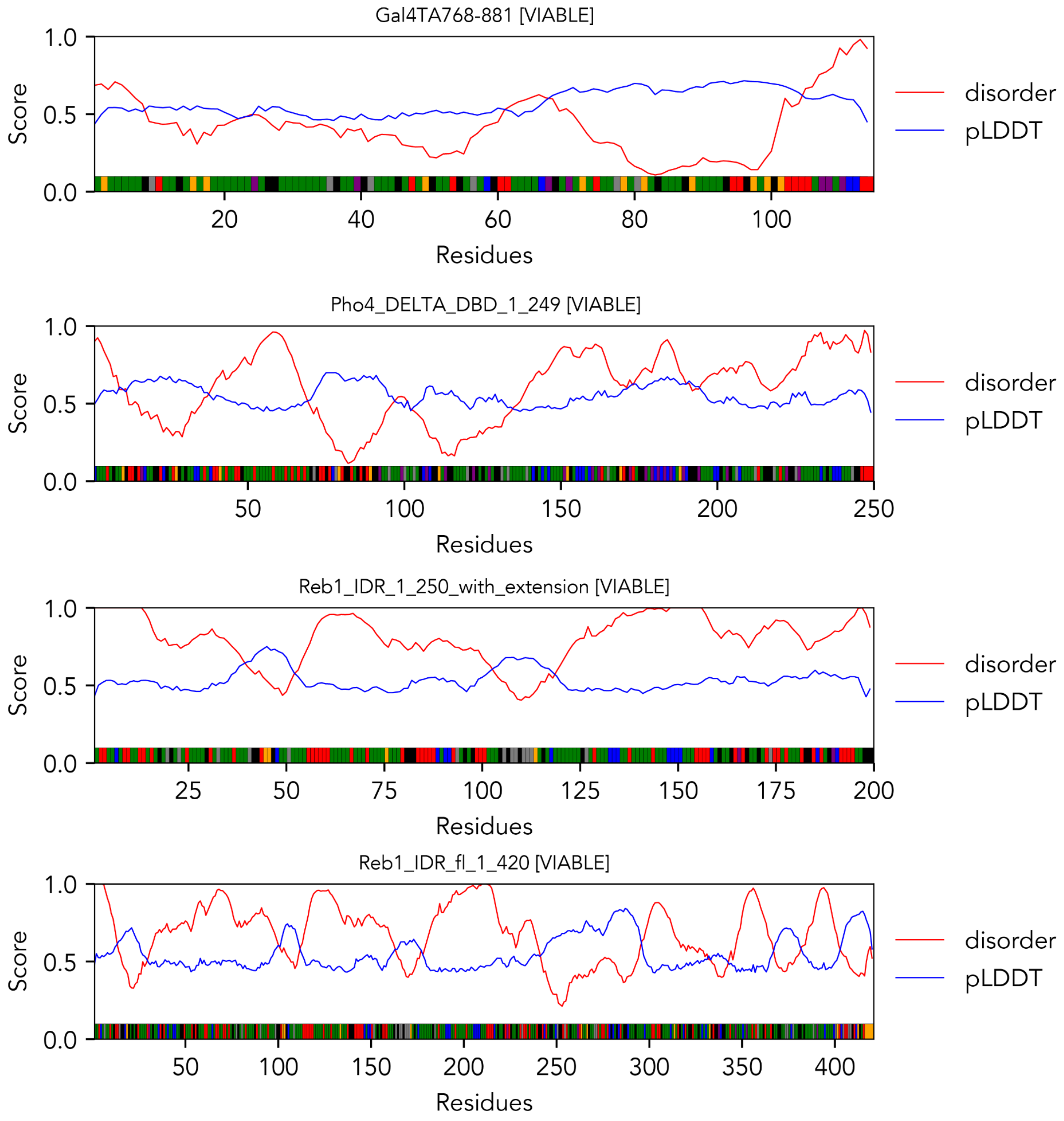

Supplementary Figure 5. Sequence profiles for viable IDRs. Linear sequence analysis for IDRs from yeast proteins Gal4, Pho4, and Reb1. The analysis here is analogous to **Fig. S4**. Viable sequences range from 113 residues (top) to over 400 residues (bottom), are largely predicted to be disordered, and contain a similar fraction of acidic, polar, and hydrophobic residues as Abf1-IDR2.

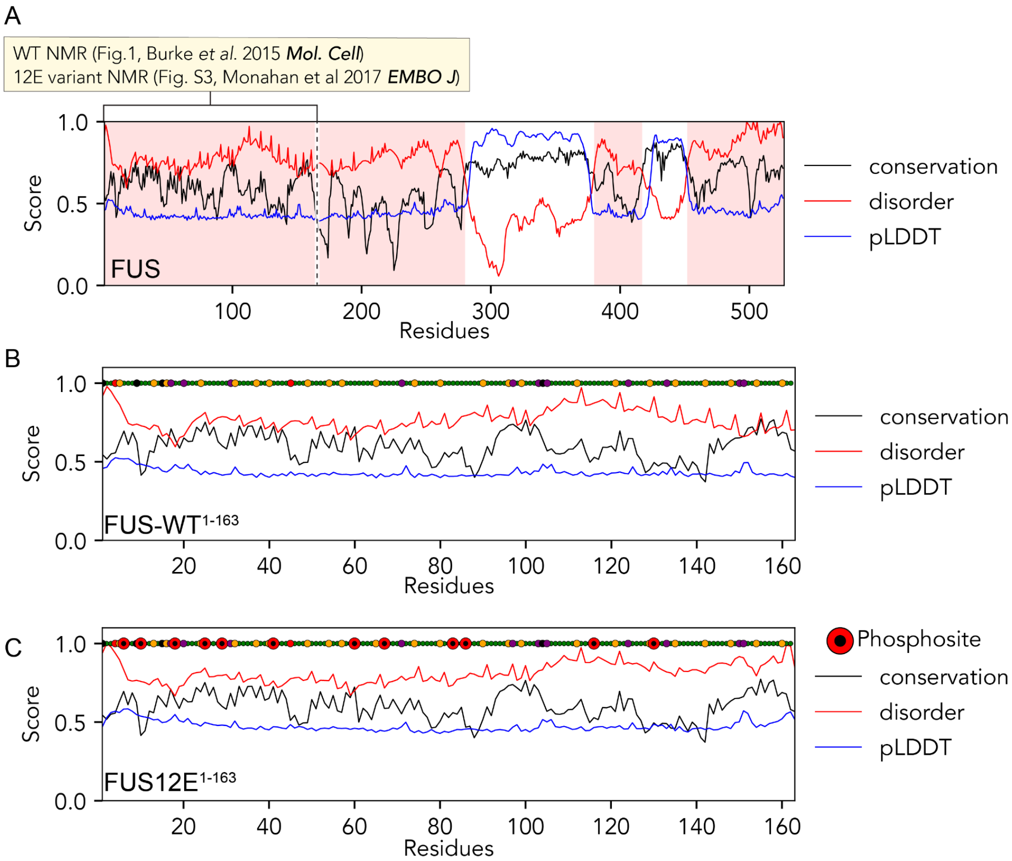

D

MASNDYEQQAEQSYGAYPEQPGQGYEQQSEQPYGQQSYSGYEQSTDTSGY
GQSSYSSYGQEQNTGYGEQSTPQGYGSTGGYGSEQSEQSSYGQQSSYPGY
GQQPAPSSTSGSYGSSEQSSSYGQPQSGSYEQQPSYGGQQQSYGQQQSYN
PPQGYGQQNQYNS

Supplementary Figure 6. FUS$^{1\text{-}163}$ and FUS12E$^{1\text{-}163}$ are low complexity disordered regions, as characterized by both experimental and computational approaches. (A) Full-length protein with IDRs highlighted in red (identified using both disorder scores and pLDDT scores). Residues 1-163 are relatively poorly conserved, and based on NMR data collected on both WT and the 12E phosphomimetic version, are predicted to be fully disordered (references and associated figures shown in the inset). **(B)** FUS$^{1\text{-}163}$ WT sequence and **(C)** FUS12E$^{1\text{-}163}$ regions zoomed in with residues from different physicochemical groups illustrated by color. Green = polar (S/T/G/Q/N/H/C), red is acidic (E/D), blue is basic (R/K), orange is aromatic (Y/W/F), black is aliphatic (A/I/L/V/M), and purple is proline. **(D)** Complete FUS12E$^{1\text{-}163}$ sequence.

A

	Sequence	FCR	NCPR	Hydro.[a]	Aro
Gal4[G4]	FNTTTMDDVYNYLFDDE	0.294	-0.294	3.694	4
Gal4[M2]	FMPRDALHGFDWSEEDD	0.412	-0.294	3.365	3
p65[M1]	DEDFSSIADMDFSALLS	0.294	-0.294	4.647	2
GR[M1]	VSLLEVIEPEVLYAGYD	0.235	-0.235	5.141	2
VP16[M1]	GEDVAMAHADALDDFDL	0.353	-0.353	4.447	1
GCN4[M1]	ESFFSSSTDSTPMFEY	0.188	-0.188	3.969	4

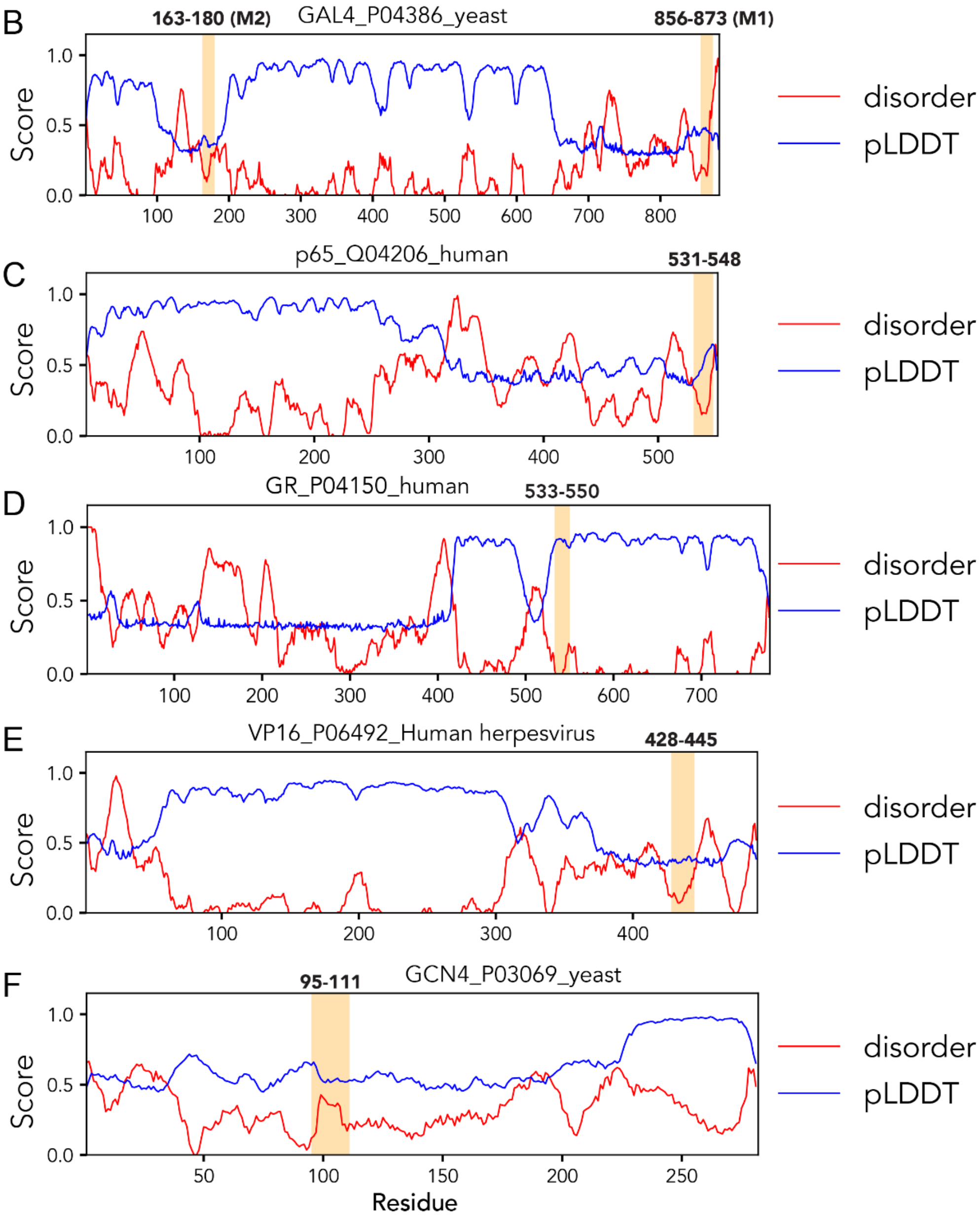

Supplementary Figure 7. Compositionally-selected 16-17 residue subsequences from unrelated proteins. Subsequences were selected from Gal4, p65, GR, VP16, and Gcn4. **(A)** Subsequences were selected solely based on composition and size. For each protein, the gene name, UniProt ID, and species of origin are provided. **(B-F)** Relative positions of subsequences within their associated full-length proteins, along with per-residue disorder scores and per-residue predicted pLDDT scores. Given that the selection was based

solely on composition within a sliding window, the motif from GR falls within a folded region, which we retained as a convenient control, since this region cannot have evolved to function as a binding motif in a disordered protein region.

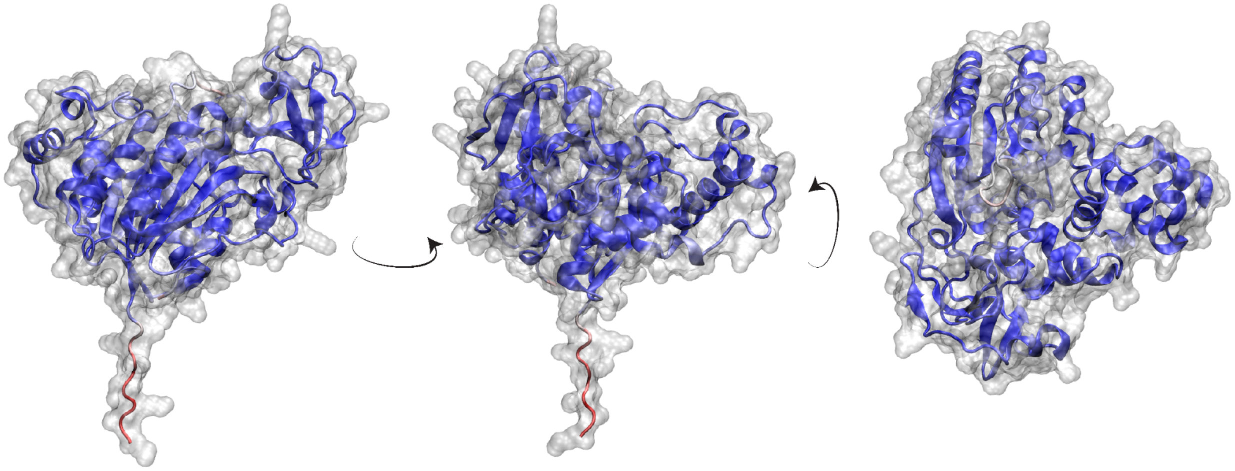

Supplementary Figure 8. ODM-seq is conducted using the M.SssI CpG-specific methyltransferase (UniProt ID: P15840). Shown here is the predicted AlphaFold structure of the enzyme. Structure is colored by pLDDT (blue = high, red = low). Purification protocol described in *Methods* and **Fig. S10, S11**.

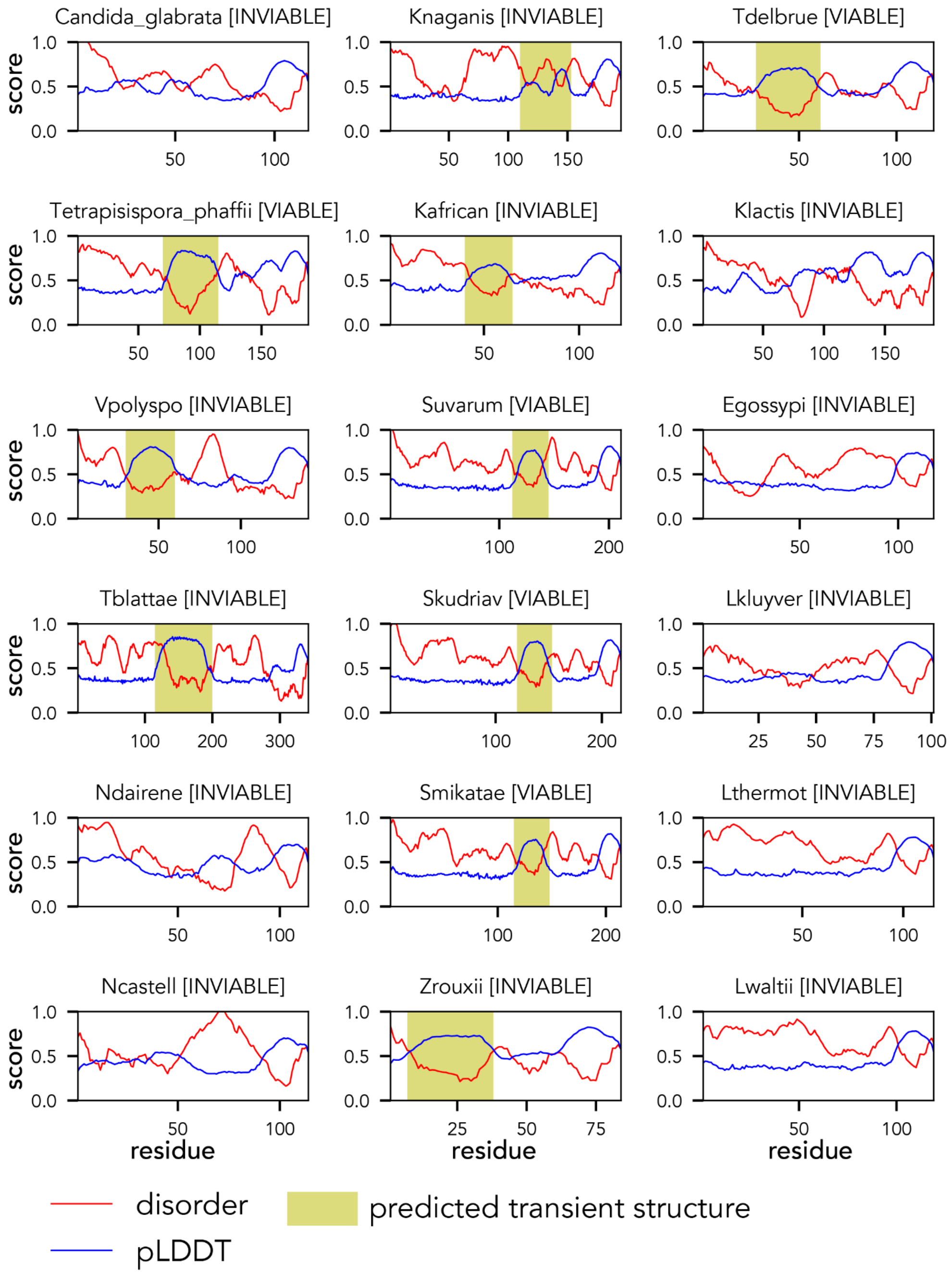

Supplementary Figure 9. Abf1 orthologs IDR2 analysis. All viable orthologous sequences contain a predicted structured region that we interpret as a local helical region (highlighted in yellow). However, several inviable sequences also possess a predicted structured subregion, suggesting this is insufficient for viability. The fact that a helical sub-region is present and absent across orthologs is consistent with a model in which rapid evolutionary changes in IDRs can drive the gain and loss of SLiMs within transient helical regions. However, we emphasize that we do not know if these predicted transient helices harbor SLiMs.

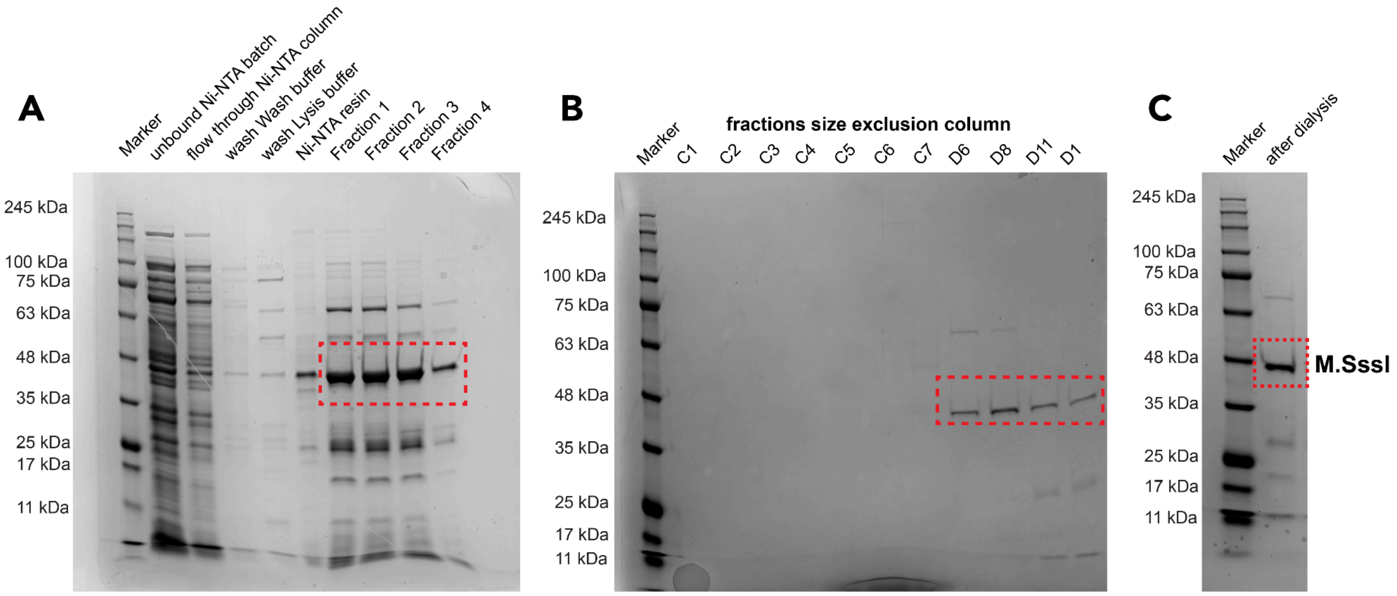

Supplementary Figure 10. Purification of M.SssI. (A), **(B)**, **(C)** show 10% SDS-PAGE (Serva) analysis of the indicated steps and fractions during the purification of M.SssI (*Methods*). Red boxes highlight the bands corresponding to M.SssI (42 kDa). Marker lane (M): Triple Color Protein Standard III (39258, Serva).

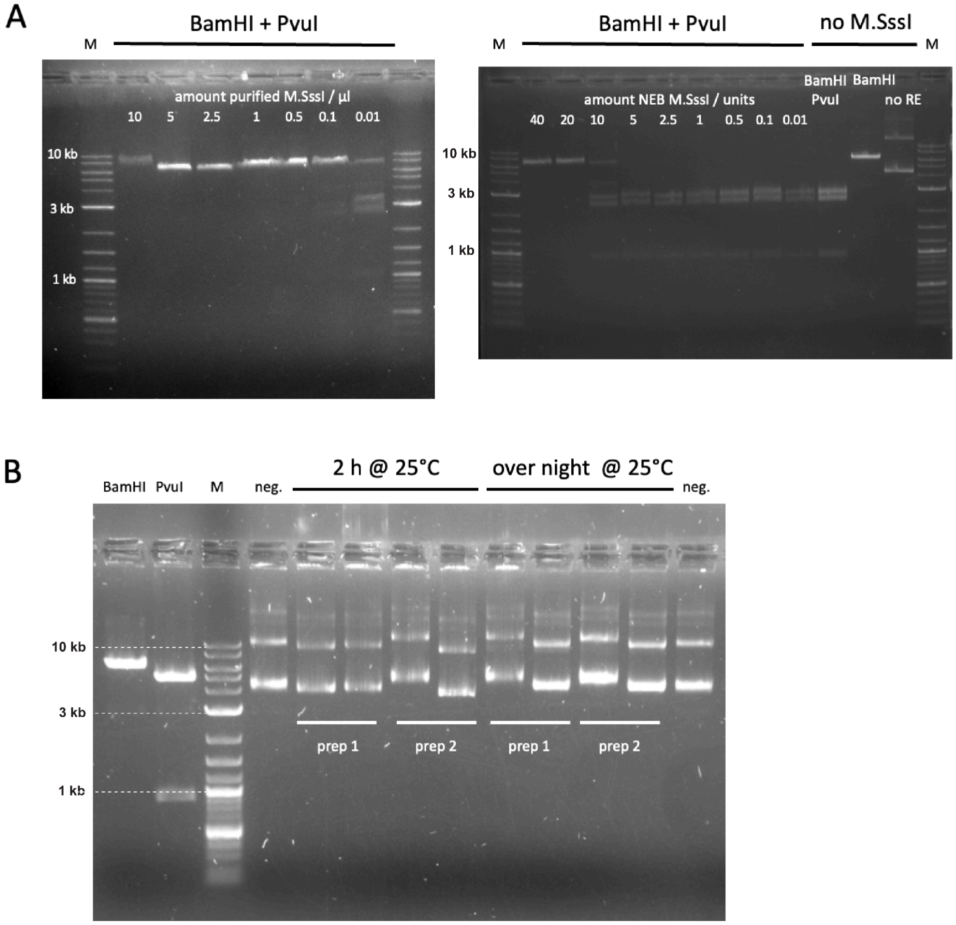

Supplementary Figure 11. Characterization of purified M.SssI. (A) Comparison of DNA methylation activity at CpG sites between our purified (left) and commercial (NEB, right) M.SssI. After plasmid pUC1-ftz (5769 bp) was incubated with indicated amounts of M.SssI in the presence of SAM and purified, it was digested with CpG methylation-non-sensitive BamHI (unique site: linearization) or BamHI + CpG methylation-sensitive PvuI (2544, 2329, 896 bp fragments if not blocked by CpG methylation). DNA fragments were electrophoresed in 1% agarose 1xTAE gels. 0.5 µl of the here-shown M.SssI preparation had similar activity to 20 units of the commercial M.SssI, i.e., a concentration of 40 U/µl. Marker lane: 1 kb plus ladder (NEB). **(B)** Purified M.SssI preparations (prep 1 and prep 2) were free from nuclease contamination as they did not remove the supercoiled form of plasmid pUC19-ftz even after overnight incubation in two different buffers, NEB rCutSmart and NEB Buffer 2, in the left and right lanes, respectively, for each test reaction of prep 1 and 2.

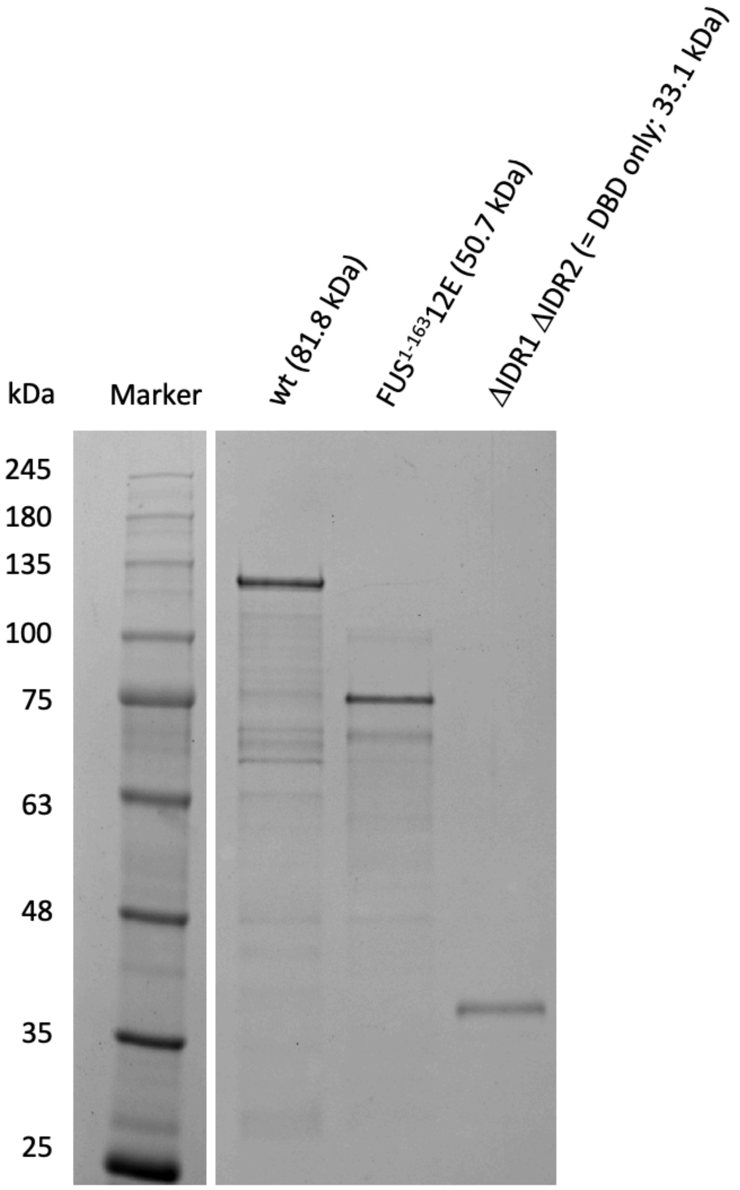

Supplementary Figure 12. 10% SDS-Page (Serva) analysis of indicated WT or variant Abf1 preparations. Marker: Triple Color Protein Standard III (39258, Serva)

A

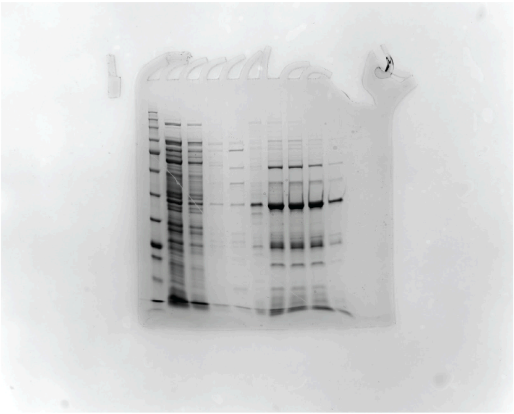

B

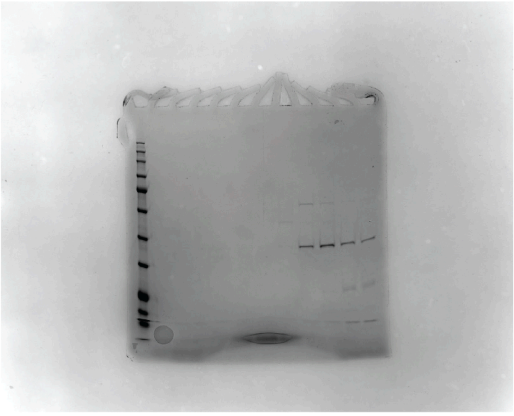

C

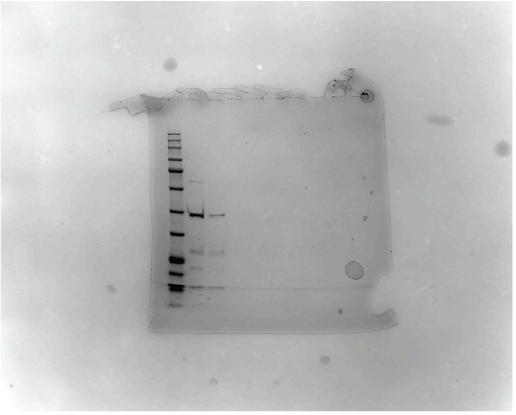

Supplementary Figure 13. Uncropped gels from **(A)** Fig. S10A, **(B)** Fig. S10B, **(C)** Fig. S10C.

A

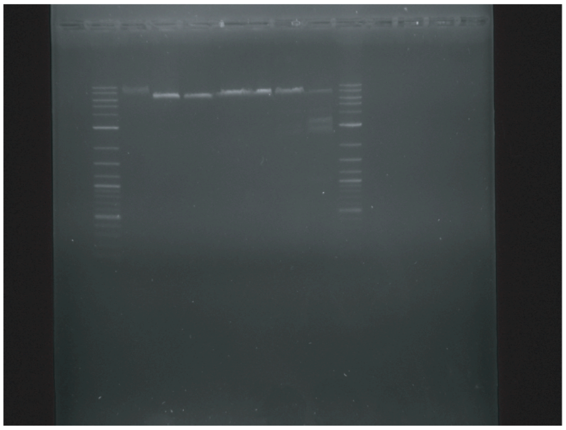

B

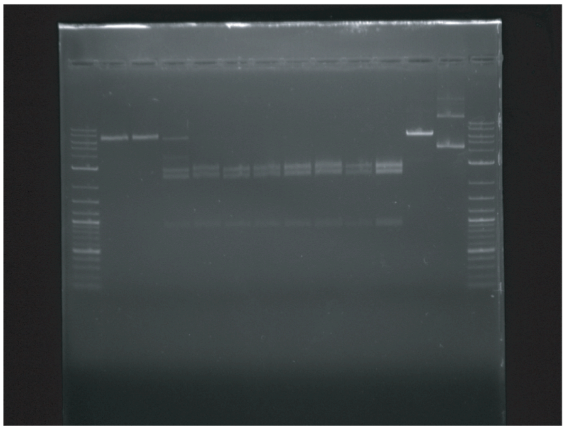

C

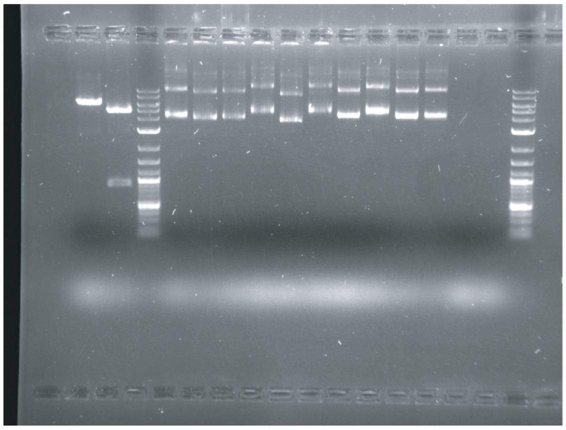

Supplementary Figure 14. Uncropped gels from **(A)** Fig. S11A (left), **(A)** Fig. S11A (right), **(A)** Fig. S11B

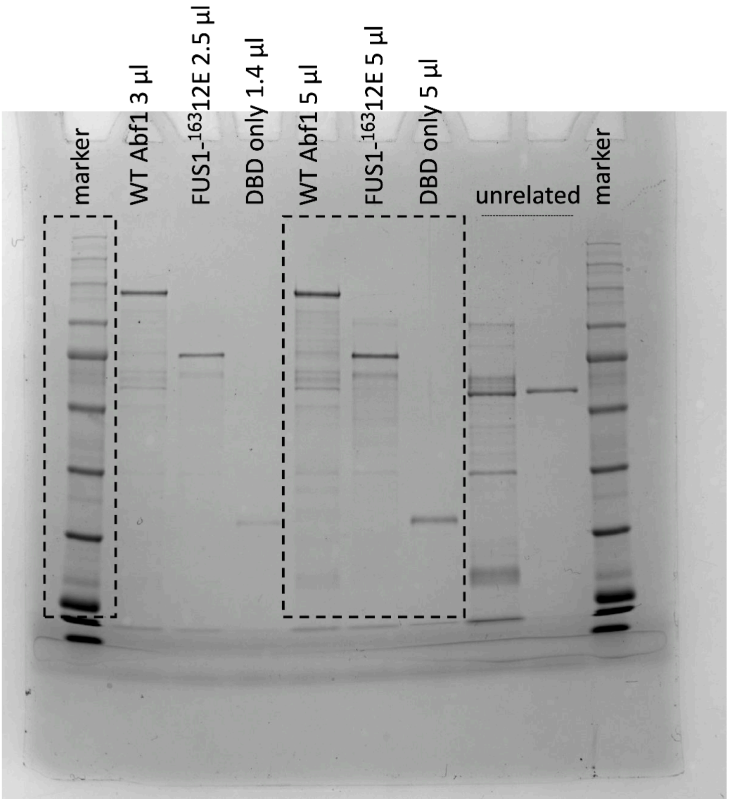

Supplementary Figure 15. Uncropped gel from Fig. S12. Boxes show lanes in Fig. S12.
